# Complementarity of medium-throughput *in situ* RNA hybridization and tissue-specific transcriptomics: case study of Arabidopsis seed development kinetics

**DOI:** 10.1038/srep24644

**Published:** 2016-04-20

**Authors:** Edith Francoz, Philippe Ranocha, Clémentine Pernot, Aurélie Le Ru, Valérie Pacquit, Christophe Dunand, Vincent Burlat

**Affiliations:** 1 Laboratoire de Recherche en Sciences Végétales, Université de Toulouse, CNRS, UPS, 24 chemin de Borde Rouge, Auzeville, BP42617, 31326 Castanet Tolosan, France; 2 Fédération de Recherche 3450, Plateforme Imagerie, Pôle de Biotechnologie Végétale, Castanet-Tolosan 31326, France

## Abstract

The rationale of this study is to compare and integrate two heterologous datasets intended to unravel the spatiotemporal specificities of gene expression in a rapidly growing and complex organ. We implemented medium-throughput RNA *in situ* hybridization (ISH) for 39 genes mainly corresponding to cell wall proteins for which we have particular interest, selected (i) on their sequence identity (24 class III peroxidase multigenic family members and 15 additional genes used as positive controls) and (ii) on their expression levels in a publicly available *Arabidopsis thaliana* seed tissue-specific transcriptomics study. The specificity of the hybridization signals was carefully studied, and ISH results obtained for the 39 selected genes were systematically compared with tissue-specific transcriptomics for 5 seed developmental stages. Integration of results illustrates the complementarity of both datasets. The tissue-specific transcriptomics provides high-throughput possibilities whereas ISH provides high spatial resolution. Moreover, depending on the tissues and the developmental stages considered, one or the other technique appears more sensitive than the other. For each tissue/developmental stage, we finally determined tissue-specific transcriptomic threshold values compatible with the spatiotemporally-specific detection limits of ISH for lists of hundreds to tens-of-thousands of genes.

The post-genomics era leads to the generation of increasing amount of publicly available large transcriptomic datasets. In October 2015, 3,848 array- and sequence-based datasets covering 1,604,299 samples from all living kingdoms were deposited on the gene expression omnibus (http://www.ncbi.nlm.nih.gov/geo/). Transcriptomics can reach cellular/tissular levels using accurate sampling methods. These include for example fluorescence activated cell sorting (FACS) of animal stem cells^1^ or plant protoplast populations^2^,^3^, isolation of nuclei tagged in specific cell types^4^, or laser capture microdissection (LCM) of single prokaryotic cell^5^, functionally distinct human neurons^6^ or plant tissue serial sections^7^,^8^,^9^,^10^. Spatiotemporally-specific transcriptomes are accessible through raw data files or user-friendly web-based tools^6^,^11^,^12^,^13^. These tissue-specific transcriptomic studies need validation and refinement of the expression profiles through qRT-PCR, promoter::reporter genes or RNA *in situ* hybridization (ISH). The most popular ISH protocols use whole mount permeabilized samples^14^,^15^ or paraplast-embedded sections^16^,^17^. ISH is a powerful approach often underestimated probably due to its perception as being difficult to perform, its relative sensitivity and its moderate throughput. Rare reports of elegant medium-to-high throughput ISH studies exist^3^,^18^ but with relatively poor spatiotemporal resolution.

A biological question that particularly needs spatiotemporally-resolved gene expression data concerns the dynamics of plant cell walls. Their study is of high interest since they constitute an active zone of molecular dialogue and signalling during plant growth and plant microorganism interactions^19^, and they harbour stocks of renewable carbon of high interest for pulp and paper and biofuel production^20^,^21^. However, plant cell wall study is complex since they form diverse, complex and dynamic networks with highly cell-specific patterns as illustrated by numerous immunocytochemical studies of cell wall polymers such as polysaccharides and lignins^22^,^23^,^24^,^25^,^26^,^27^,^28^. The knowledge of plant cell wall proteomes has increased to about 2,000 proteins in a given organism in the last ten years and these proteomic studies argue for species- and organ-specific profiles^29^,^30^,^31^. High-throughput plant cell-specific profiles remain so far unachievable and these proteomic studies have to be completed by more spatially resolutive approaches such as immunolabelling dedicated to a subset of proteins^30^.

The present study uses the fast and complex development kinetics of the ~400 μm-long seeds from the model plant *Arabidopsis thaliana* (Supplementary Fig. S1) well adapted to the study of cell wall dynamics^25^. The rationale of the present study was to evaluate and demonstrate the complementarity between the results obtained from a publicly available high-throughput tissue-specific transcriptomic study^7^ (Supplementary Table S1) and our medium-throughput ISH analysis implemented for 39 selected genes and 6 seed developmental stages. We primarily selected 24 genes of the multigenic family of Class III peroxidases (73 members) mainly corresponding to cell wall proteins^32^. Recent studies demonstrated that these oxidoreductases that may accommodate a wide range of substrate *in vitro* became highly specific *in vivo* due to their accurate spatiotemporal expression and positioning in cell wall microdomains where they encounter specific partners and substrates^33^,^34^. Studying members of such a multigenic family also allowed to clearly assess the specificity of ISH signals given the high sequence identities. To reinforce our gene sampling, we added 15 genes mostly corresponding to cell wall proteins primarily selected based on their tissue-specific transcriptomic expression values.

Tissue-specific transcriptomic high-throughput was not challenged by ISH medium-throughput, however, ISH allowed to obtain specific signals for genes with high sequence identity that were not distinguished by tissue-specific transcriptomics. We also proved that ISH was more accurate than tissue-specific transcriptomics regarding the localisation of the transcripts in precise cell types. Our results were discussed and compared with the literature describing microphenotypes or cell-specific expression data for some of these genes. For all other genes, our results constitute a basis for future functional studies. To widen the interest of our method, we finally estimated, for each tissue/developmental stage, thresholds of tissue-specific transcriptomic expression values for which a specific ISH signal could be reasonably detected for lists of hundreds to tens-of-thousands of genes.

## Methods

**Figure 1:**
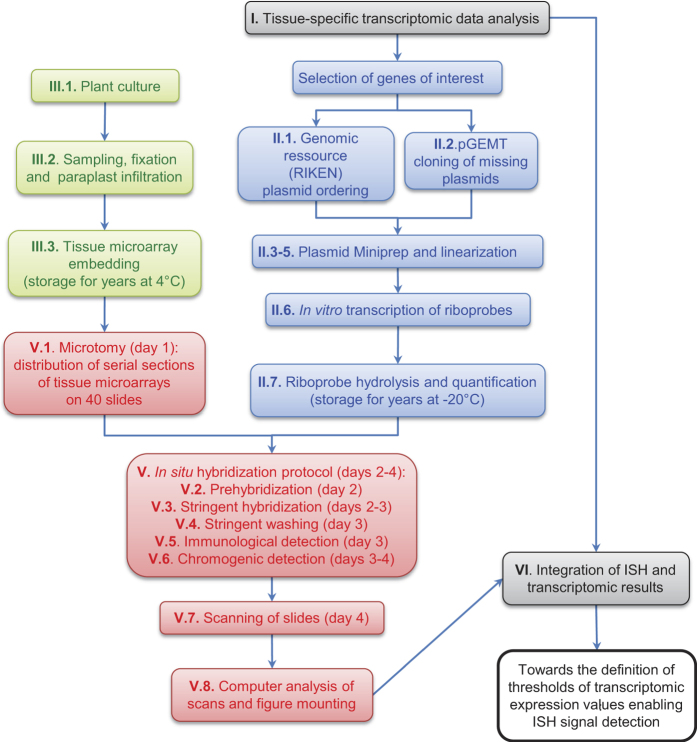
Flowchart of the method used for medium throughput *in situ* hybridization based on/compared to published tissue-specific transcriptomic data. This flowchart is labelled according to the paragraph numbers appearing in the Material and Methods and Supplementary Methods sections.

The overall flowchart of the method is presented in Fig. 1. The following sections explain the principle of the 6 main steps of the method displayed in Fig. 1. Extended details of the methods and step-by-step protocol are provided in the Supplementary Method in the same order.

### Tissue-specific transcriptomic data analysis (step I)

Gene Expression Omnibus accession series GSE12404^7^ was downloaded as series matrix files at www.ncbi.nlm.nih.gov/geo. The “.txt” file was converted to “.xls” file which was further annotated and edited (Supplementary Table S1). The expression data of the 73 Class III peroxidases (*AtPRXs*)^35^,^36^ (http://peroxibase.toulouse.inra.fr/) was filtered (Supplementary Table S2). We first selected the 16 most highly expressed *AtPRXs* spot IDs from this resource and from two additional resources (seed eFP browser at http://bar.utoronto.ca/efp/cgi-bin/efpWeb.cgi?dataSource=Seed^12^, and the Supplementary data 2 provided in the original publication^7^). We performed a Pearson correlation coefficient analysis showing that, despite different data treatment, the three resources corresponded to the same original data (Supplementary Table S3). The *circa* 3 ratio of signal intensities between the GSE12404/eFP browser datasets and the Belmonte *et al.* dataset (Supplementary Table S3) allowed to convert the detection limit value of 15 defined in the original publication^7^ to a detection limit value of 45 for the GSE12404/eFP browser datasets. In the following, we used the most complete GSE12404 dataset (Supplementary Table S1). For the ISH study, we selected 24 *AtPRXs* (about 1/3 of the family) corresponding to the 16 most responsive spot IDs (14 unique genes and 2 ambiguous duplicated gene pairs), to 6 *AtPRXs* whose maximum transcriptomic expression value was below a 300 arbitrary cut-off and 2 *AtPRXs* for which no spot ID was available (Supplementary Table S2). We added 15 non-peroxidase genes, that corresponded to two peroxidase partners (*RBOHE*, *RBOHF*), one housekeeping gene (*TUB4*), and 12 putative cell wall proteins in which we have particular interest^29^,^31^. We paid attention to select candidates presenting maximum tissue-specific transcriptomic expression values ranging from very high to medium levels (Supplementary Table S5).

The most recent release of the *Arabidopsis* cDNA sequences dataset (TAIR10) was downloaded from The Arabidopsis Information Resource FTP server (ftp://ftp.arabidopsis.org/home/tair/Sequences/blast_datasets/TAIR10_blastsets/). Each sequence of this dataset was searched locally using Blastall 2.2.24 (“blastn” command with the default parameters^37^) against the TAIR10 cDNA database. The second best hit (defined as the best hit beside the query itself) for each of the 33,602 cDNA sequences of TAIR10 is indicated in Supplementary Table S6. The Supplementary Table S7 corresponds to the extraction from the Supplementary Table S6 of the second best hit of the 15,866 cDNAs with a nucleotide number above 300. The Supplementary Table S8 corresponds to the extraction from Supplementary Table S7 of the second best hit among the 73 *AtPRXs* and the 15 additional non peroxidase genes selected for our *in situ* hybridization study. More detailed protocols are provided in Supplementary Methods.

### Plasmid resources and riboprobe *in vitro* transcription (step II)

The full length cDNA (pda clones) ordered at the RIKEN bioresource center [ http://www.brc.riken.jp/lab/epd/catalog/cdnaclone.html;^38^,^39^] are listed in the Supplementary Table S9. These cDNA clones are provided within pBluescript-derived vector series allowing their direct utilization for *in vitro* transcription of riboprobes since the multiple cloning sites are framed by the T3 and T7 RNA polymerase promoters, respectively (Supplementary Fig. S2). Five cDNAs not available at RIKEN were amplified using a pool of retro-transcribed mRNAs from various seed developmental stages, and cloned in pGEM-T Easy (Promega) (Supplementary Table S10). These clones are included in the Supplementary Table S9, and the multiple cloning site (MCS) including SP6 and T7 RNA polymerase promoter position is shown in Supplementary Fig. S2.

Plasmids (5–7 μg) were linearized with excess (20 units) of single cut 5′ overhang or blunt restriction enzymes (Promega, Roche) (Supplementary Table S9) for 4 h at 37 °C. The digestion occurred either at the 5′ or the 3′ end of the cDNA, in order to produce the template for *in vitro* transcription of the antisense or the sense probes, respectively. Sense probes were used as classical negative control and the significance/limits of this type of control and the use of additional negative controls will be discussed hereafter. Whenever possible, the linearization was performed using enzymes single-cutting in the multiple cloning site (MCS) and not cutting the cDNA (Supplementary Fig. S2). In some cases, we used enzymes single-cutting at the cDNA ends and not cutting the vector (Supplementary Fig. S3).

*In vitro* transcription of Digoxigenin (Dig)-labelled riboprobes for ISH or unlabelled antisense riboprobes for competitive inhibition was performed during 2 h at 37 °C, using Dig RNA labelling mix (Roche) and unlabelled NTP mix (Promega), respectively, and T3, T7 or SP6 RNA polymerase (Promega) (Supplementary Table S9). Riboprobes were further chopped to an average size of 400 b by alkaline hydrolysis (Supplementary Table S9). Step-by-step detailed protocol is provided in Supplementary Methods.

### Plant material and tissue microarray preparation (step III)

The same wild-type *Arabidopsis thaliana* ecotype (Wassilewskija, Ws) and similar culture conditions (115 μmol.m^−2^.sec^−1^ continuous light using 25% Osram 58 W Fluora and 75% lumilux cool daylight 58 W tubular fluorescent lamps, respectively; 22 °C; 75% relative humidity) as used for the tissue-specific transcriptomic reference study^7^ was primarily used. For specificity studies, *atprx36* T-DNA knock out insertion line [per36-1; SAIL_194_G03^33^;] and its corresponding *A. thaliana* wild type background (Columbia 0, Col-0) were cultivated in the same conditions. RNase-free conditions were strictly observed for all steps. In order to cover the whole kinetics of seed development, 39, 40, 43 and 45-day old plants were sampled each in three batches (siliques from the top, medium and bottom of the floral stem, the youngest siliques being at the top). In each case, dozens of whole siliques were harvested by cutting the pedicel with a razor blade and rapidly vacuum infiltrated in FAA (10% Formalin (37% formaldehyde solution, Sigma-Aldrich); 50% ethyl alcohol; 5% acetic acid; 35% DEPC-treated water) and fixed for 16 h at 4 °C. The dehydration and paraplast infiltration protocol was adapted from^40^. Siliques were concentrated in embedding molds constituting tissue microarrays emcompassing up to 1,000 developing seeds (Supplementary Fig. S1b). Step-by-step detailed protocol is provided in Supplementary Methods.

### Medium-throughput *in situ* hybridization conditions (step IV)

The respect of RNase-free conditions is crucial for the success of ISH. The detail of material, chemicals and solutions including preparation guide is provided in Supplementary Methods.

### Medium-throughput *in situ* hybridization (step V)

**Figure 2:**
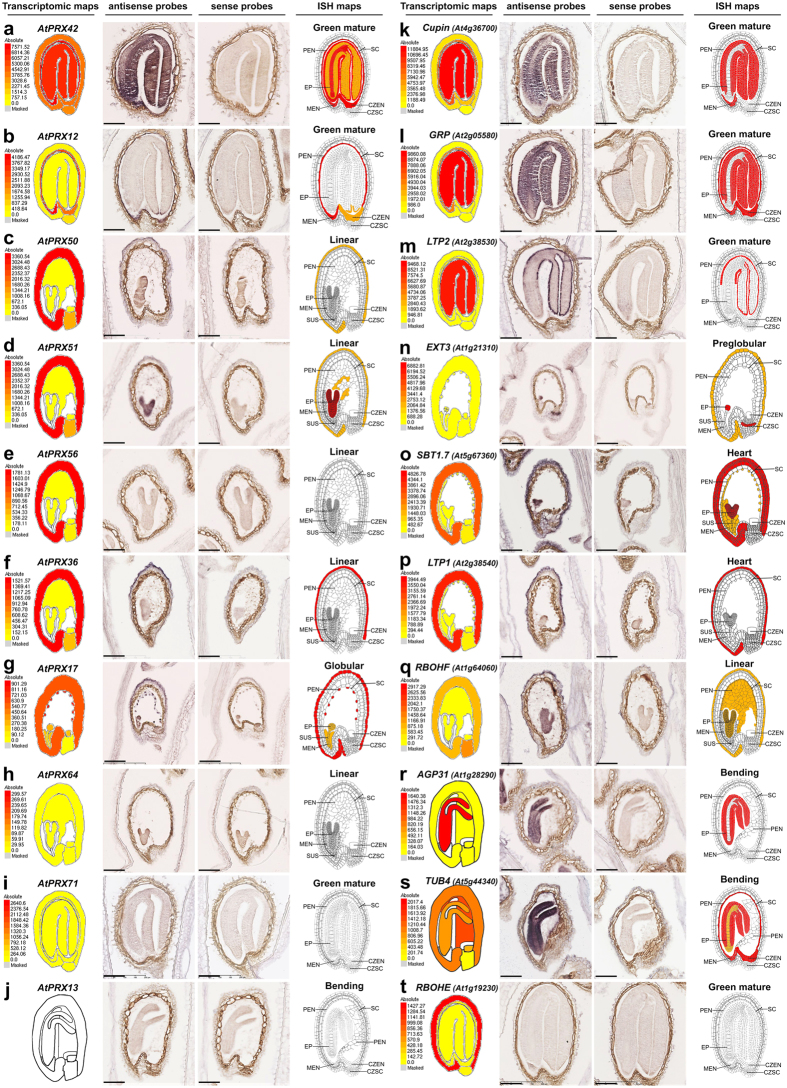
Illustration of the *in situ* hybridization results obtained for a selection of genes and developmental stages. Results for 10 *AtPRXs* (**a**–**j**) and for 10 non peroxidase genes (**k**–**t**) are displayed in the decreasing order of their maximal individual tissue-specific transcriptomic expression values^7^. For each gene and each developmental stage, a screen copy of the seed eFP browser tissue-specific transcriptomic map including the individual absolute heatmap scale (red-to-yellow colour codes correspond to high-to-low expression values) that is different for each gene^12^ is compared with the corresponding ISH results (for both the antisense and sense probes). The final corresponding new ISH map (re-coloured after original drawings from ref. 7 available at Seedgenenetwork http://estdb.biology.ucla.edu/seed/) gave increased cellular resolution with a unique colour code for all genes (red corresponding to visually detected strong signals, orange corresponding to moderate signals and white corresponding to the absence of detected ISH signal). Note that the naturally brown colour of seed coat does not correspond to ISH signal. The full ISH results corresponding to a selection of 5 developmental stages for all 39 genes can be found in Supplementary Figs. S4–S42. Scale bars: 100 μm. Abbreviations: AS, anti-sense; CZEN, chalazal endosperm; CZSC, chalazal seed coat; EP, embryo proper; MEN, micropylar endosperm; PEN, peripheral endosperm; S, sense; SC, seed coat; SUS, suspensor.

The basis of this protocol comes from^41^ following several adaptations^40^,^42^,^43^ and final simplification using a unique buffer for most of the steps and substituting the acetic anhydre/triethanolamine tedious charge equilibration step by a more simple diethylpyrocarbonate (DEPC)-mediated carbethoxylation step, inspired by^16^. 10 μm-serial sections of tissue microarrays were disposed on precoated microscopy slides. 40 slides, corresponding to 40 riboprobes were processed in one ISH experiment. Hybridization was performed overnight at 50 °C, and following stringent washing steps, hybridized probes were immunodetected using anti-Dig-alkaline phosphatase (AP) antibody (Roche) and colour development of AP reaction was performed overnight at room temperature. Step-by-step detailed protocol is provided with timing in Supplementary Methods. Slides were mounted in Eukitt and scanned at high-throughput using a nanozoomer HT slide scanner (Hamamatsu). We routinely scanned all slides at ×20 focus (=0.46 μm per pixel on a single z plan) and these scans were viewed and analysed using the NDP view freeware (Hamamatsu) and directly used to prepare Figures (Fig. 2; Supplementary Figs. S4–S45).

### Integration of ISH and tissue-specific transcriptomic results (step VI)

One Figure was assembled for each gene studied by ISH. In each Figure, we compared the screen copy of the individual tissue-specific transcriptomic maps and relative heatmap scale available through seed eFP browser at http://bar.utoronto.ca/efp/cgi-bin/efpWeb.cgi?dataSource=Seed^12^ for 5 selected developmental stages, with corresponding ISH antisense and sense images directly extracted from NDP view. Finally, we constructed using Corel Photopaint a new detailed expression map for each gene based on our ISH results, with a red/orange/white colour code (red, strong ISH signals; orange, moderate ISH signals; white, no ISH signal), and using the original detailed cartoon from ref. 7 available at Seedgenenetwork (http://estdb.biology.ucla.edu/seed/) as a basis for the drawing except for the bending cotyledons stage for which we drew our own cartoon. Note that the new ISH maps corresponded to the observation of numerous seed sections from at least 3 experimental repeats and not only to the individual displayed ISH images.

The Supplementary Table S1 was used to extract tissue-specific transcriptomic expression profiles from all the *AtPRXs* and the 39 genes studied in ISH. This data was gathered in single Microsoft Excel files (Supplementary Table S4 for peroxidases and Supplementary Table S5 for the 39 genes studied by ISH), keeping the original information about the maximum expression value and the ranking position of each gene. The individual tissue-specific transcriptomic values were framed in bold when either strong or moderate ISH signal was observed in the corresponding tissues according to Supplementary Figs. S4–S42. The gene annotation was framed in red (strong ISH signal in at least one tissue), orange (moderate-to-low ISH signal in at least one tissue) or yellow (no ISH signal detected) following the similar colour code as in Supplementary Figs. S4–S42. Demonstration of ISH specificity is provided in Supplementary Figs. S43–S45.

**Figure 3:**
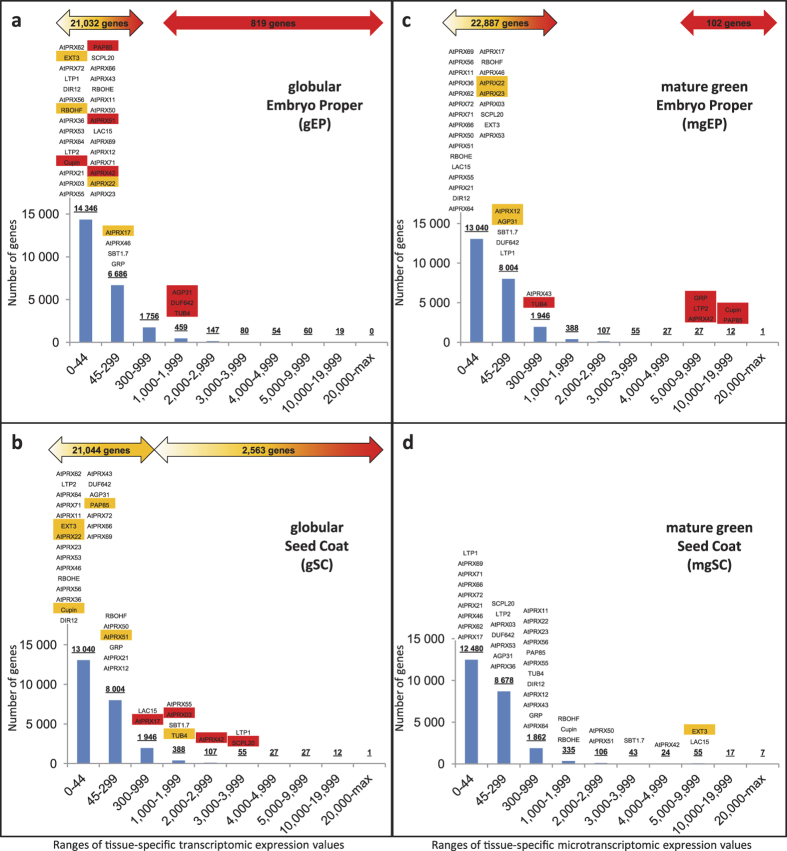
Thresholds of tissue-specific transcriptomic values compatible with ISH are specific to each developmental stage/tissue. For each developmental stage/tissue tissue-specific transcriptomic sample ((**a**,**b**) globular developmental stage; (**c**,**d**) mature green developmental stage; (**a**,**c**) embryo proper tissue; (**b**,**d**) seed coat tissue), the 23,933 genes present on the microarray were classified according to their tissue-specific transcriptomic expression values. The range 0–44 corresponded to the expression values below the 45 detection limit of the transcriptomic study (55–65% of the genes); the range 45–299 corresponded to the expression values between the 45 detection limit of the transcriptomics and the 300 arbitrary cut-off that we initially defined for our ISH study; the other ranges were arbitrarily set to allow the distribution of the genes in various expression value groups. The resulting number of genes within each range was plotted on individual histograms. All the genes analysed by ISH in this study were positioned above the histograms according to their individual tissue-specific transcriptomic expression value. The ISH results were colour-coded in red/orange/white as in Fig. 2, according to Supplementary Figs. S4–S42. The deducted total number of genes compatible, to various extend, with ISH was posted on the top of each graph within double arrows using shades of the same colour coding. This clearly illustrates that the sensitivity of ISH is dependent on spatiotemporal parameters and the complementarity of transcriptomics and ISH. The corresponding histograms for all developmental stages and tissues are available in Supplementary Figs. S46–S51.

The Supplementary Table S1 was sequentially used to distribute, for each of 36 tissue-specific transcriptomic samples (all samples except the 6 whole seed samples), the 23,933 genes within 10 groups according to their transcriptomic expression value. The groups corresponded to expression values of 0–44 (below the detection limit of the transcriptomic study; 55–65% of the genes depending on the sample), 45–299 (below the arbitrary 300 cut-off used for our first selection of candidate genes), 1000–1999, 2000–2999, 3000–3999, 4000–4999, 5000–9999, 10000–19999, 20000-max. In order to fully integrate our ISH results with the whole tissue-specific transcriptomic data, the abbreviation of the 37 genes of the 39 genes studied by ISH that were present on the array (all 39 genes except *AtPRX13* and *AtPRX32*) was positioned on the individual histograms on the top of the range corresponding to their tissue-specific transcriptomic value (Fig. 3; Supplementary Figs. S46–S51). In order to summarize the ISH results, we kept the same red/orange/white colour code described above. Less genes were studied in ISH for the preglobular stage which was under-represented on the tissue arrays. We finally calculated within colour-coded double arrows positioned on the top of the histograms, the sum of genes for which ISH could be sensitive enough to various degrees for each tissue/developmental stage.

## Results and Discussion

### Comparison and integration of *in situ* hybridization and tissue-specific transcriptomic results

We systematically compared, for each of the 39 genes and 5 selected developmental stages, a screen copy of an user-friendly web-based tissue-specific transcriptomic map^12^ including the individual absolute heatmap scale that is different for each gene, with the corresponding ISH results (Fig. 2a–t; Supplementary Figs. S4–S42). All together, the analysis of ISH results for tens of thousands of seed sections allowed drawing the corresponding new ISH maps giving increased cellular resolution, using a unique colour code for all genes; red, orange and white corresponding to strong, moderate and no visually detected ISH signal, respectively (Fig. 2a–t; Supplementary Figs. S4–S42). Since preglobular stage was underrepresented on the tissue microarray, only a subset of the 39 genes could be studied at this developmental stage. Additionally, in some circumstances, small developing embryo could not be recovered on all serial section series hindering their presence in some pictures. In a first attempt to integrate ISH and tissue-specific transcriptomic results, the initial selection of genes belonging to the Class III peroxidase multigenic family (*AtPRXs*; Supplementary Table S2) were organized within Fig. 2 and among Supplementary Figs. S4–S27, following decreasing maximum individual tissue-specific transcriptomic expression values. In a first screening with the 16 most highly expressed *AtPRXs*, ISH signals overall confirmed tissue-specific transcriptomic data for 8 *AtPRXs* (*AtPRX42, 12, 50, 51, 03, 36, 46, 17*; Fig. 2a–d,f,g; Supplementary Figs. S4–S7 and S11–S14). For *AtPRX42, 51, 03*, ISH provided new signals not detected by tissue-specific transcriptomics in young embryo and peripheral endosperm tissues (Fig. 2d; Supplementary Figs. S4, S7 and S11). For 3 other *AtPRX*s (*AtPRX55, 22, 23*), ISH was fully complementary to tissue-specific transcriptomics since all ISH signals were not detected by tissue-specific transcriptomic data (Supplementary Figs. S10, S16 and S17). *AtPRX12* and *AtPRX36* highly specific spatiotemporal ISH profiles were fully consistent with tissue-specific transcriptomic data, and interestingly, the resolution reached by ISH allowed to demonstrate the *AtPRX12* expression in all endosperm cells and the restriction of *AtPRX36* expression to the outermost cell layer of the 5-layered teguments used for laser capture microdissection (Fig. 2b,f; Supplementary Figs. S5 and S12). We acknowledged that tissue-specific transcriptomics is more sensitive than ISH in chalazal tissues and mature seed coat (Fig. 2a,e,g,h; Supplementary Figs. S4–S11 and S13–S19). Neither the 6 lowly expressed *AtPRXs* nor the 2 *AtPRXs* without spot ID gave positive ISH signal (Fig. 2h–j; Supplementary Figs. S20–S27). To better understand these detection limits, we integrated ISH and tissue-specific transcriptomic results for the 24 *AtPRXs* studied in a single table (Supplementary Table S4). An overall gradient of ISH-positive genes towards ISH-negative genes followed the ranking of decreasing maximum transcriptomic expression values. However, no clear single cut-off between undetected and detected *AtPRX* gene expression could be highlighted, indicating that the ISH sensitivity was dependent on spatiotemporal parameters.

To decipher how spatiotemporality influences sensitivity, we analysed 15 additional non-peroxidase genes with medium to very-high maximum tissue-specific transcriptomic expression values (Supplementary Table S5). We detected ISH signal in agreement with transcriptomic data for the 14 most expressed genes (Fig. 2k–t; Supplementary Figs. S28–S42; Supplementary Table S5). All together, this illustrated how both techniques were complementary since ISH gave new signals not detected through tissue-specific transcriptomics, mostly illustrated in the younger developmental stages for *AtPRX51*, *AtPRX22*, *Cupin, PAP85, EXT3, SBT1.7, RBOHF, AGP31* and *DUF642*; Fig. 2d,n,o,q; Supplementary Figs. S7, S16, S28, S29, S32, S34, S38, S39 and S41; Supplementary Table S5); whereas tissue-specific transcriptomics was more powerful than ISH for 14 *AtPRXs*, *Cupin, EXT3, LAC15, SBT1.7, DIR12, SCPL20, LTP1, RBOHF, TUB4*, mostly illustrated in chalazal tissues at young stages and in seed coat at mature green stages (Fig. 2a,c,d,k; Supplementary Figs. S4, S6, S7, S9–S11, S14–S21, S28, S32–S38 and S40; Supplementary Table S5). The scarcity of ISH signal for preglobular stage could be explained by its underrepresentation on the tissue-arrays.

To propose final simplified integrated models taking into account the high-throughput tissue-specific transcriptomic and medium-throughput ISH data, we sorted, for each tissue/developmental stage, the 23,933 genes available in Supplementary Table S1 according to several ranges of their individual expression value and plotted the summarized colour-coded ISH results for the 39 genes on the top of the resulting histograms (Fig. 3; Supplementary Figs. S46–S51). This highlighted how ISH signal detection thresholds primarily relied on spatiotemporal parameters and allowed to estimate that the number of genes putatively detectable with ISH ranged from hundreds to putatively the whole genome depending on the tissue/developmental stage considered. For example, from the 23,933 genes present on the array, 55–65% were below the tissue-specific transcriptomic detection limit (value of 45), depending on the tissue/developmental stage (Fig. 3; Supplementary Figs. S46–S51). Interestingly, ISH allowed signal detection for several of those genes in tissues such as the embryo at all developmental stages (*e.g.* 7 out of 30 genes in the embryo at the globular stage) giving a chance of ISH success for the whole genome in these tissues/developmental stages (Fig. 3a,c; Supplementary Figs. S46–S51). On the other hand, ISH sensitivity within the seed coat tissues decreased along the seed development kinetics and therefore tissue-specific transcriptomics became more sensitive (Fig. 3b,d; Supplementary Figs. S46–S51). Increased ISH sensitivity could be theoretically obtained for recalcitrant tissues with branched DNA ISH^44^ but these techniques remain expensive prohibiting their application to medium-throughput.

### Medium-throughput *in situ* hybridization spatial resolution and specificity

Beyond the primary interest of our study which is to demonstrate the complementarity of both datasets, we particularly want to emphasise additional advantages of this medium-throughput ISH protocol not achievable through tissue-specific transcriptomics. The first one is the possibility to extend the study to several serial sections for a given gene. For example, the spatiotemporal specific expression of *AtPRX36* in the outermost cell layer of the 5-layered teguments (Fig. 2f; Supplementary Fig. S12; Supplementary Tables S4 and S5) can be refined with a tomographical study allowing some spatial modelization (Supplementary Fig. S43). The second point concerns the commonly underestimated specificity of ISH. *AtPRX50*/*51, AtPRX22*/*23 and LTP1*/*LTP2*, three pairs of tandem duplicated genes with high identity level (82–92% nucleotide identity all along the sequence; Supplementary Table S8) corresponded to ambiguous tissue-specific transcriptomic spot IDs, but gave different -and thus specific- ISH signals (Fig. 2c,d; Supplementary Figs. S6, S7, S16, S17,S31, S37 and S44; Supplementary Tables S4 and S5). The fact that *AtPRX50*/*51* presented the highest nucleotide identity among the 39 studied genes (92.4%; Supplementary Table S8) contributes demonstrating the ISH specificity for the 37 other genes studied. This also indicates that signal specificity in future ISH studies would have to be demonstrated for the 4,176 genes whose second best hit is above this value, and would be secured for all the other genes (Supplementary Table S7). In this study we used sense riboprobe as traditional negative controls. In most cases, we observed no significant background reinforcing corresponding labelling with antisense probes whenever present (Supplementary Figs. S4–S42). In some circumstances, we observed faint signals with both antisense and sense riboprobes mostly in young embryo epidermis (Supplementary Figs. S11, S13, S14, S21, S26 and S38). We arbitrarily chose to classify these signals as background and to not consider corresponding labelling with antisense probes as specific signals. However, it has to be noticed that some residual *in situ* labelling with sense probes could also have some physiological significance since natural antisense RNA, mostly corresponding to lowly expressed genes, are growingly being discovered, *e.g.* in plant or human tissues^45^,^46^. More dedicated and careful studies would be necessary to fully consider the significance of the observed faint labelling with these few sense probes. Two other ways to further demonstrate the signal specificity are illustrated in the case of *AtPRX36* by testing both the loss of ISH signal in a knockout mutant previously characterized and by applying competitive inhibition between *AtPRX36* labelled antisense probes and *AtPRX36* or *AtPRX72* (82.2% nucleotide identity; Supplementary Table S8) unlabelled antisense probes (Supplementary Fig. S45).

### Biological significance of ISH results

Literature also supports the specificity and validity of ISH results. The ISH expression profiles and previously published localization and/or phenotyping are in good agreement for some of the studied genes. *AtPRX36* specific expression in the outermost cell layer of seed coat [also called mucilage secretory cells (MSCs)^47^] at linear cotyledon stage (Supplementary Figs. S12, S43 and S45) is in good agreement with recently published GFP imaging studies under the control of *AtPRX36* promoter and the corresponding phenotype of delayed mucilage release^33^). *SBT1.7* expression in the MSCs from heart to bending cotyledon stages (Supplementary Fig. S34) can be also related to promoter-reporter gene and immunolabelling profiles in this cell layer and the related phenotype of delayed mucilage release^48^. Expression of *SBT1.7* in additional tissues such as the embryo argues for additional roles to be uncovered for this protein. *DIR12*/*DP1* expression in the MSCs detected by ISH (Supplementary Fig. S35) is correlated to the promoter activity^49^. Contrary to *AtPRX36* and *SBT1.7*, the *DIR12*/*DP1* expression profile does not correspond to an obvious mucilage release phenotype^50^ and is rather to be linked to a secondary metabolism phenotype^51^. *LAC15* expression was detected in the second cell layer underneath MSC (Supplementary Fig. S33) in agreement with *LAC15* promoter activity and a role in oxidative polymerization of flavonoids in this cell layer^52^. The strong ISH signal detected in young developing embryo for *EXT3*, (Supplementary Fig. S32) and not detected by transcriptomics can be directly related to the role of EXT3 in structuring the cell plate during initial cell divisions of the embryo leading to a lethal phenotype in a KO mutant^53^,^54^. For all other genes, ISH results constitute a refined spatiotemporal information that is crucial for future functional study of their role.

## Concluding Remarks

Finally, our study allowed

- To provide a highly detailed ISH protocol explaining the means allowing to reach medium-throughput, to acknowledge its relative sensitivity and to demonstrate its high specificity.
- To confirm numerous expression profiles obtained by the tissue-specific transcriptomic approach validating the ISH protocol.
- To complement the transcriptomic data by studying genes having no probe set IDs, corresponding to ambiguous probe set IDs or by detecting ISH signals not revealed by tissue-specific transcriptomics.
- To draw new expression maps with spatially -and temporally- refined expression profiles that will allow to write new hypotheses for functional studies of the cell wall protein candidate genes.
- To integrate both datasets to estimate spatiotemporally-specific thresholds of tissue-specific transcriptomic signal intensity putatively compatible with the ISH protocol for hundreds to tens-of-thousands genes.

We anticipate that this method should be more widely applied in parallel to any tissue-specific transcriptomic studies, contributing to the functional study of numerous genes along the development of various plant or animal models. However, this method is not restricted to model organisms with available transcriptomic facilities since the complementarity of RNAseq large datasets with ISH approach have been recently illustrated for dozens of genes involved in the anticancer alkaloid biosynthetic pathway from the Madagascar periwinkle non model plant^55^,^56^,^57^.

## Additional Information

**How to cite this article**: Francoz, E. *et al.* Complementarity of medium-throughput *in situ* RNA hybridization and tissue-specific transcriptomics: case study of Arabidopsis seed development kinetics. *Sci. Rep.* **6**, 24644; doi: 10.1038/srep24644 (2016).

## Supplementary Material

Supplementary Information

Supplementary Table S1

Supplementary Tables S2-S5

Supplementary Tables S6-S8
