# Supplementary material for: Complementarity of medium-throughput in situ RNA hybridization and tissue-specific transcriptomics: case study of Arabidopsis seed development kinetics
Source: Sci Rep. 2016 Apr 20;6:24644. doi: 10.1038/srep24644 (PMC4837347; doi:10.1038/srep24644)
Supplement: Supplementary Information [file srep24644-s1.pdf]

Scientific Reports

Complementarity of medium-throughput *in situ* RNA hybridization and tissue-specific transcriptomics: case study of Arabidopsis seed development kinetics

Edith Francoz[1], Philippe Ranocha[1], Clémentine Pernot[1], Aurélie Le Ru[2], Valérie Pacquit[1], Christophe Dunand[1] and Vincent Burlat[1]

[1] Laboratoire de Recherche en Sciences Végétales, Université de Toulouse, CNRS, UPS, 24 chemin de Borde Rouge, Auzeville, BP42617, 31326 Castanet Tolosan, France
[2] Fédération de Recherche 3450, Plateforme Imagerie, Pôle de Biotechnologie Végétale, Castanet-Tolosan 31326, France.

SUPPLEMENTARY INFORMATION:

Supplementary Figure S1: Comparison of transcriptomic tissue coverage along seed developmental stages and of the density of samples gathered within tissue arrays used for *in situ* hybridization.

Supplementary Figure S2: Map of multiple cloning sites (MCSs) from RAFL2-25 vectors used in the RIKEN bioresource center and from the pGEM-T Easy vector.

Supplementary Figure S3: Restriction maps (5' overhang and blunt enzymes) of the four RIKEN vector series.

Supplementary Figure S4 to S42: Comparison of tissue-specific transcriptomic expression maps with the *in situ* hybridization (ISH) results and the new ISH cell-specific expression map for 39 genes.

Supplementary Figure S43: Illustration of the possibility to use serial sections for a same riboprobe pair to draw tomographical view of *in situ* hybridization (ISH) signals.

Supplementary Figure S44: Illustration of the specificity of *in situ* hybridization (ISH) signals for two duplicated genes (LTP1 and LTP2) presenting 83.2% nucleotide sequence identity.

Supplementary Figure S45: Illustration of the specificity of *in situ* hybridization (ISH) signals using competitive inhibition and knock out (KO) mutant line.

Supplementary Figures S46 - S51: Thresholds of tissue-specific transcriptomic values compatible with ISH are specific to each developmental stage/tissue.

Supplementary Methods: Detailed step-by-step protocol and tips.

Supplementary Table S9: List of plasmids used in this study and conditions for riboprobe *in vitro* transcription.

Supplementary Table S10: Primers and PCR information for the cloning in pGEM□T Easy of RBOHE, EXT3, LTP1, AtPRX13, AtPRX43 and AtPRX46.

Supplementary Tables S1-S8 are available as separate Microsoft Excel files

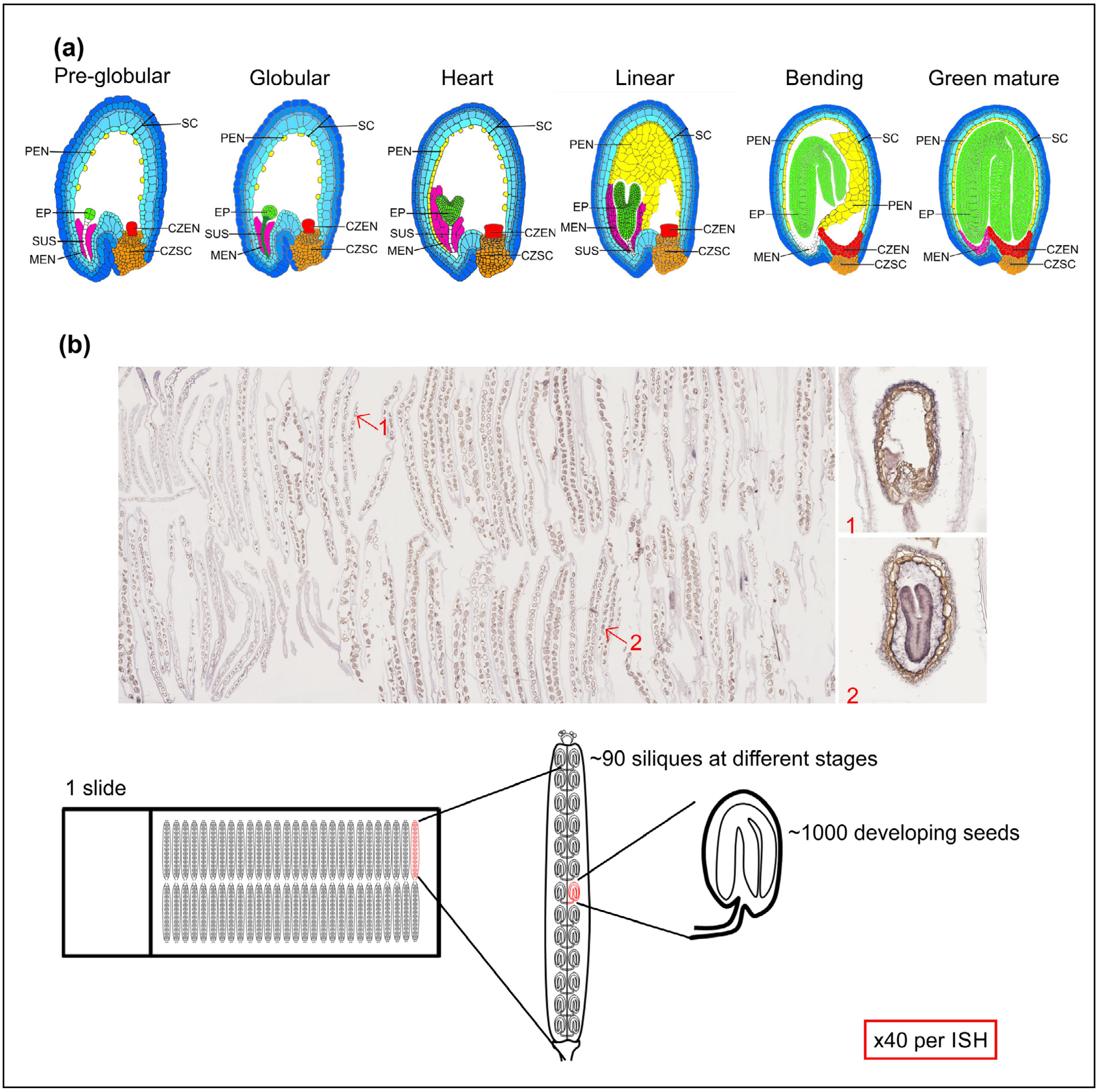

Supplementary Figure S1: Comparison of transcriptomic tissue coverage along seed developmental stages and of the density of samples gathered within tissue arrays used for *in situ* hybridization. (a) The cartoons displaying the 6 stages of seed development used for the tissue-specific transcriptomic studies by Belmonte *et al.* (2013) are re-coloured after original drawings from Seedgenenetwork at http://estdb.biology.ucla.edu/ seed/ with each colour corresponding to one tissue sampled by laser capture microdissection (42 samples in total). Note that the 5 cell layers of seed coat coloured in various shades of blue correspond to a single seed coat sample for each developmental stage. **(b)** Example of one ISH slide following scanning at high resolution (full slide area view and 2 magnified zones at different developmental stages), and drawing illustrating the density of samples on one slide.

Abbreviations: CZEN, chalazal endosperm; CZSC, chalazal seed coat; EP, embryo proper; MEN, micropylar endosperm; PEN, peripheral endosperm; SC, seed coat; SUS, suspensor.

RAFL02-RAFL03 map

```
2821    CGCCAGGGTT TTCCCAGTCA CGACGTTGTA AAACGACGGC CAGTGAATTG TAATACGACT
                                                         T7 promotor⇨
2881    CACTATAGGG CGAATTGGGT ACCGGGCCCC CCCTCGATTG GCCAAATCGG CCCTCGAGTT
                         Asp718                                   XhoI
2941    TTTTTTTTTT TTTT←------------------------------------------------
                                       3'cDNA5'
1       GGGGGGGGGG GGGGACTAGT GAGCTCGGCC ATAAGGGCCA TAGCTCCAGC TTTTGTTCCC
                       SpeI  EcoICRI
61      TTTAGTGAGG GTTAATTTCG AGCTTGGCGT AATCATGGTC ATAGCTGTTT CCTGTGTGAA
          ⇦T3 promotor
```

RAFL04-RAFL06 map

```
2821    AGGGTTTTCC CAGTCACGAC GTTGTAAAAC GACGGCCAGT GAATTGTAAT ACGACTCACT
                                                         T7 promotor⇨
2881    ATAGGGCGAA TTGGGTACCG GGCCCCCCCT CGATTGGCCA AATCGGCCCT CGAGTTAATT
                    Asp718                                    XhoI
2941    AAATTAATCC CCCCCCCCC-------------------------------------------→
                                       5'cDNA3'
1       AAAAAAAAAA AAAAAAGAGC TCGGCCATAA GGGCCATAGC TCCAGCTTTT GTTCCCTTTA
                        EcoICRI
61      GTGAGGGTTA ATTTCGAGCT TGGCGTAATC ATGGTCATAG CTGTTTCCTG TGTGAAATTG
        ⇦T3 promotor
```

RAFL07-11,RAFL26 map

```
2881    GCCAGTGAAT TGTAATACGA CTCACTATAG GGCGAATTGG AGCTCCACCG CGGTGGCGGC
                          T7 promotor⇨                               NotI
2941    CGCATAACTT CGTATAGGAT ACATTATACG AAGTTATGGA TCAGGCCAAA TCGGCCGAGC

3001    TCGAATTCGT CGAGTTAATT AAATTAATCC CCCCCCCCC---------------------→
          EcoRI                                          5'cDNA3'
1       AAAAAAAAAA AAAAAAGAGC TCTTGGATCC GGCCATAAGG GCCTGATCCT TCGAGGGGGG
                       EcoICRI   BamHI
61      GCCCGGTACC AGCTTTTGTT CCCTTTAGTG AGGGTTAATT TCGAGCTTGG CGTAATCATG
            Asp718                 ⇦T3 promotor
```

RAFL12-RAFL25 map

```
2881    GACGGCCAGT GAATTGTAAT ACGACTCACT ATAGGGCGAA TTGGAGCTCC ACCGCGGTGG
                                T7 promotor⇨
2941    CGGCCGCATA ACTTCGTATA GCATACATTA TACGAAGTTA TGGATCAGGC CAAATCGGCC
          NotI
3001    GAGCTCGAAT TCGTCGAGAA CCG------------------------------------→
              EcoRI                           5'cDNA3'
1       AAAAAAAAAA AAAAAACTAT CCAGGGTTGG ATCCGGCCAT AAGGGCCTGA TCCTTCGAGG
                                      BamHI
61      GGGGGCCCGG TACCAGCTTT TGTTCCCTTT AGTGAGGGTT AATTTCGAGC TTGGCGTAAT
                 Asp718                ⇦T3 promotor
```

pGEMT-easy map

```
5'. TGTAA TACGA CTCAC TATAG GGCGA ATTGG GCCCG ACGTC GCATG CTCCC GGCCG CCATG
            T7 promotor⇨                                                NcoI
    GCGGC CGCGG GAATT CGATT←--------------------------→A ATCAC TAGTG AATTC
                         5'cDNA3' or 3'cDNA5'               SpeI
    GCGGC CGCCT GCAGG TCGAC CATAT GGGA GAGCT CCCAA CGCGT  TGGAT GCATA GCTTG
                      SalI    NdeI
    AGTAT TCTAT AGTGT CACCT AAAT .  3'
          ⇦SP6 promotor
```

Supplementary Figure S2: Map of multiple cloning sites (MCSs) from RAFL2-25 vectors used in the RIKEN bioresource center and from the pGEM-T Easy vector. The cDNA orientation and RNA polymerase promoter sequences are displayed. The single-cut restriction sites that can be used for plasmid linearization are colour-coded in green (5' overhang) and yellow (blunt).

Non Cut Enzymes **5' overhang (RAFL02-RAFL03)**					
AccI	AccIII	AflII	AgeI	AscI	AsuII
AvrII	BamHI	BbvII	BclI	BglII	Bpu1102I
Bsc91I	Bsp1407I	BspMI	BspMII	BssHII	BstEII
Bsu36I	ClaI	Csp45I	CspI	CvnI	EagI
Eco52I	EcoNI	EcoRI	EspI	HindIII	MfeI
Mlu113I	MluI	MstII	NarI	NcoI	NdeI
NheI	NotI	PinAI	PpuMI	SalI	SauI
SgrAI	SplI	StyI	SunI	Tth111I	XbaI
XmaI	XmaIII				
Non Cut Enzymes **blunt (RAFL02-RAFL03)**					
Eco47III	Eco72I	EcoRV	EheI	HindII	HpaI
NruI	PmaCI	PmeI	SmaI	SnaBI	SpoI
SrfI	StuI	SwaI			

Non Cut Enzymes **5' overhang (RAFL04-RAFL06)**					
AccI	AccIII	AflII	AgeI	AscI	AsuII
AvrII	BamHI	BbvII	BclI	BglII	Bpu1102I
Bsc91I	Bsp1407I	BspMI	BspMII	BssHII	BstEII
Bsu36I	ClaI	Csp45I	CspI	CvnI	EagI
Eco52I	EcoNI	EcoRI	EspI	HindIII	MfeI
Mlu113I	MluI	MstII	NarI	NcoI	NdeI
NheI	NotI	PinAI	PpuMI	SalI	SauI
SgrAI	SpeI	SplI	StyI	SunI	Tth111I
XbaI	XmaI	XmaIII			
Non Cut Enzymes **blunt (RAFL04-RAFL06)**					
Eco47III	Eco72I	EcoRV	EheI	HindII	HpaI
NruI	PmaCI	PmeI	SmaI	SnaBI	SpoI
SrfI	StuI	SwaI			

Non Cut Enzymes **5' overhang (RAFL07-11,RAFL26)**					
AccI	AccIII	AflII	AgeI	AscI	AsuII
AvaI	AvrII	BbvII	BclI	BglII	Bpu1102I
Bsc91I	Bsp1407I	BspMI	BspMII	BssHII	BstEII
Bsu36I	ClaI	Csp45I	CspI	CvnI	EcoNI
EspI	HindIII	MfeI	MluI	MstII	NarI
NcoI	NdeI	NheI	PinAI	PpuMI	SalI
SauI	SgrAI	SpeI	SplI	StyI	SunI
Tth111I	XbaI	XhoI	XmaI		
Non Cut Enzymes **blunt (RAFL07-11,RAFL26)**					
BalI	Eco47III	Eco72I	EcoRV	EheI	HindII
HpaI	MscI	NruI	PmaCI	PmeI	SciI
SmaI	SnaBI	SpoI	SrfI	StuI	SwaI

Non Cut Enzymes **5' overhang (RAFL12-RAFL25)**					
AccI	AccIII	AflII	AgeI	AscI	AsuII
AvaI	AvrII	BbvII	BclI	BglII	Bpu1102I
Bsc91I	Bsp1407I	BspMI	BspMII	BssHII	BstEII
Bsu36I	ClaI	Csp45I	CspI	CvnI	EcoNI
EspI	HindIII	MfeI	MluI	MstII	NarI
NcoI	NdeI	NheI	PinAI	PpuMI	SalI
SauI	SgrAI	SpeI	SplI	StyI	SunI
Tth111I	XbaI	XhoI	XmaI		
Non Cut Enzymes **blunt (RAFL12-RAFL25)**					
BalI	Eco47III	Eco72I	EcoRV	EheI	HindII
HpaI	MscI	NruI	PmaCI	PmeI	SciI
SmaI	SnaBI	SpoI	SrfI	StuI	SwaI

Supplementary Figure S3: Restriction maps (5' overhang and blunt enzymes) of the four RIKEN vector series. These restriction maps allow, by comparison with the restriction map of each clones, to select enzymes that cut once at cDNA extremities and not in the vector in case where restriction sites present in the MCS of the vector cannot be used for the linearization since they also cut in the cDNA. Selection of classical enzymes are highlighted in green (5' overhang) and yellow (blunt).

Supplementary Figure S4 to S42: Comparison of tissue-specific microtranscriptomic expression maps with the *in situ* hybridization (ISH) results and the new ISH cell-specific expression map for 39 genes.

Each of the following 39 **Supplementary Figures** allows comparisons for five developmental stages between: **(a)** a screen copy of the seed eFP browser tissue-specific microtranscriptomic map including the individual absolute heatmap scale (red-to-yellow colour codes correspond to high-to-low expression values) that is different for each gene (Winter *et al.,* 2007),

(b) the corresponding ISH results for both the antisense (AS) and sense (S) probe used as a negative control, and **(c)** the final corresponding new cell-specific ISH map re-coloured after original drawings from Belmonte *et al*. (2013) available at Seedgenenetwork (http://estdb.biology.ucla.edu/ seed/) giving increased cellular resolution and using a unique colour code for all genes with red corresponding to strong signals, orange corresponding to moderate signals and white corresponding to the absence of detected ISH signal. The 24 *AtPRX* genes appear in **Supplementary Figures S4 to S27** and the 15 additional non-peroxidase genes appear in **Supplementary Figures S28 to S42** following the decreasing order of their maximum microtranscriptomic values. Scale bars, 100µm.

Supplementary Figure S4: *AtPRX42 (AT4G21960)*
Supplementary Figure S5: *AtPRX12 (AT1G71695)*
Supplementary Figure S6: *AtPRX50 (AT4G37520)*
Supplementary Figure S7: *AtPRX51 (AT4G37530)*
Supplementary Figure S8: *AtPRX43 (AT4G25980)*
Supplementary Figure S9: *AtPRX56 (AT5G15180)*
Supplementary Figure S10: *AtPRX55 (AT5G14130)*
Supplementary Figure S11: *AtPRX03 (AT1G05260)*
Supplementary Figure S12: *AtPRX36 (AT3G50990)*
Supplementary Figure S13: *AtPRX46 (AT4G31760)*
Supplementary Figure S14: *AtPRX17 (AT2G22420)*
Supplementary Figure S15: *AtPRX11 (AT1G68850)*
Supplementary Figure S16: *AtPRX22 (AT2G38380)*
Supplementary Figure S17: *AtPRX23 (AT2G38390)*
Supplementary Figure S18: *AtPRX21 (AT2G37130)*
Supplementary Figure S19: *AtPRX64 (AT5G42180)*
Supplementary Figure S20: *AtPRX66 (AT5G51890)*
Supplementary Figure S21: *AtPRX53 (AT5G06720)*
Supplementary Figure S22: *AtPRX69 (AT5G64100)*
Supplementary Figure S23: *AtPRX71 (AT5G64120)*
Supplementary Figure S24: *AtPRX62 (AT5G39580)*
Supplementary Figure S25: *AtPRX72 (AT5G66390)*
Supplementary Figure S26: *AtPRX13 (AT1G77100)*
Supplementary Figure S27: *AtPRX32 (AT3G32980)*
Supplementary Figure S28: *Cupin (AT4G36700)*
Supplementary Figure S29: *PAP85 (AT3G22640)*
Supplementary Figure S30: *GRP (AT2G05580)*
Supplementary Figure S31: *LTP2 (AT2G38530)*
Supplementary Figure S32: *EXT3 (AT1G21310)*
Supplementary Figure S33: *LAC15 (AT5G48100)*
Supplementary Figure S34: *SBT1.7 (AT5G67360)*
Supplementary Figure S35: *DIR12 (AT4G11180)*
Supplementary Figure S36: *SCPL20 (AT4G12910)*
Supplementary Figure S37: *LTP1 (AT2G38540)*
Supplementary Figure S38: *RBOHF (AT1G64060)*
Supplementary Figure S39: *AGP31 (AT1G28290)*
Supplementary Figure S40: *TUB4 (AT5G44340)*
Supplementary Figure S41: *DUF642 (AT3G08030)*
Supplementary Figure S42: *RBOHE (AT1G19230)*

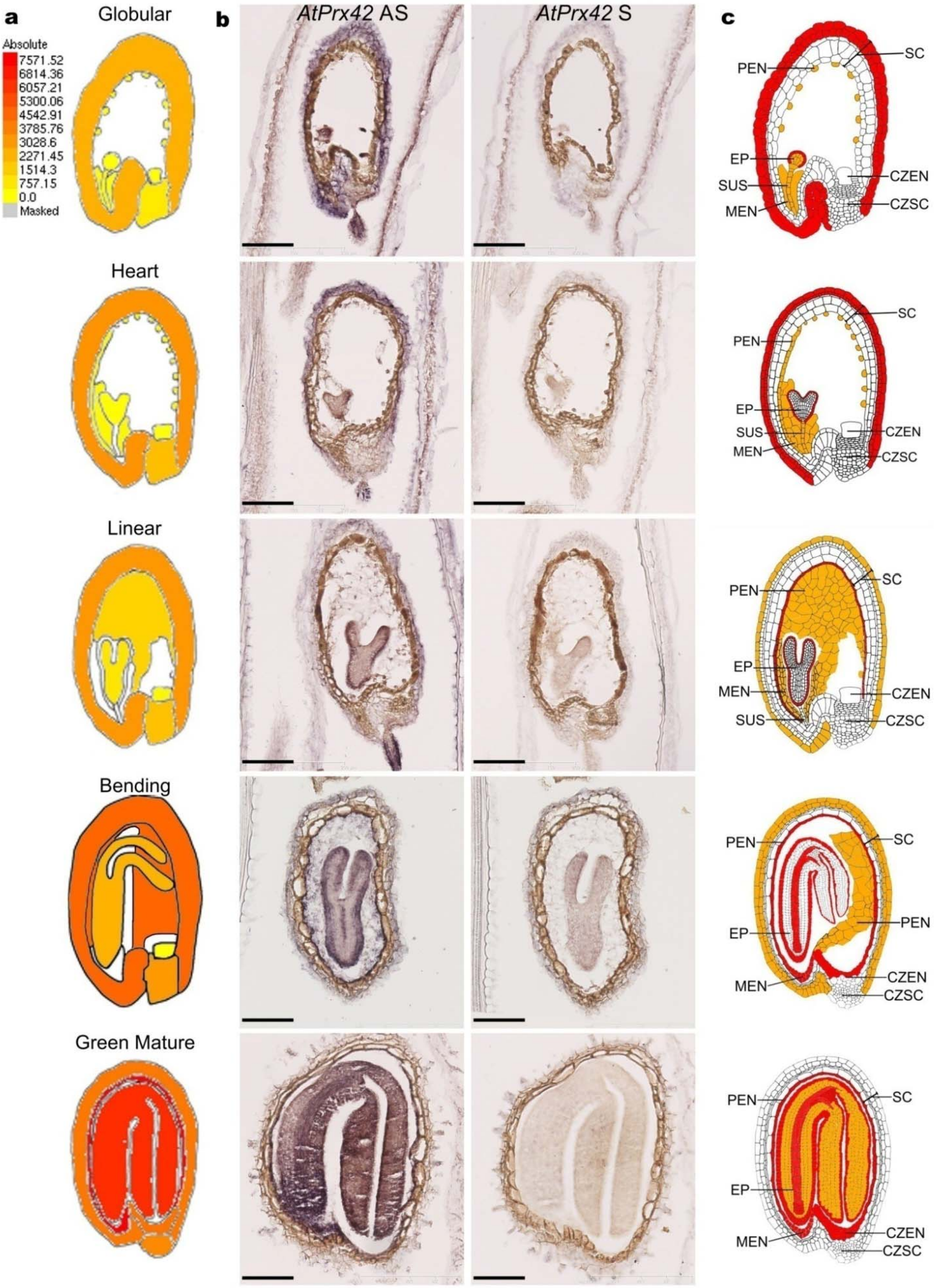

Supplementary Figure S4: *AtPRX42* *(AT4G21960)*

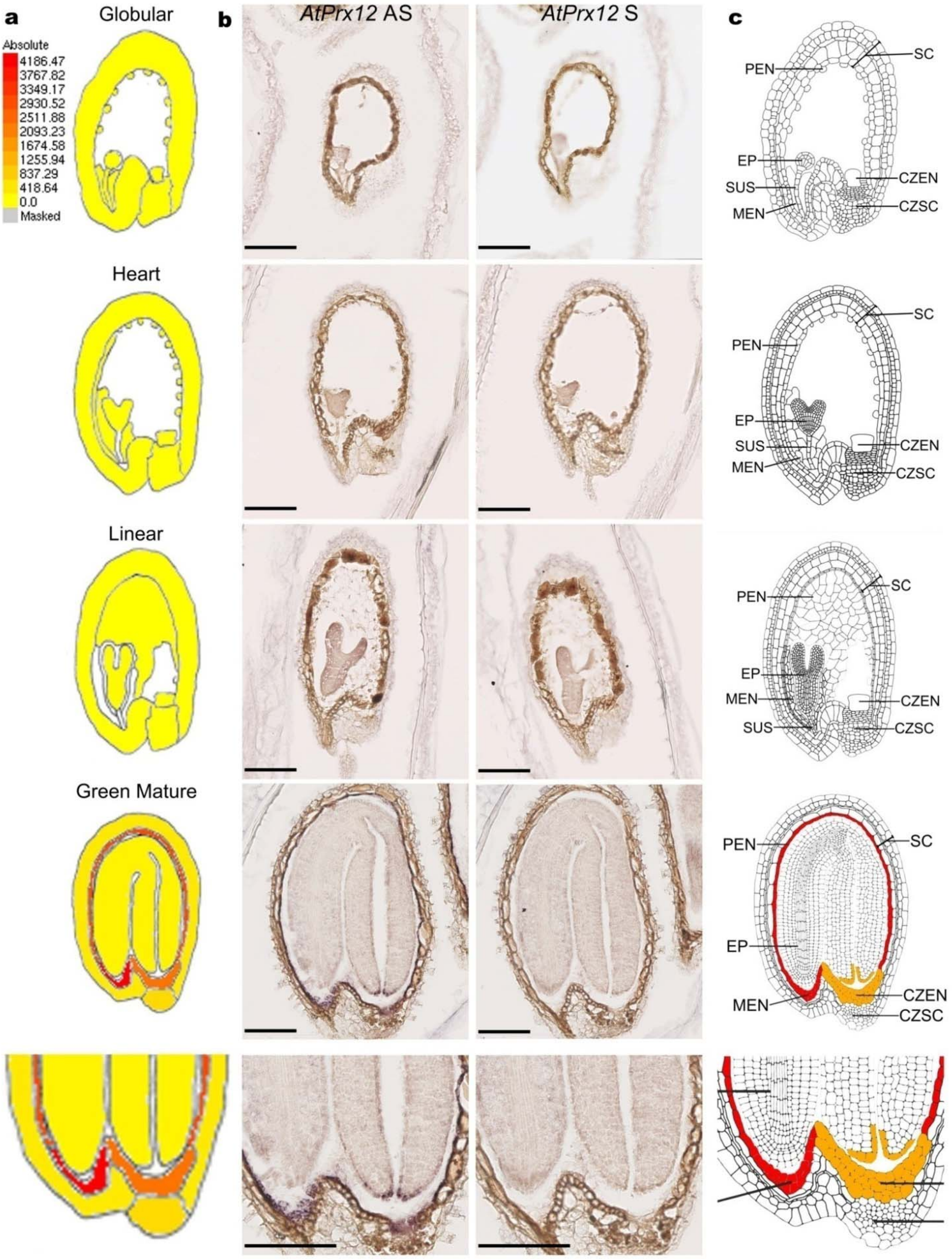

Supplementary Figure S5: *AtPRX12 (AT1G71695)*

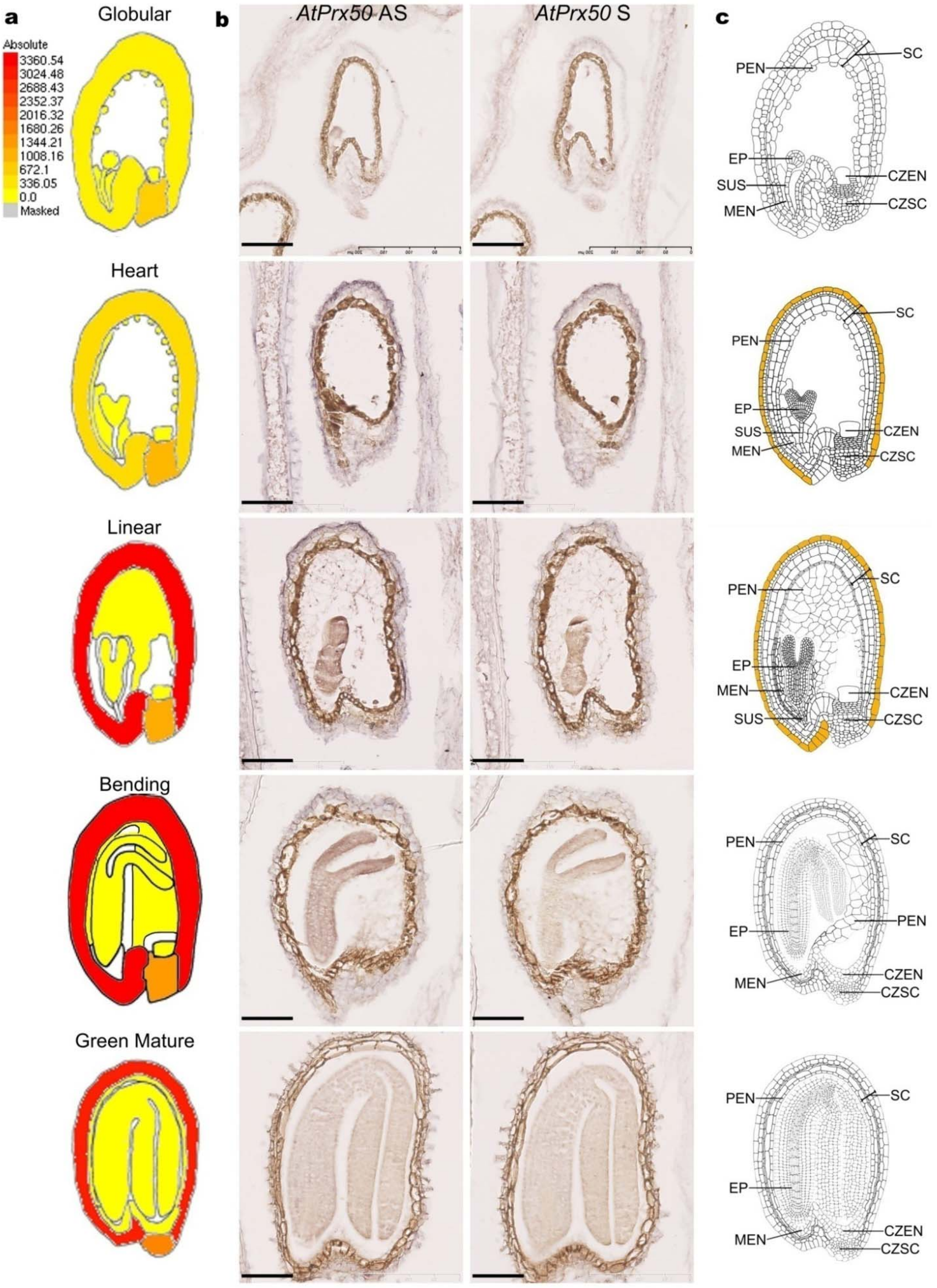

Supplementary Figure S6: *AtPRX50* *(AT4G37520)*

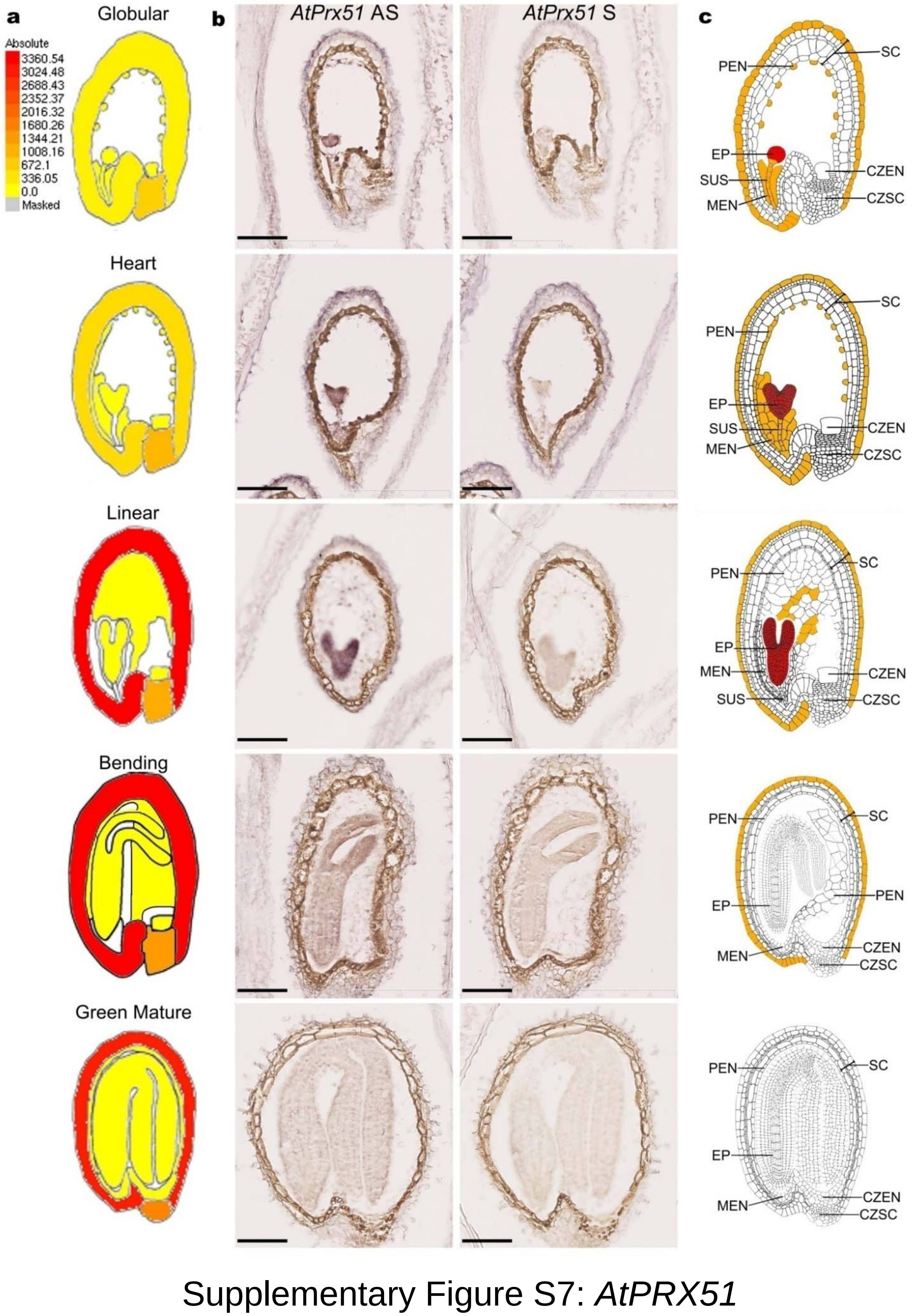

Supplementary Figure S7: *AtPRX51* *(AT4G37530)*

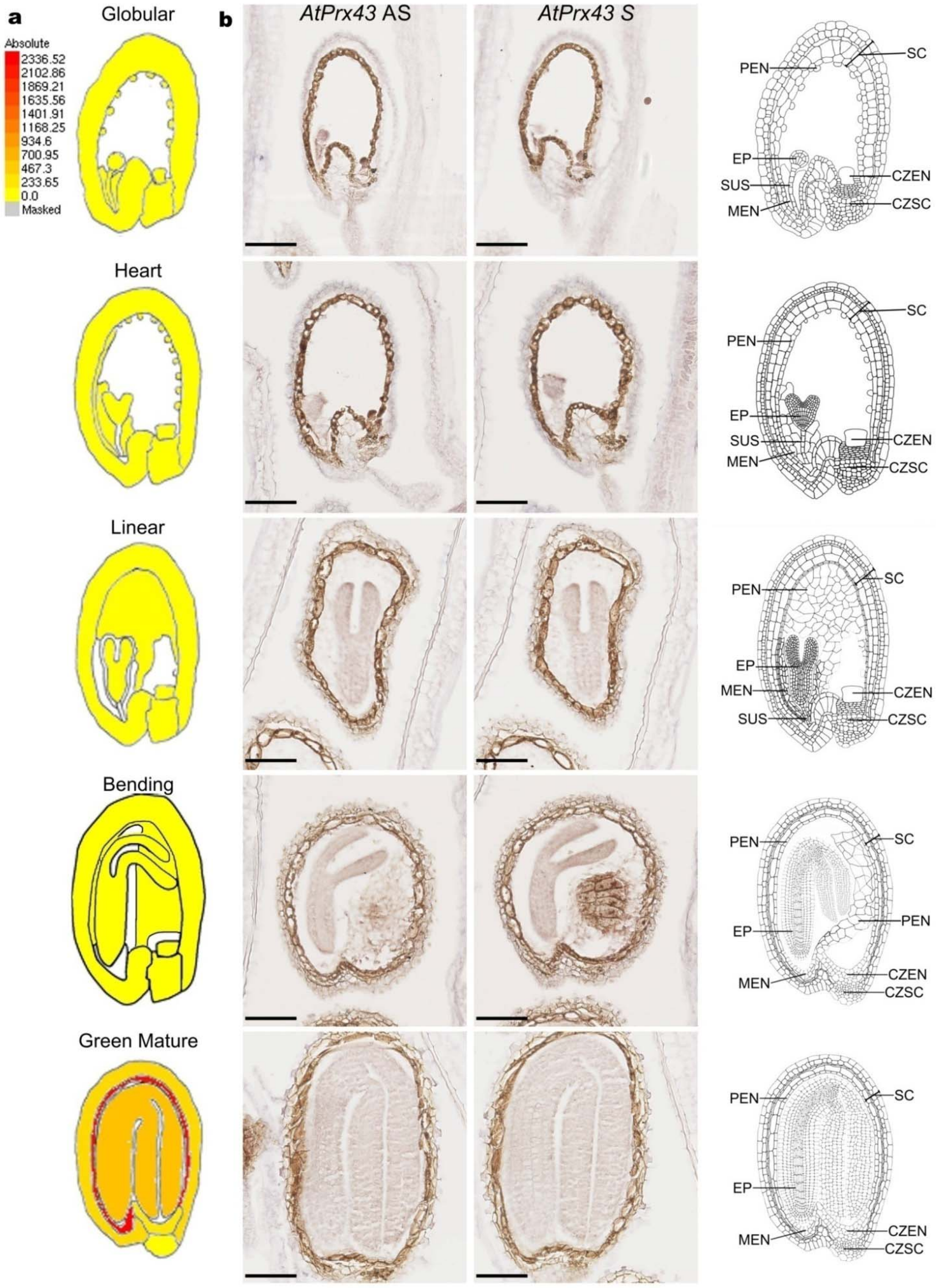

Supplementary Figure S8: *AtPRX43 (AT4G25980)*

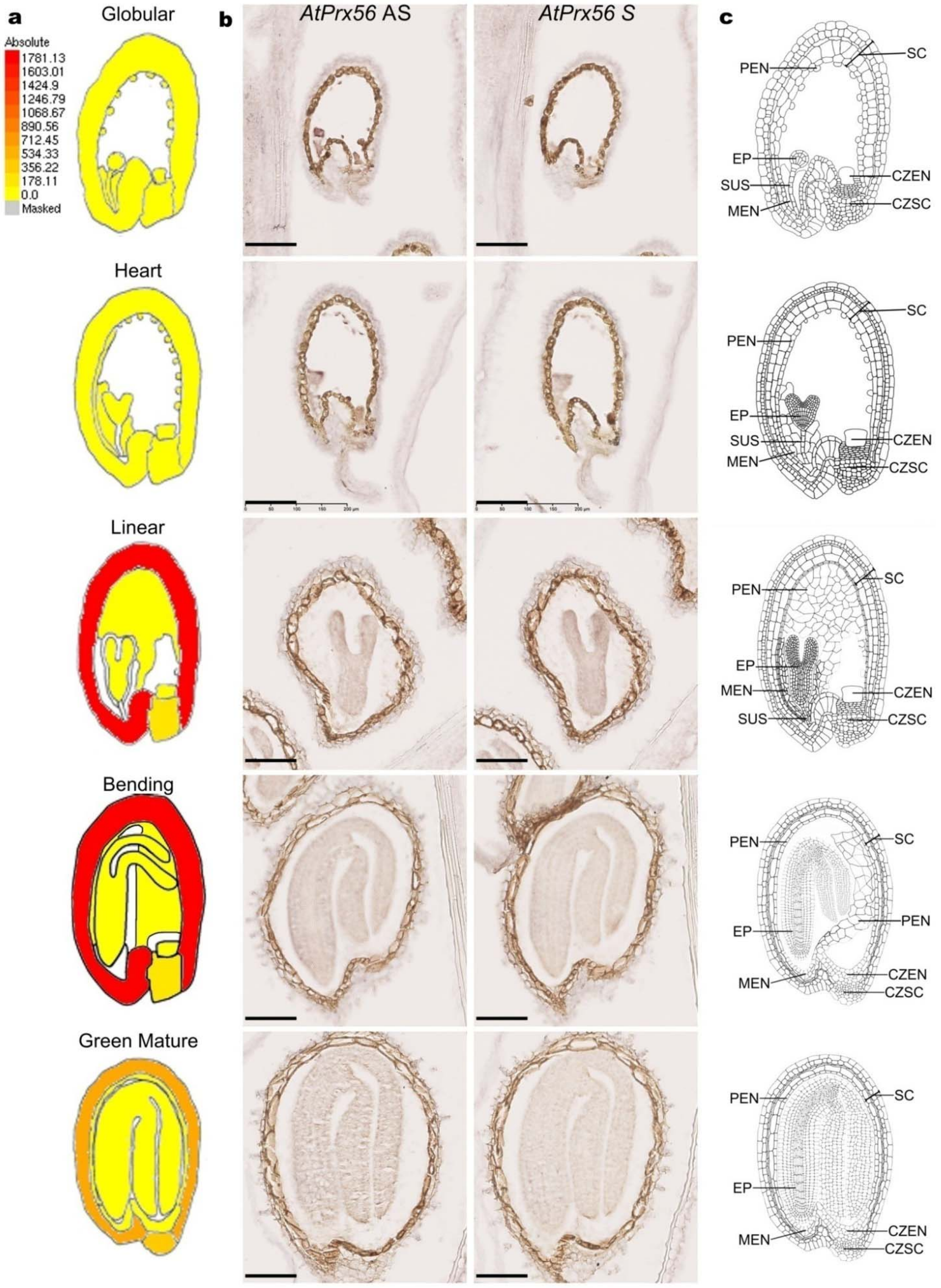

Supplementary Figure S9: *AtPRX56 (AT5G15180)*

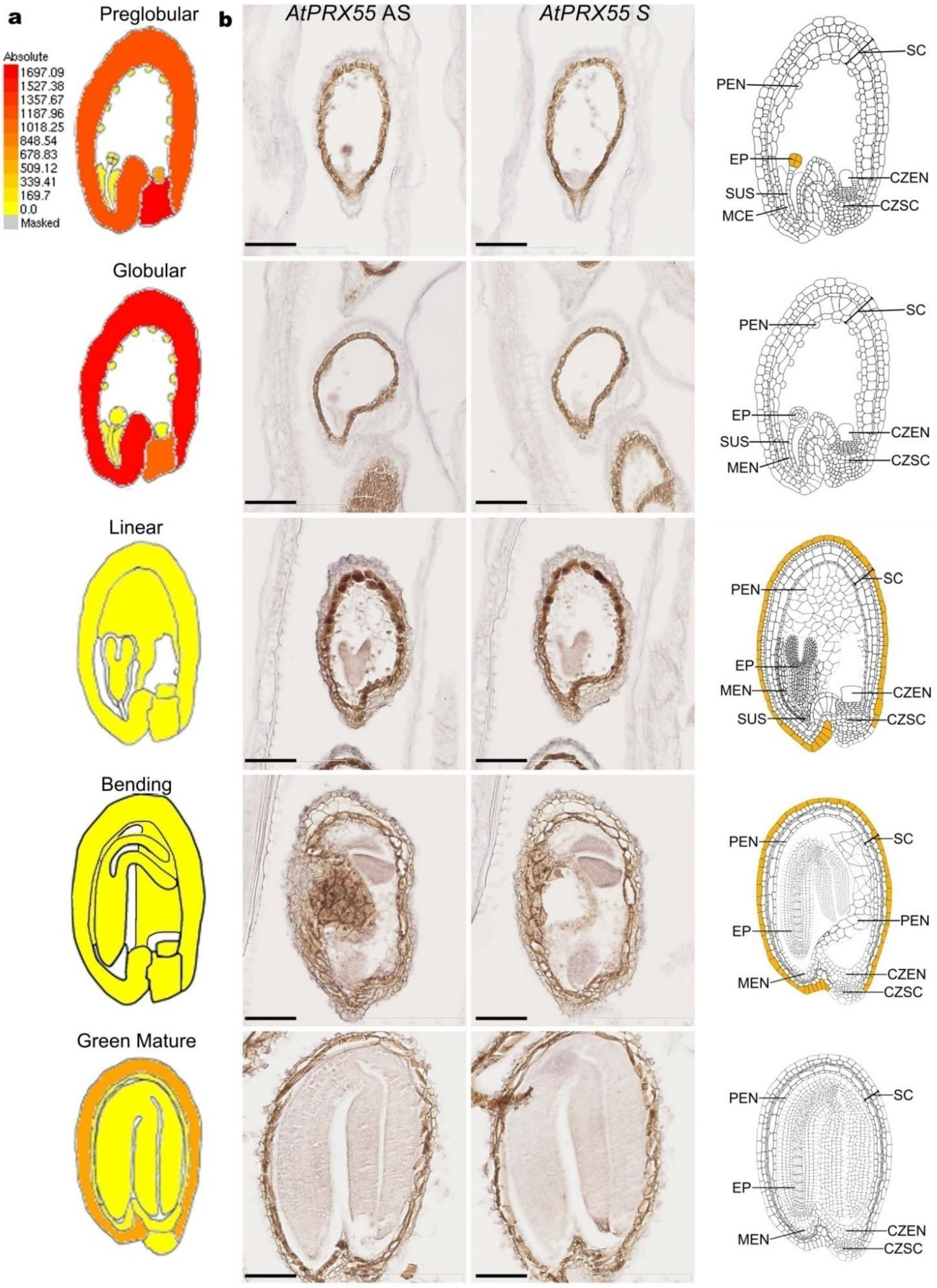

Supplementary Figure S10: *AtPRX55* *(AT5G14130)*

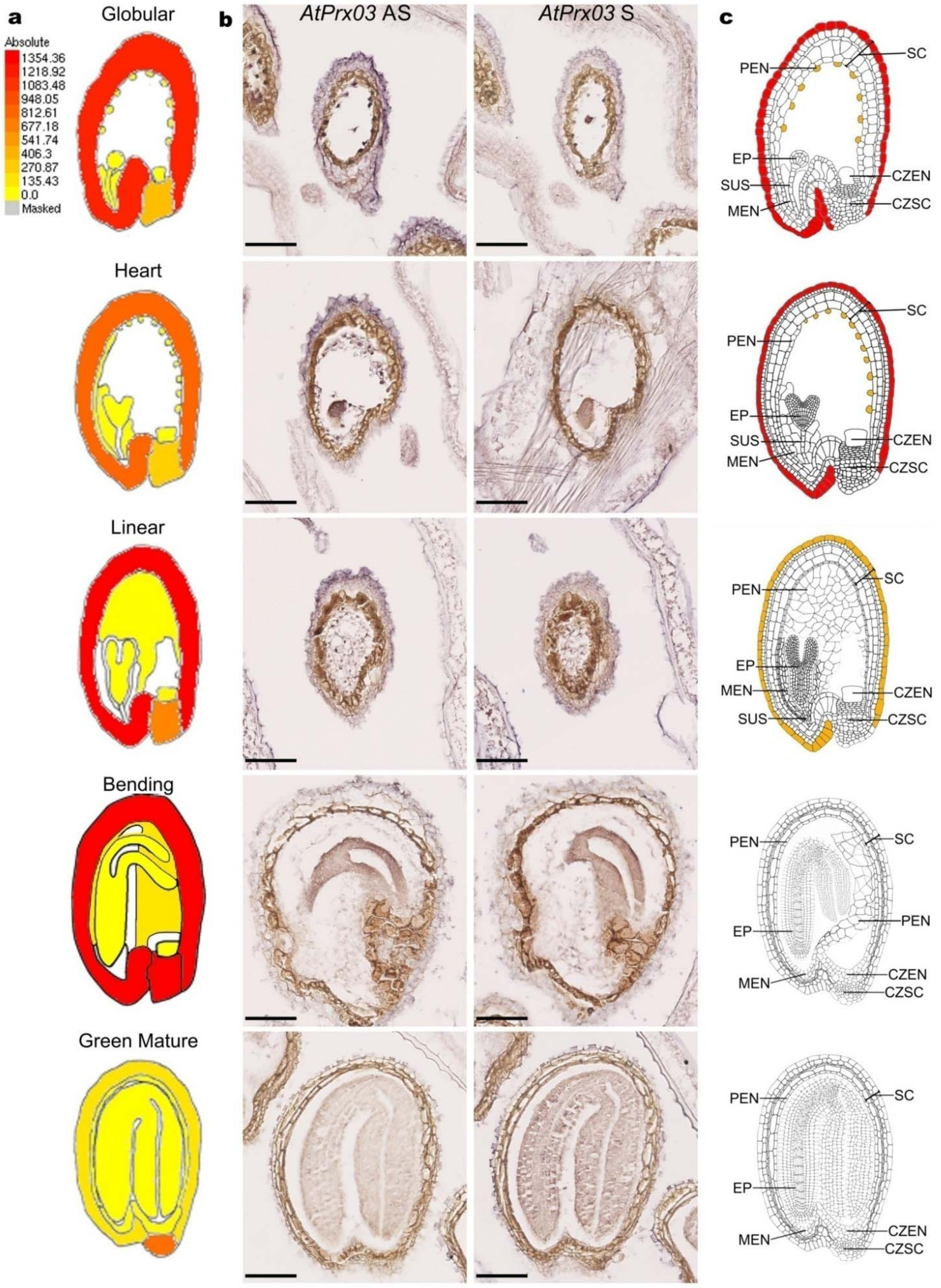

Supplementary Figure S11: *AtPRX03 (AT1G05260)*

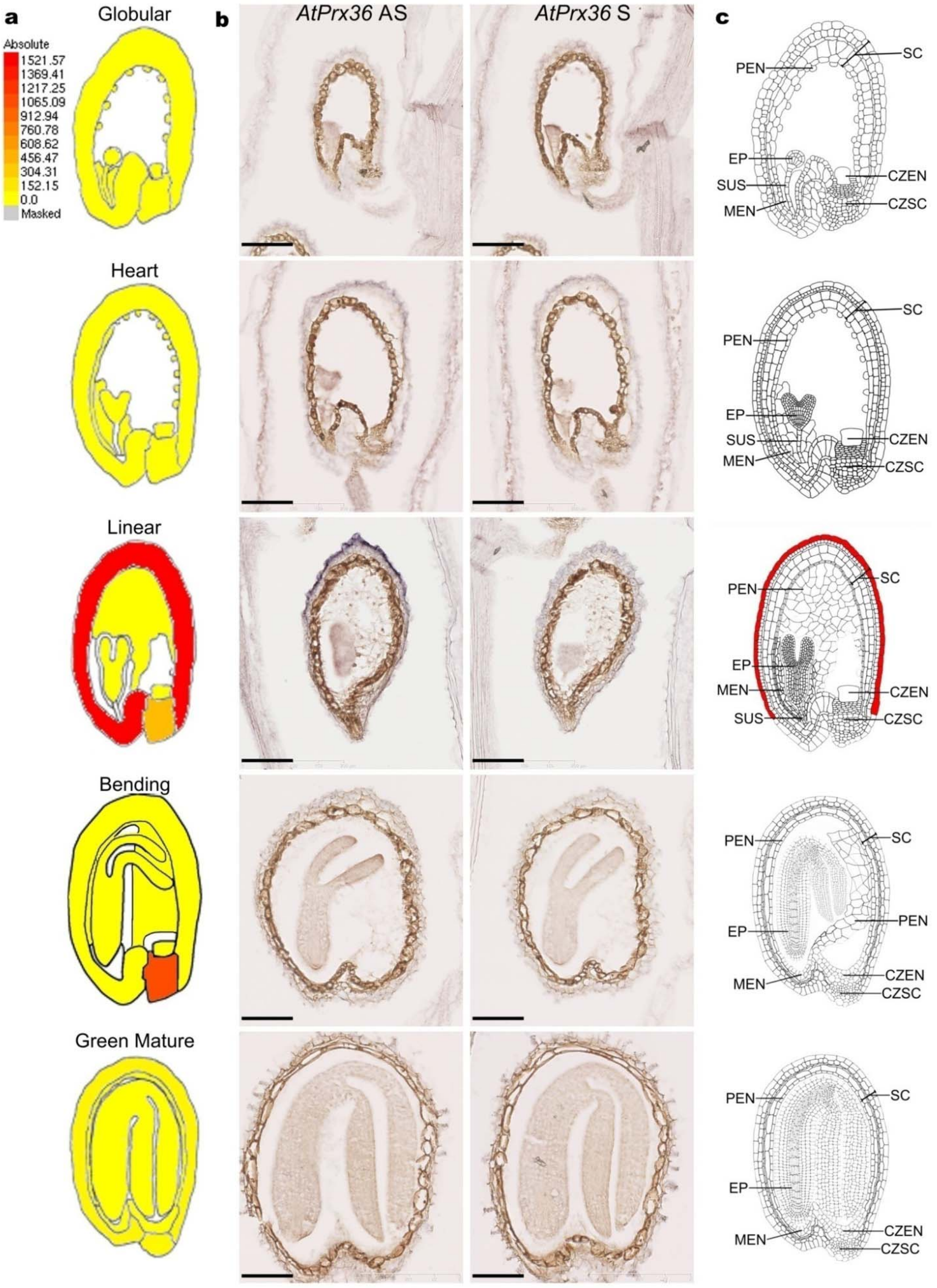

Supplementary Figure S12: *AtPRX36 (AT3G50990)*

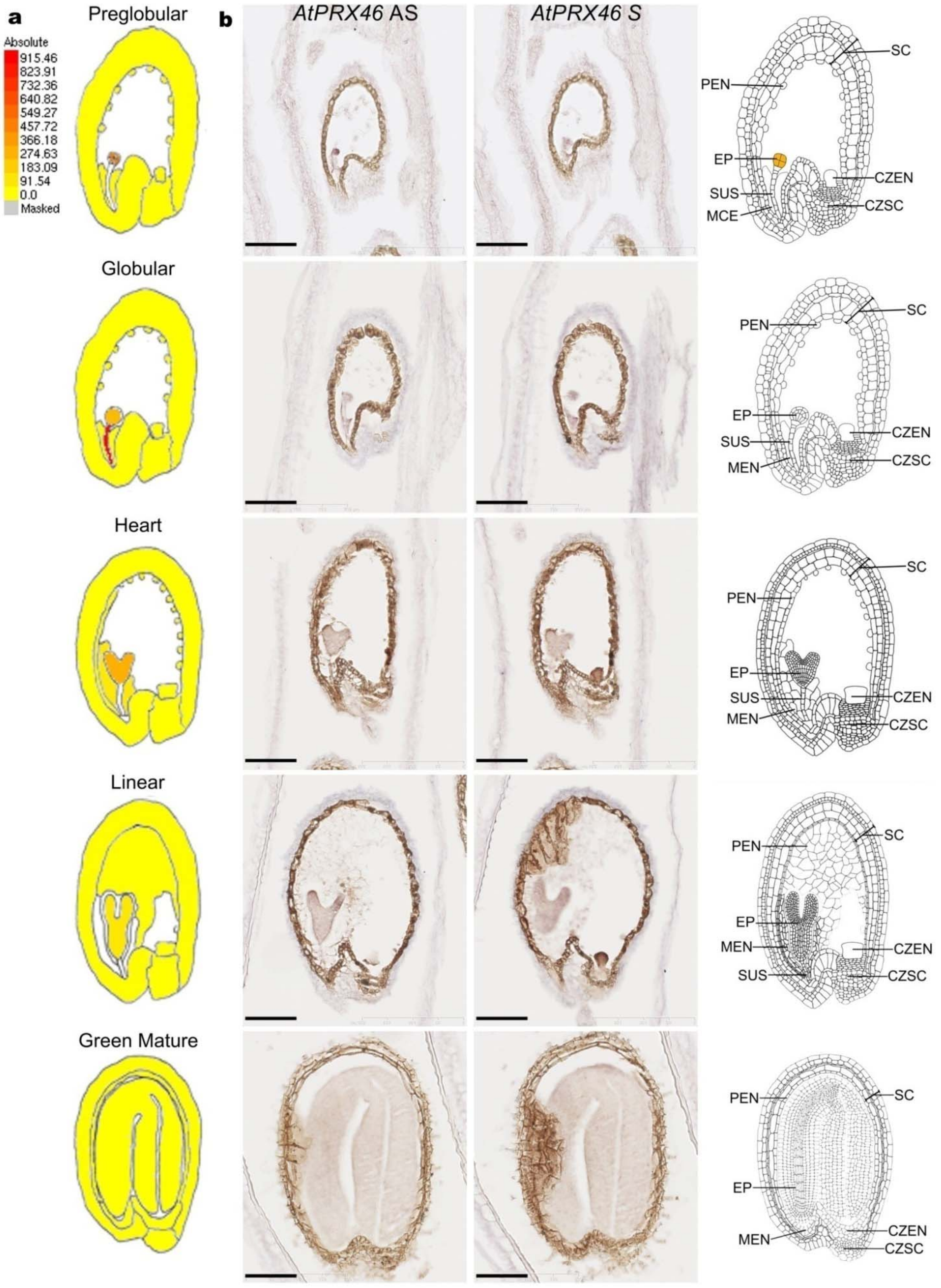

Supplementary Figure S13: *AtPRX46* *(AT4G31760)*

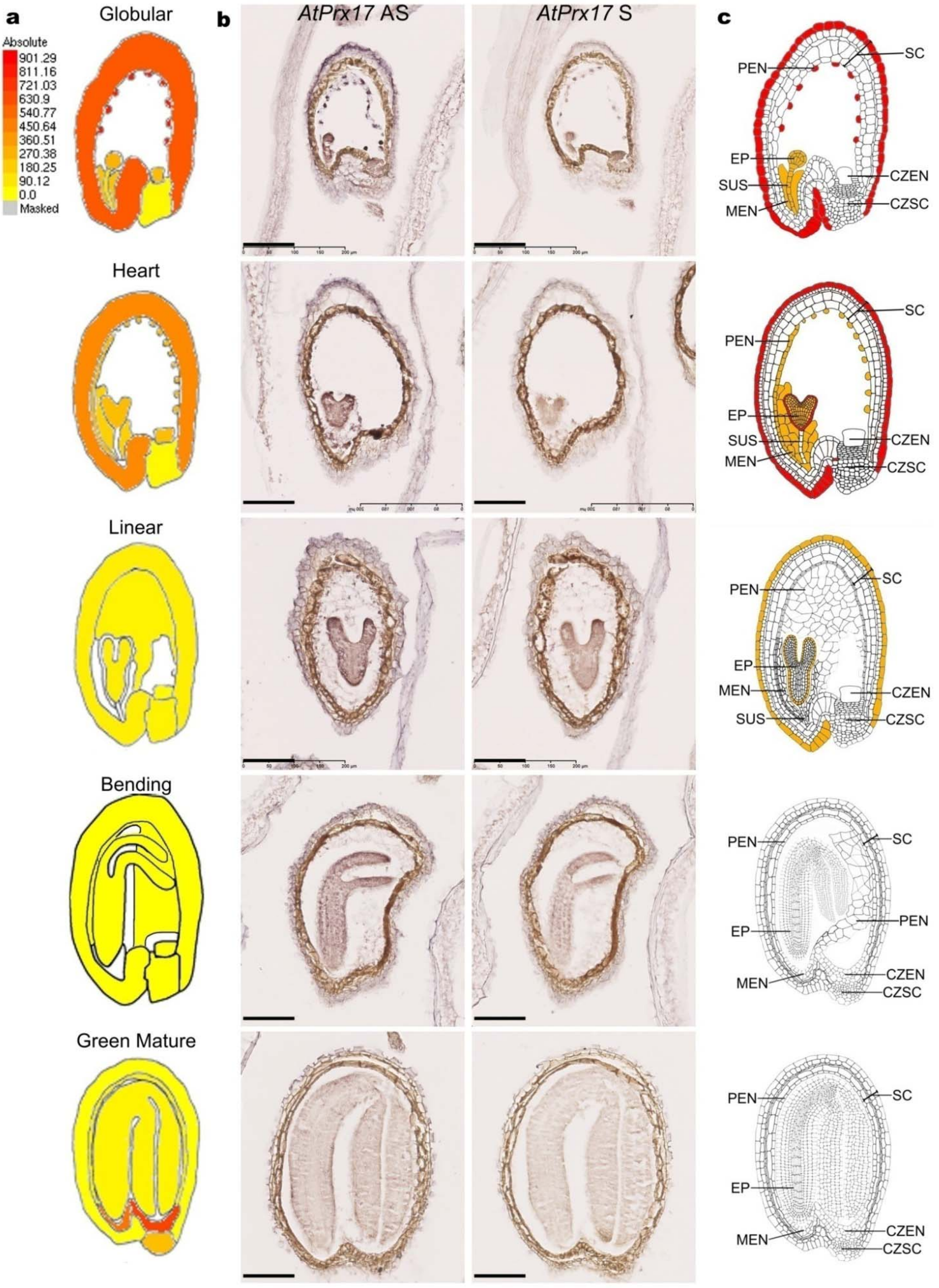

Supplementary Figure S14: *AtPRX17 (AT2G22420)*

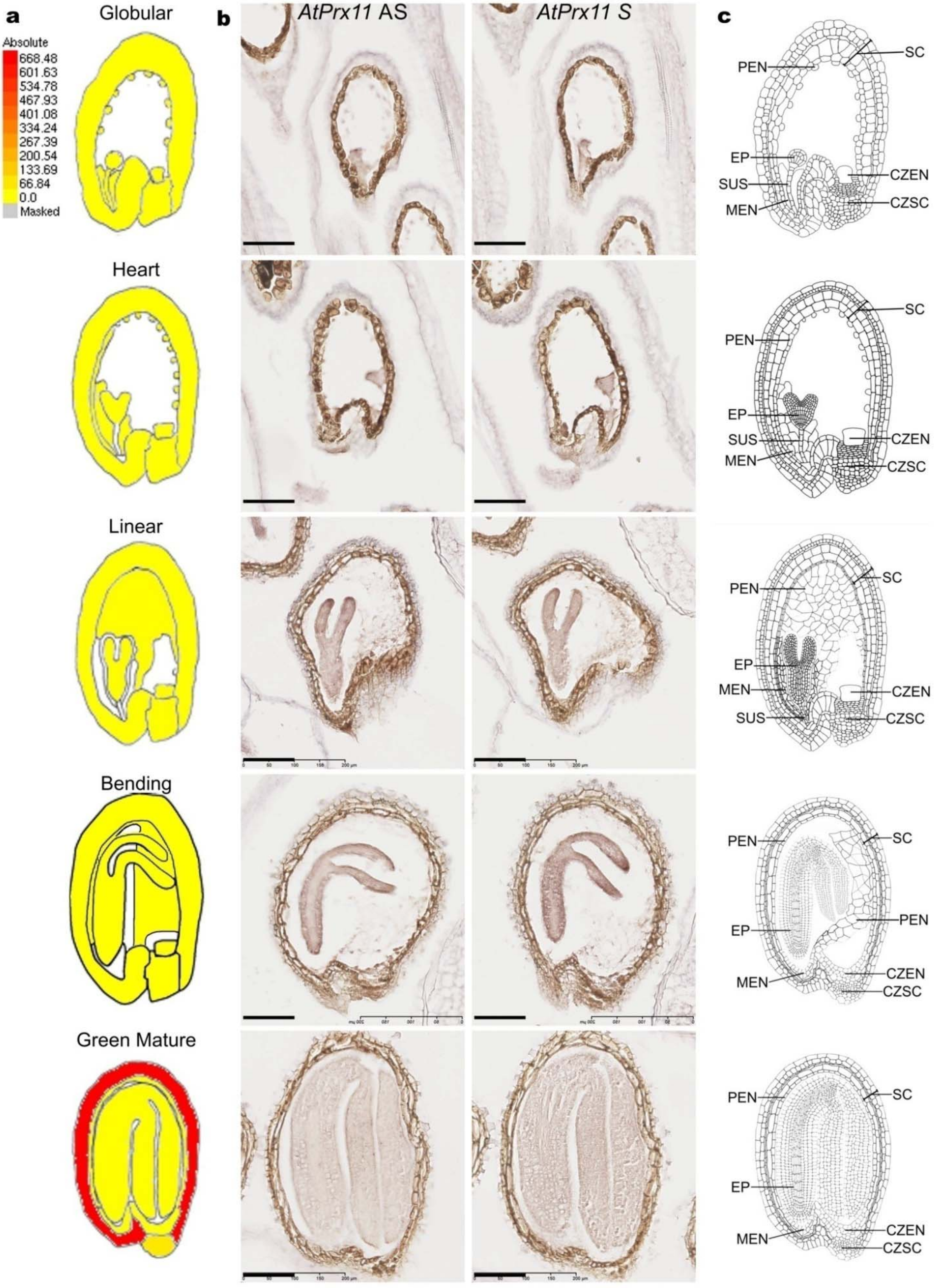

Supplementary Figure S15: *AtPRX11 (AT1G68850)*

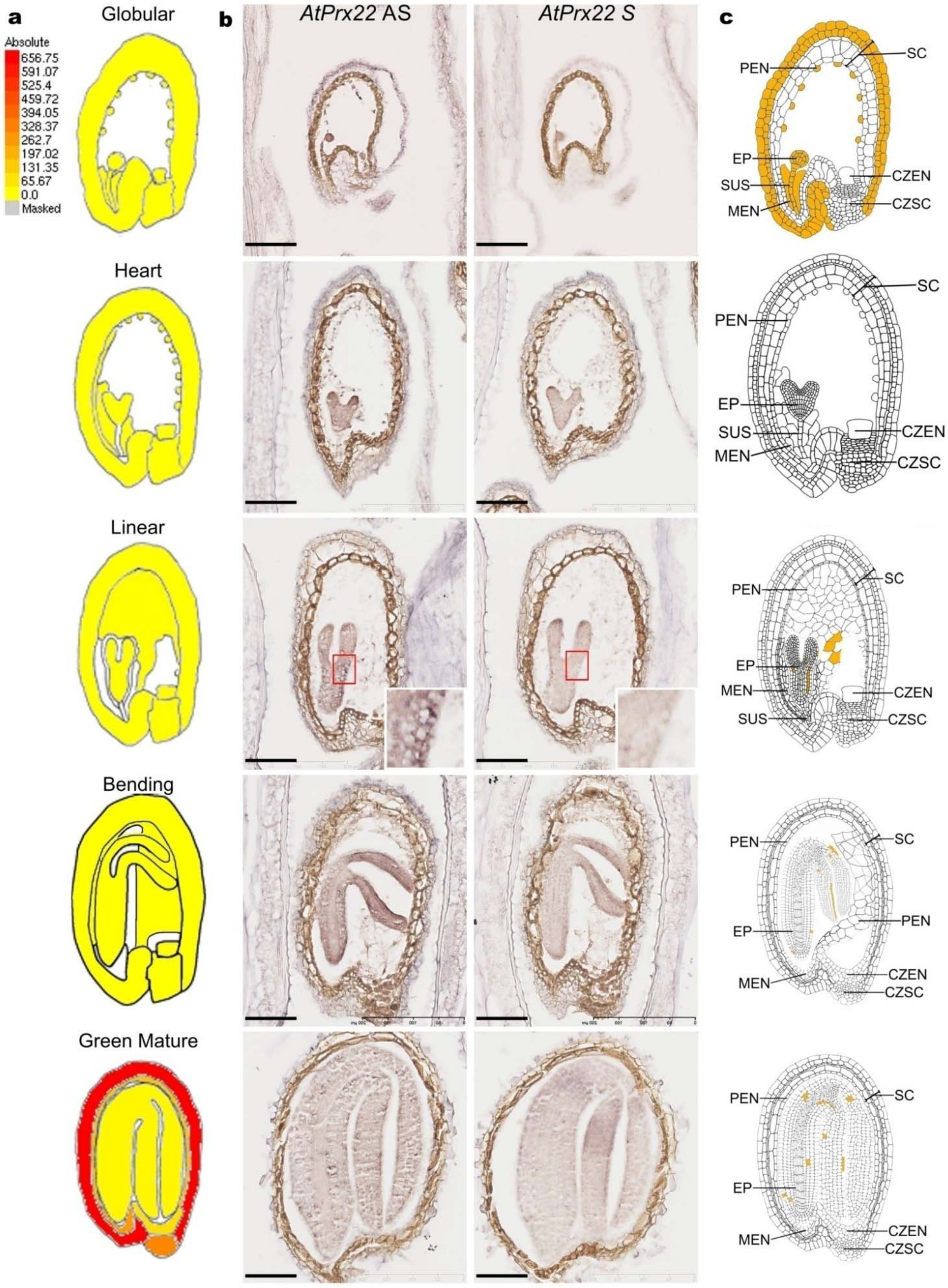

Supplementary Figure S16: *AtPRX22*
(AT2G38380)

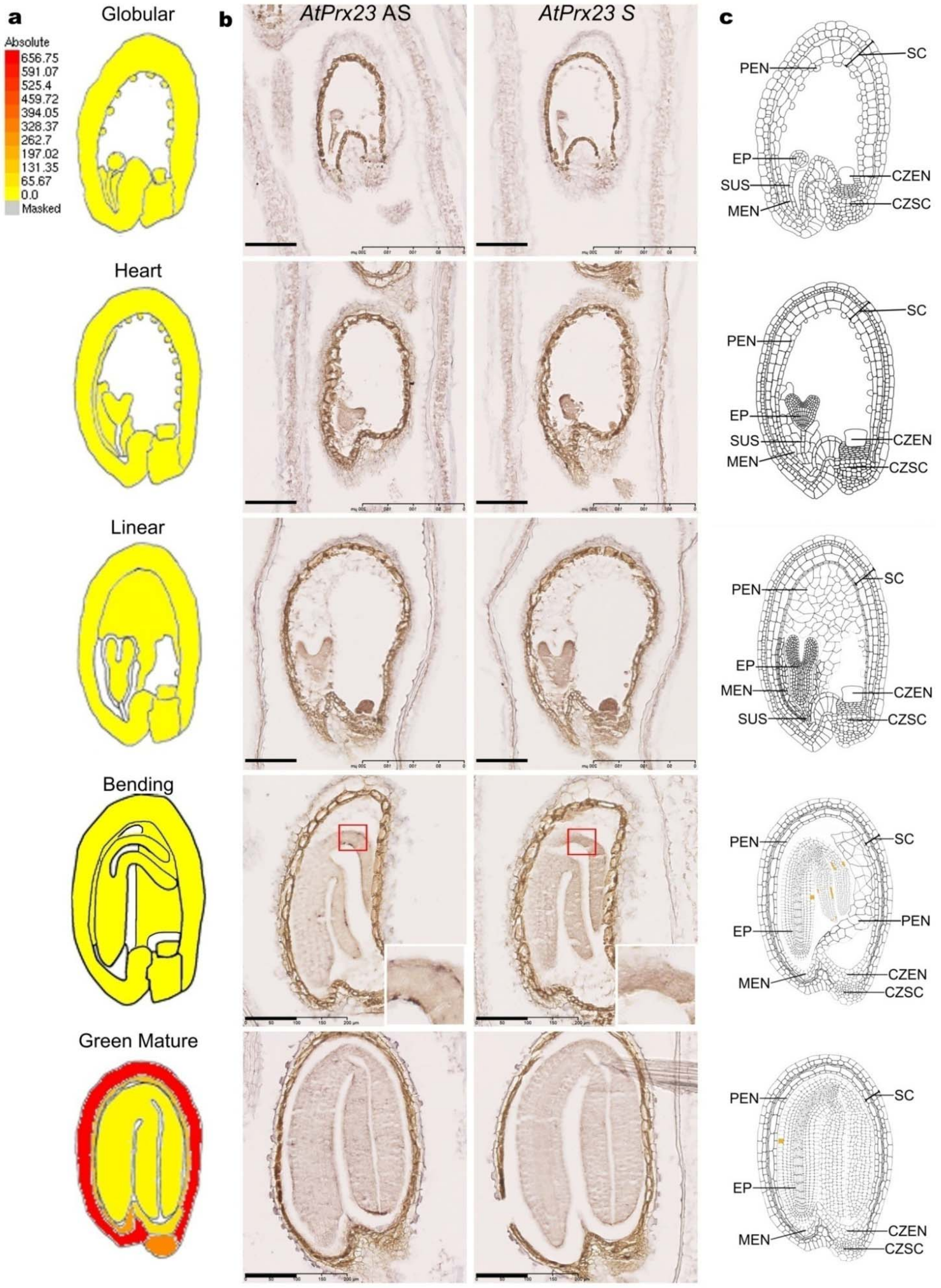

Supplementary Figure S17: *AtPRX23 (AT2G38390)*

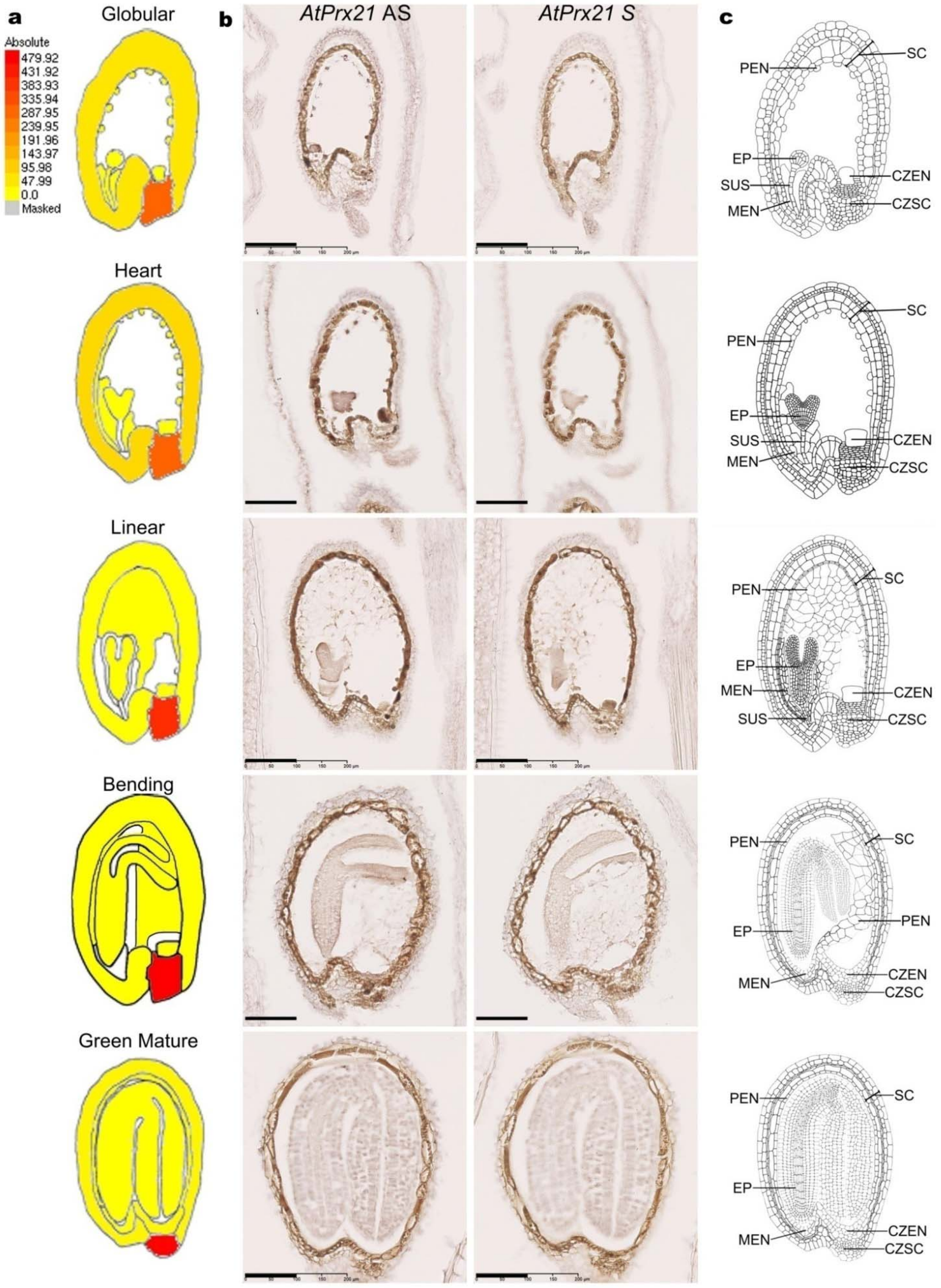

Supplementary Figure S18: *AtPRX21 (AT2G37130)*

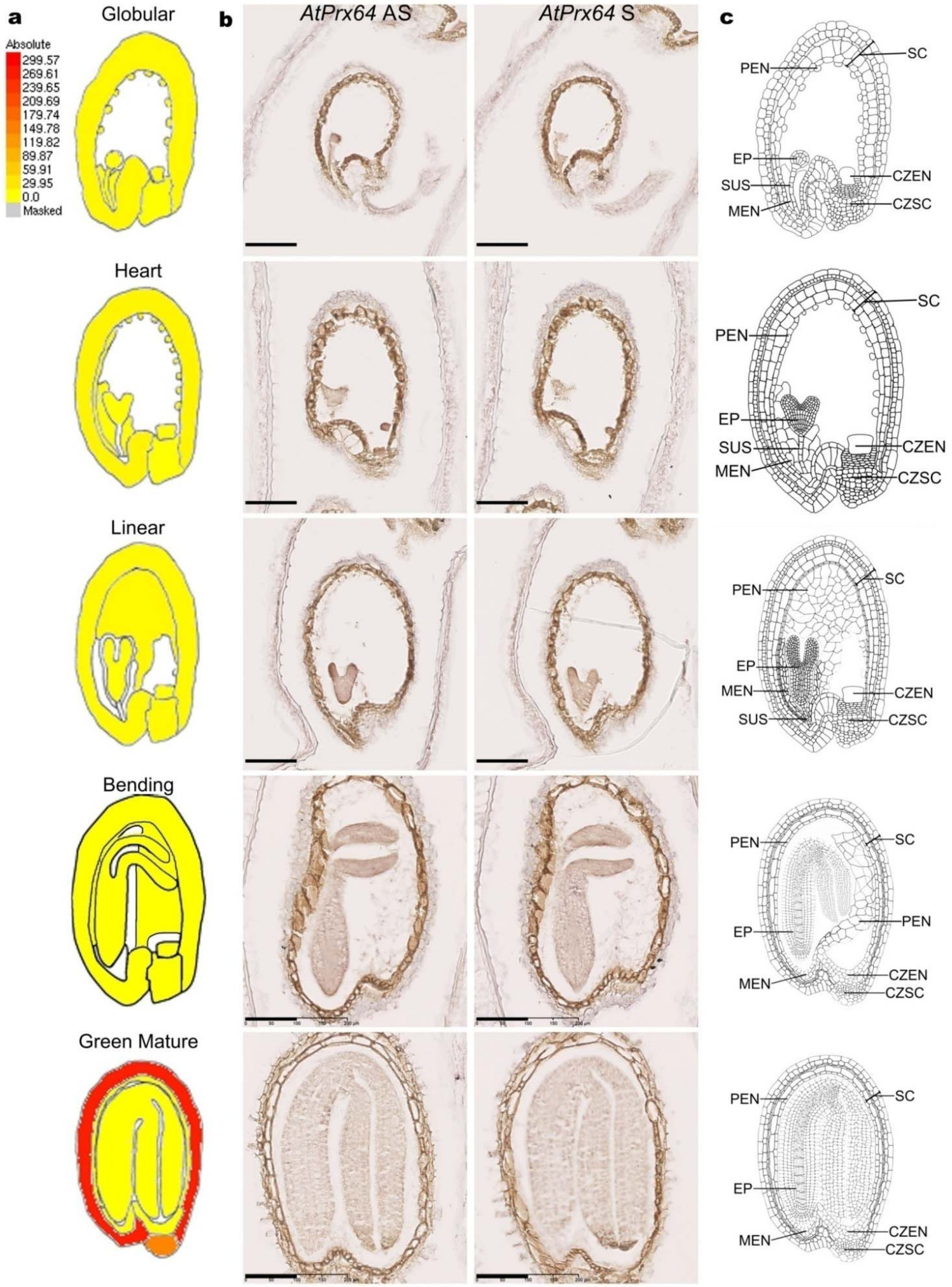

Supplementary Figure S19: *AtPRX64 (AT5G42180)*

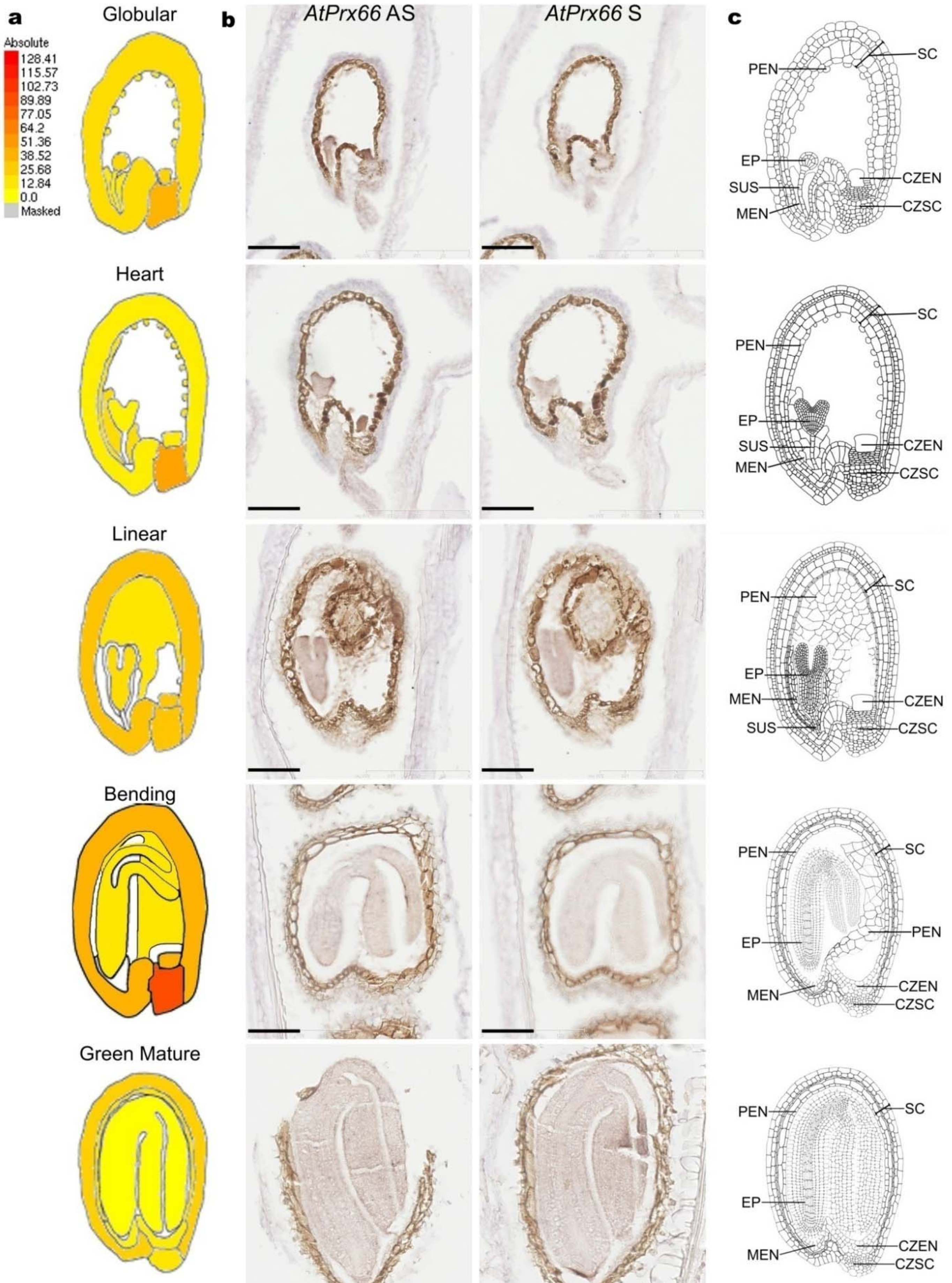

Supplementary Figure S20: *AtPRX66 (AT5G51890)*

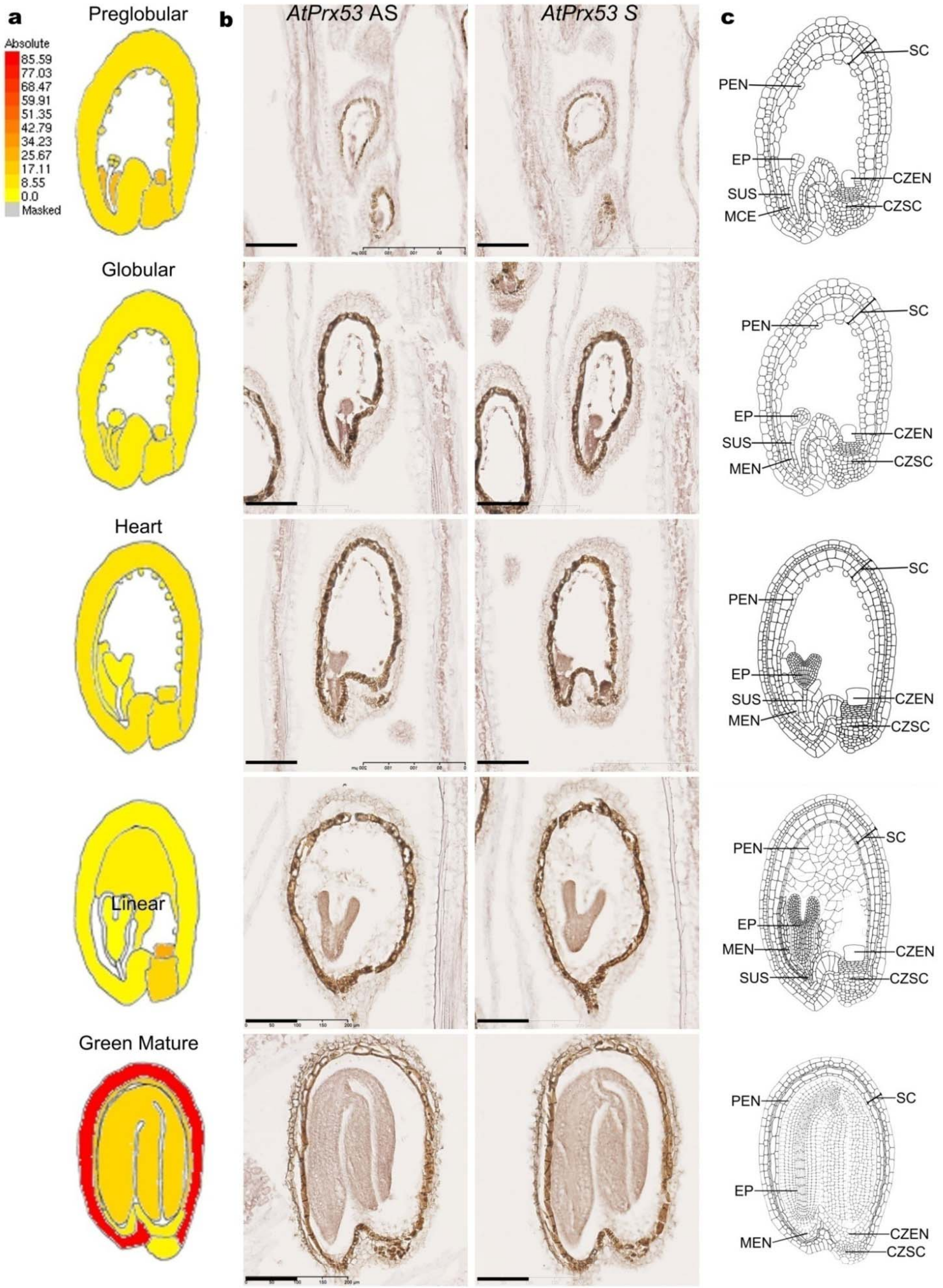

Supplementary Figure S21: *AtPRX53* *(AT5G06720)*

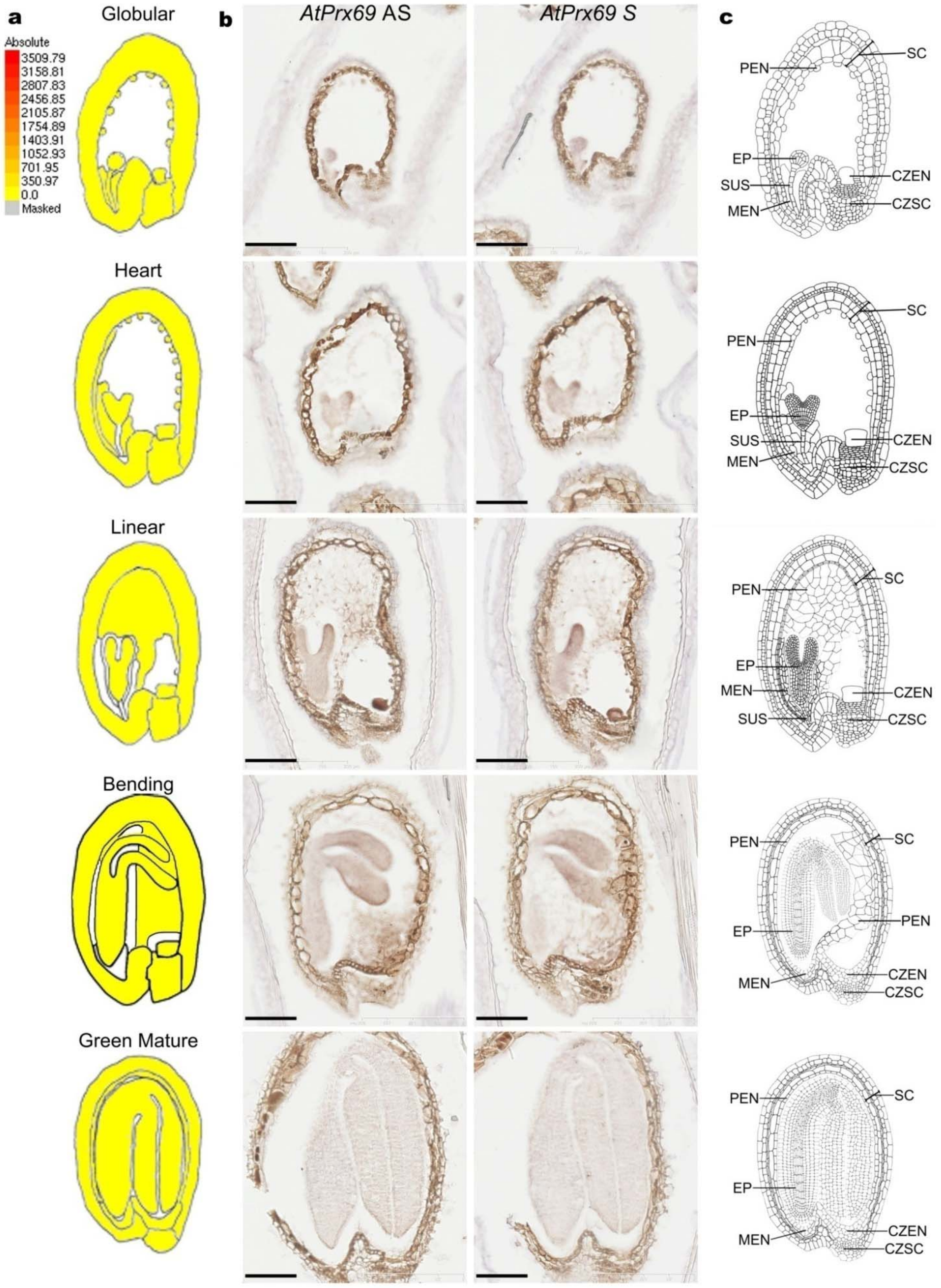

Supplementary Figure S22: *AtPRX69*
(AT5G64100)

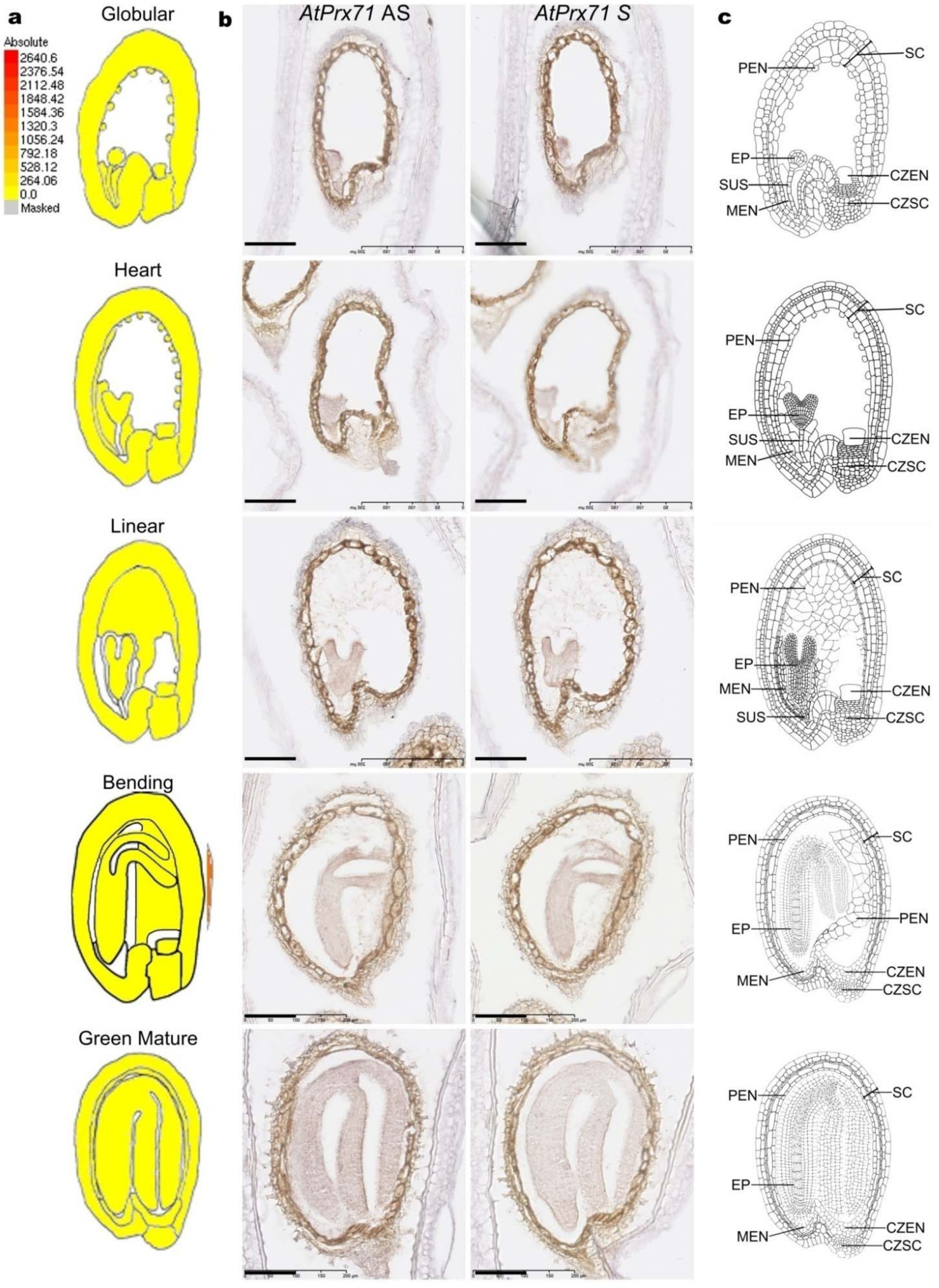

Supplementary Figure S23: *AtPRX71 (AT5G64120)*

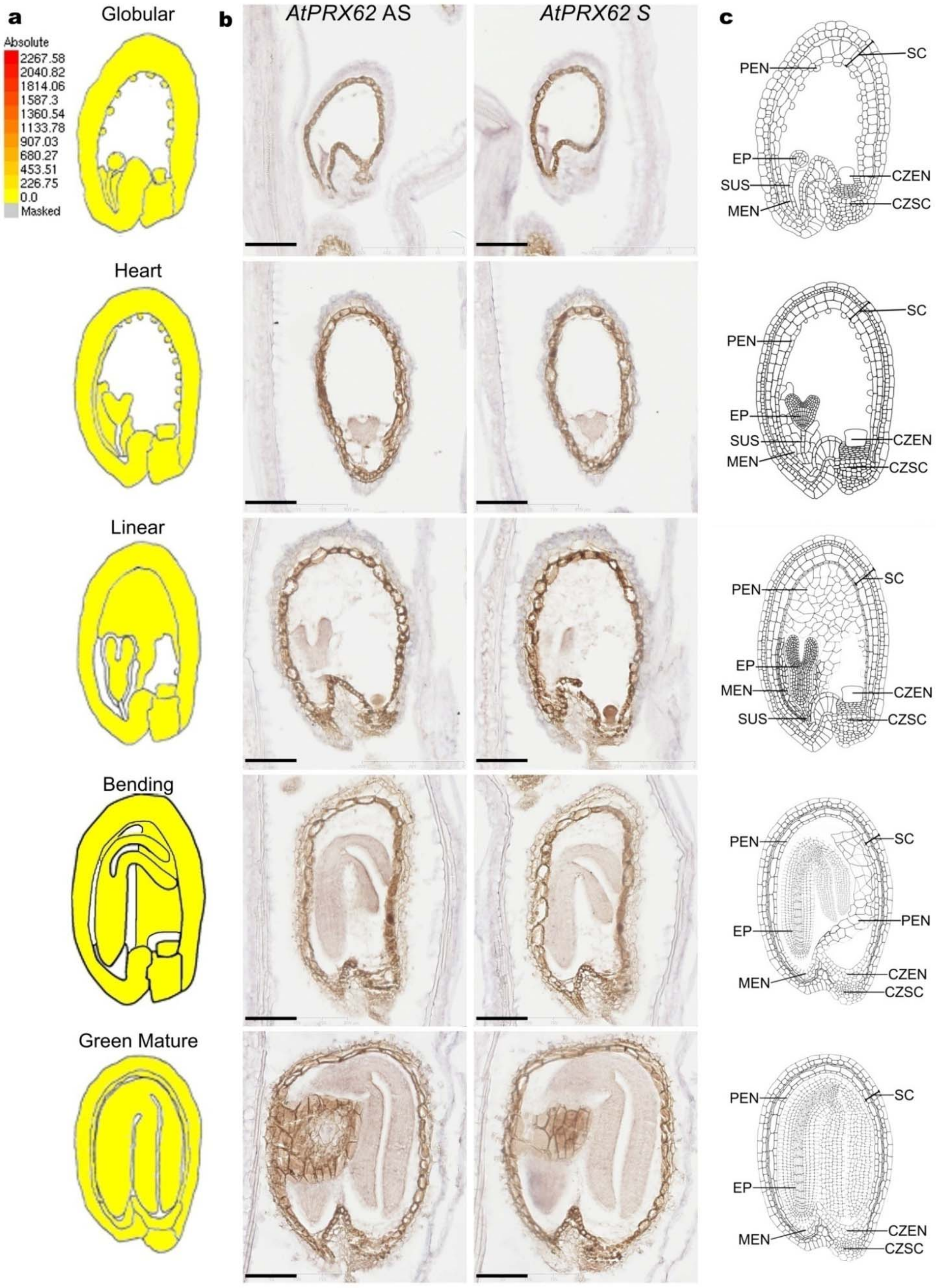

Supplementary Figure S24: *AtPRX62 (AT5G39580)*

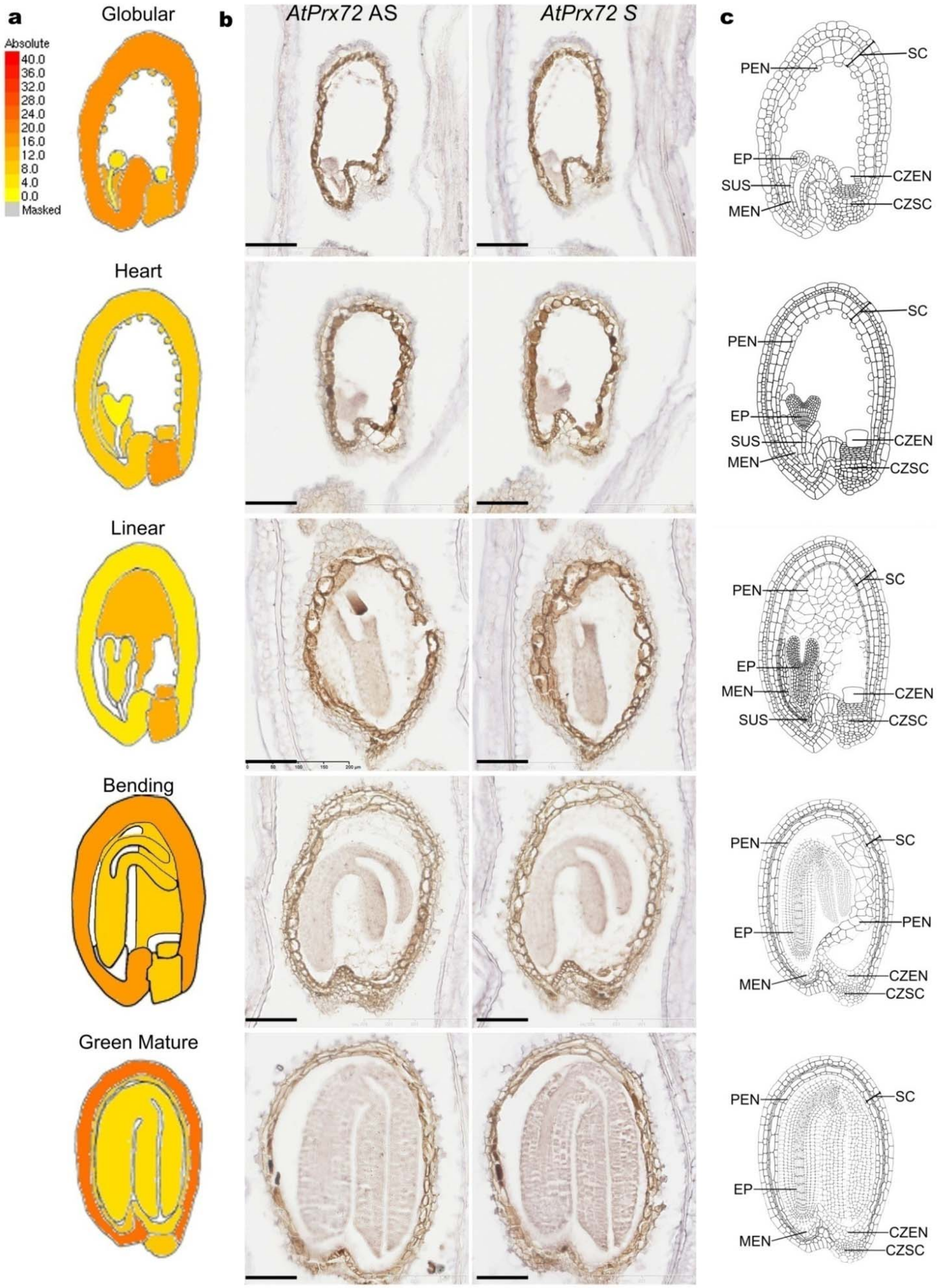

Supplementary Figure S25: *AtPRX72* *(AT5G66390)*

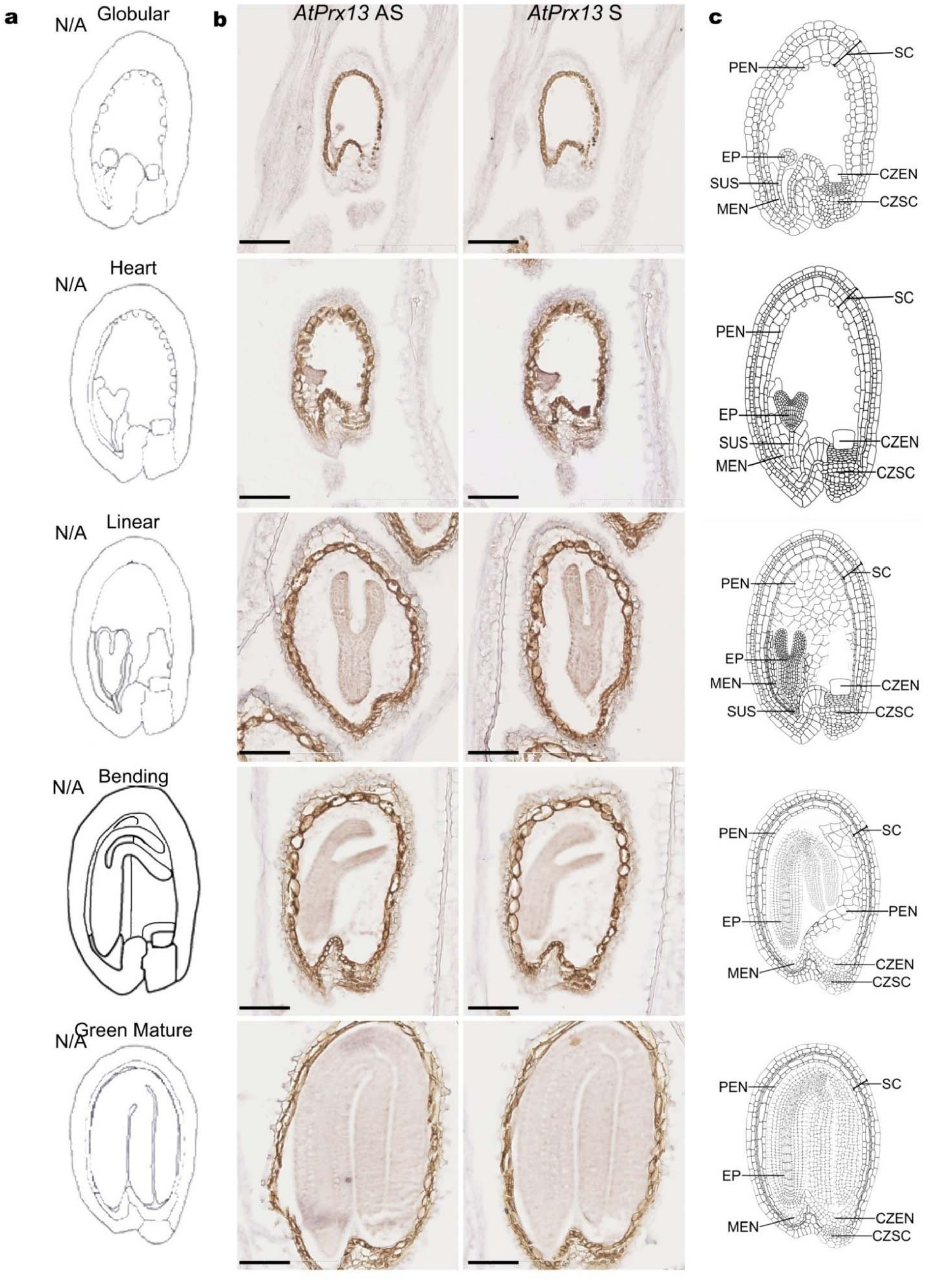

Supplementary Figure S26: *AtPRX13*
(AT1G77100)

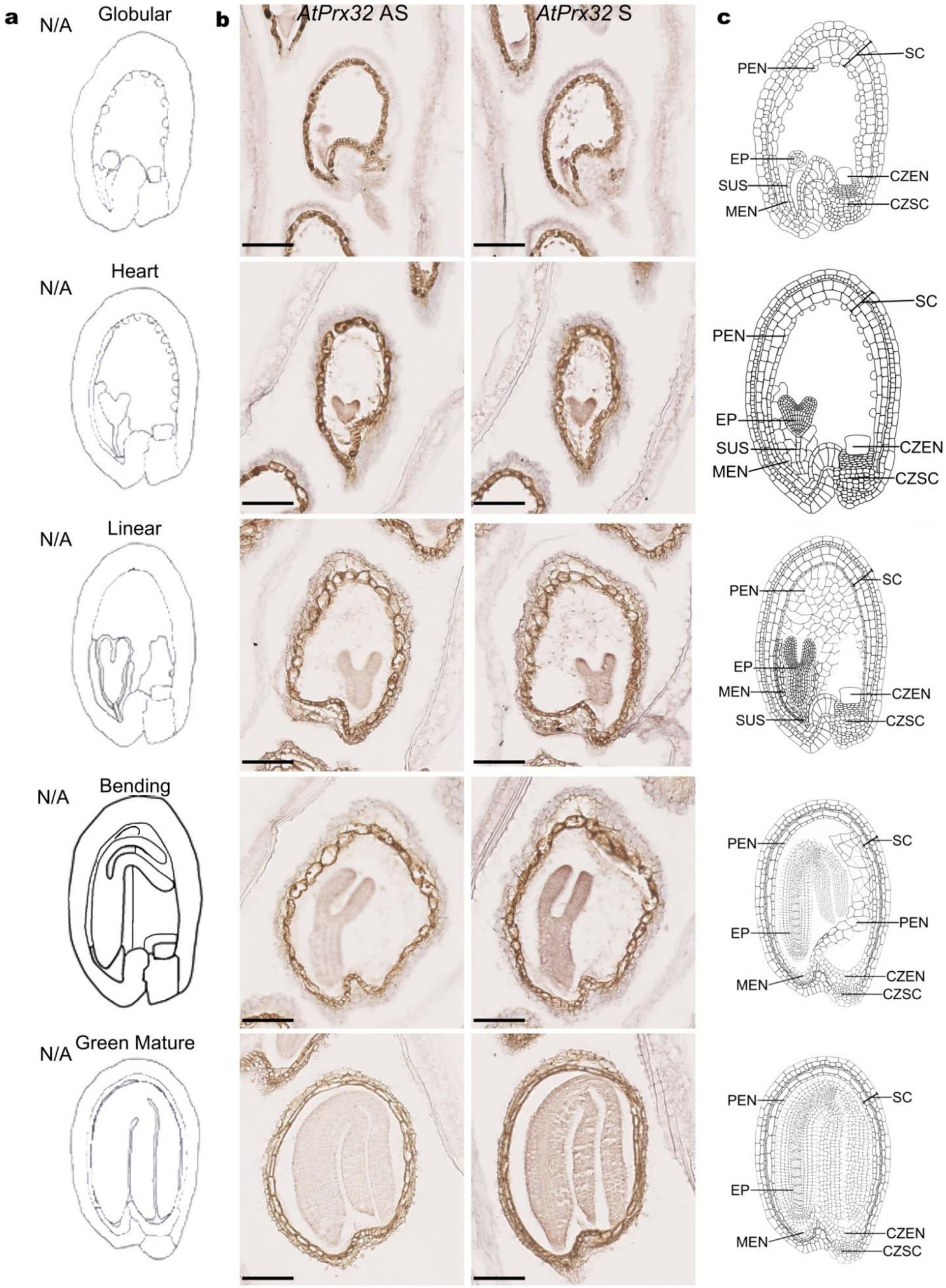

Supplementary Figure S27: *AtPRX32 (AT3G32980)*

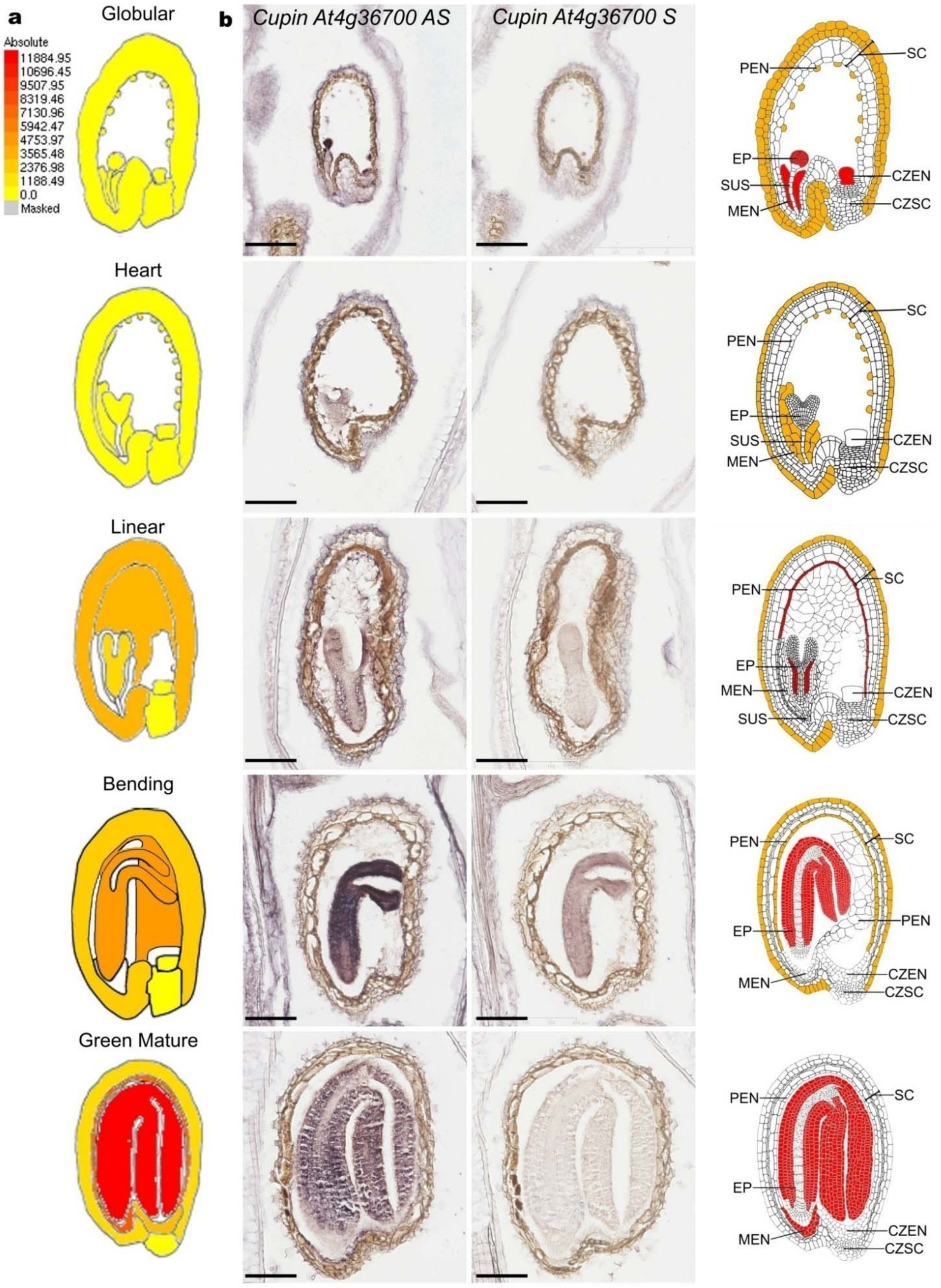

Supplementary Figure S28: *Cupin (AT4G36700)*

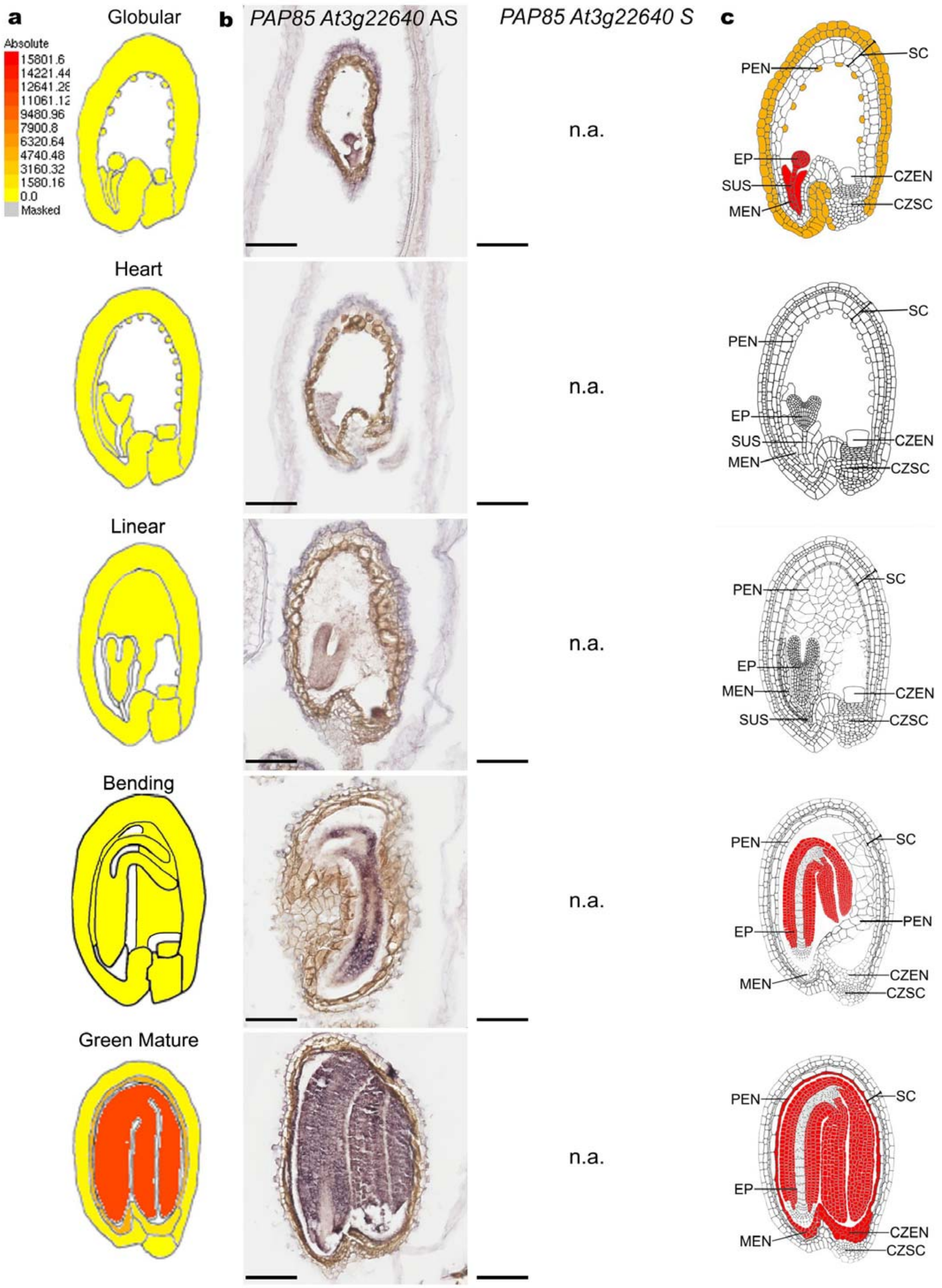

Supplementary Figure S29: *PAP85 (AT3G22640)*

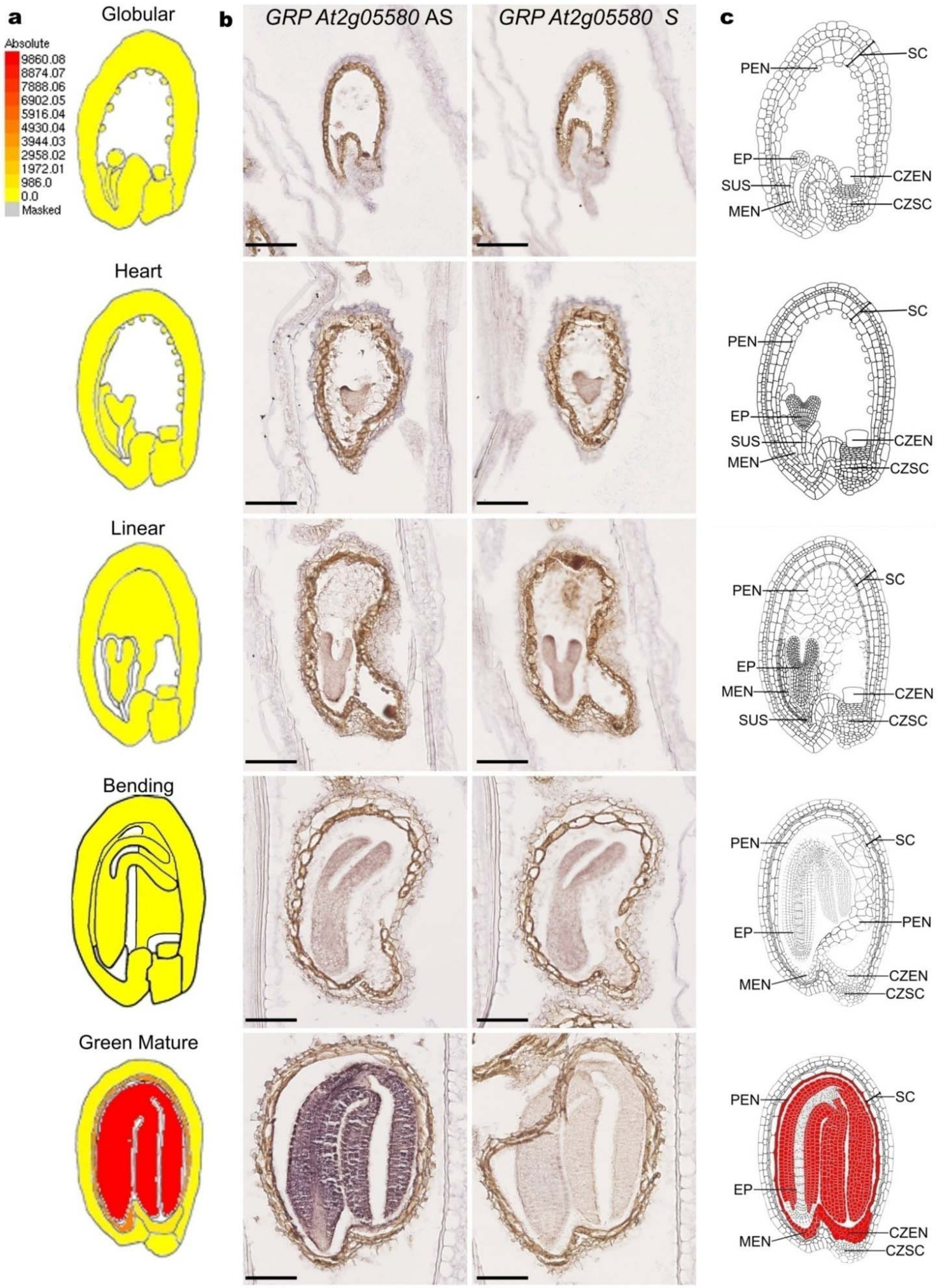

Supplementary Figure S30: *GRP (AT2G05580)*

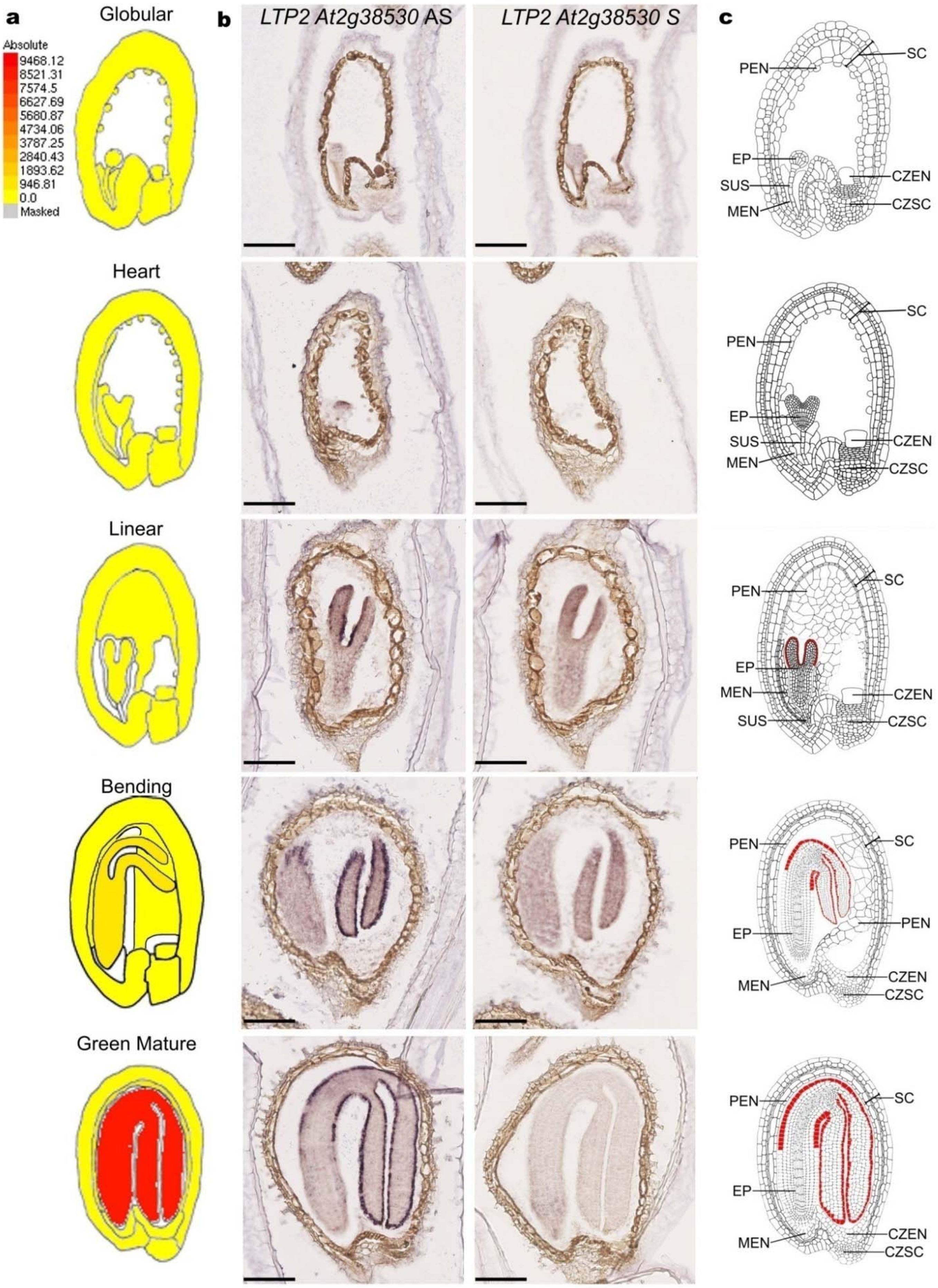

Supplementary Figure S31: *LTP2 (AT2G38530)*

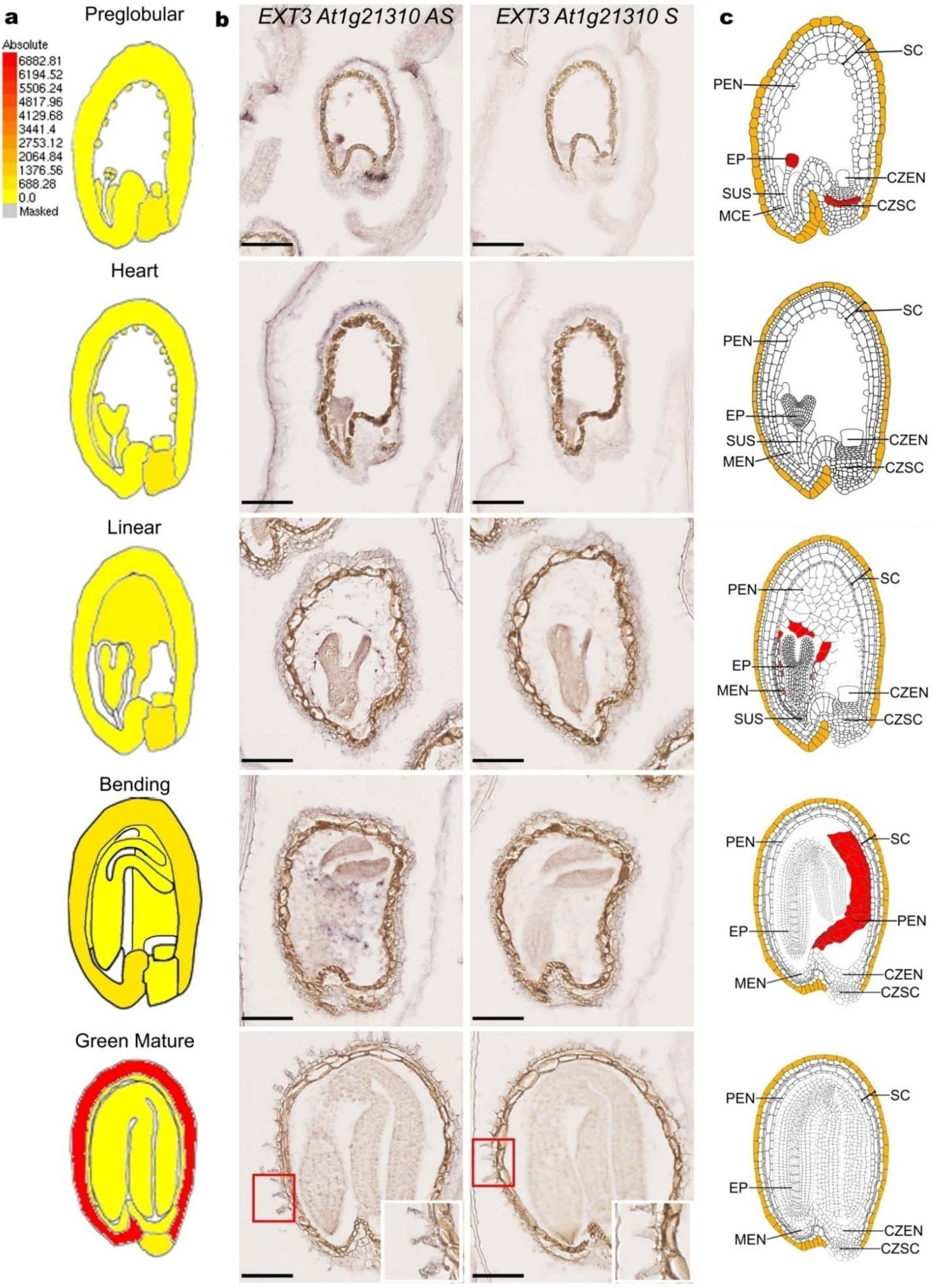

Supplementary Figure S32: *EXT3 (AT1G21310)*

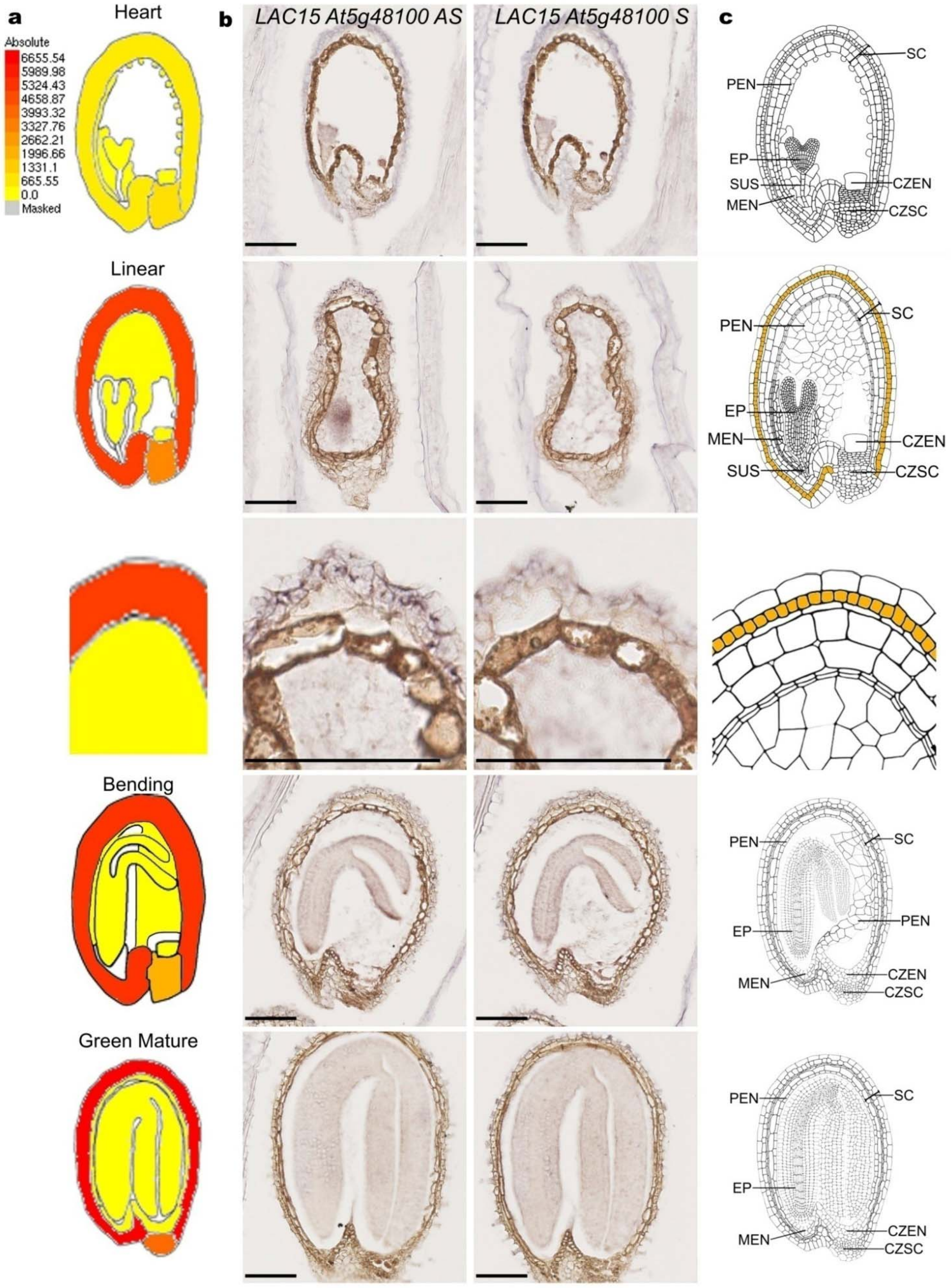

Supplementary Figure S33: *LAC15 (AT5G48100)*

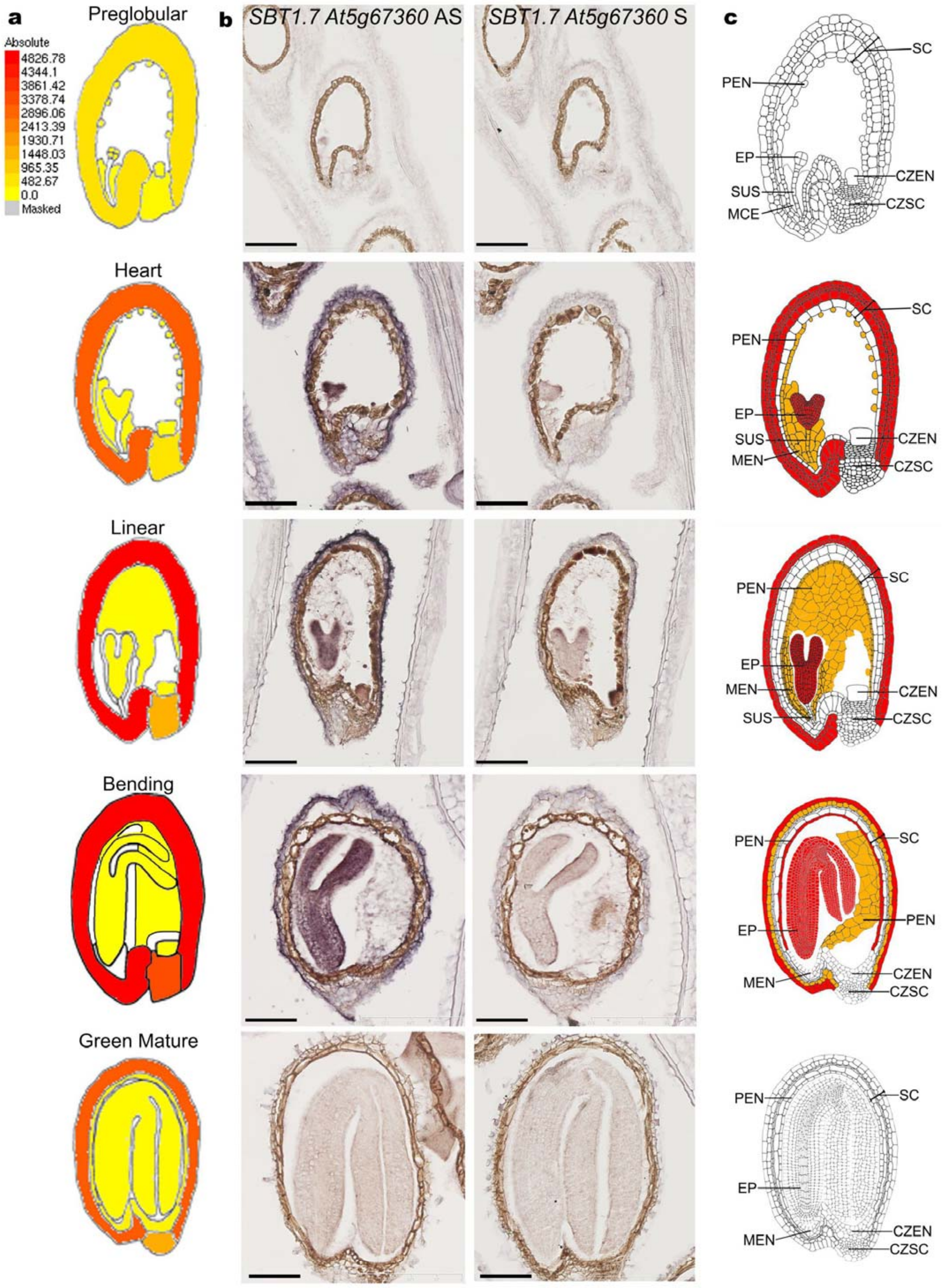

Supplementary Figure S34: *SBT1.7 (AT5G67360)*

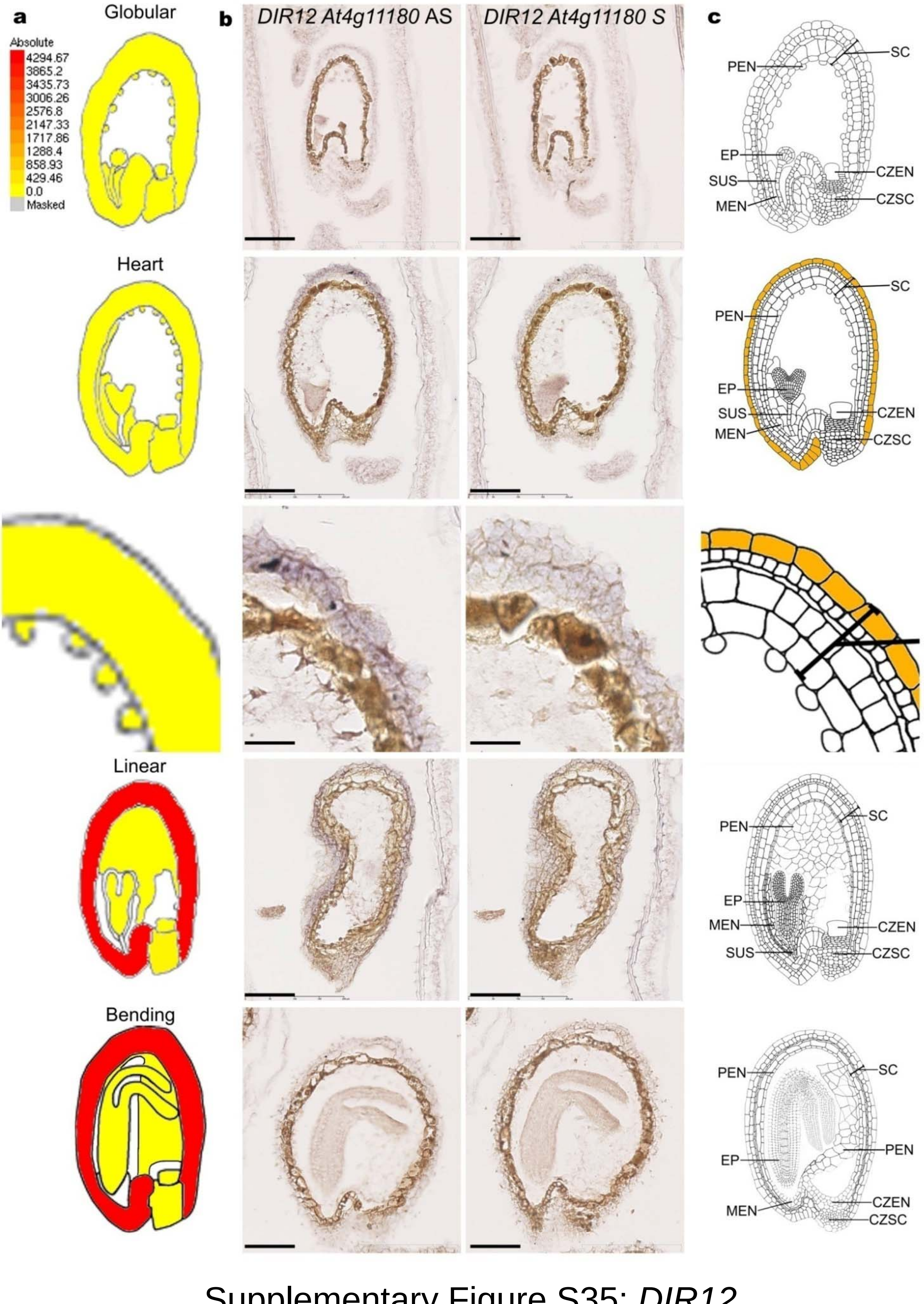

Supplementary Figure S35: *DIR12 (AT4G11180)*

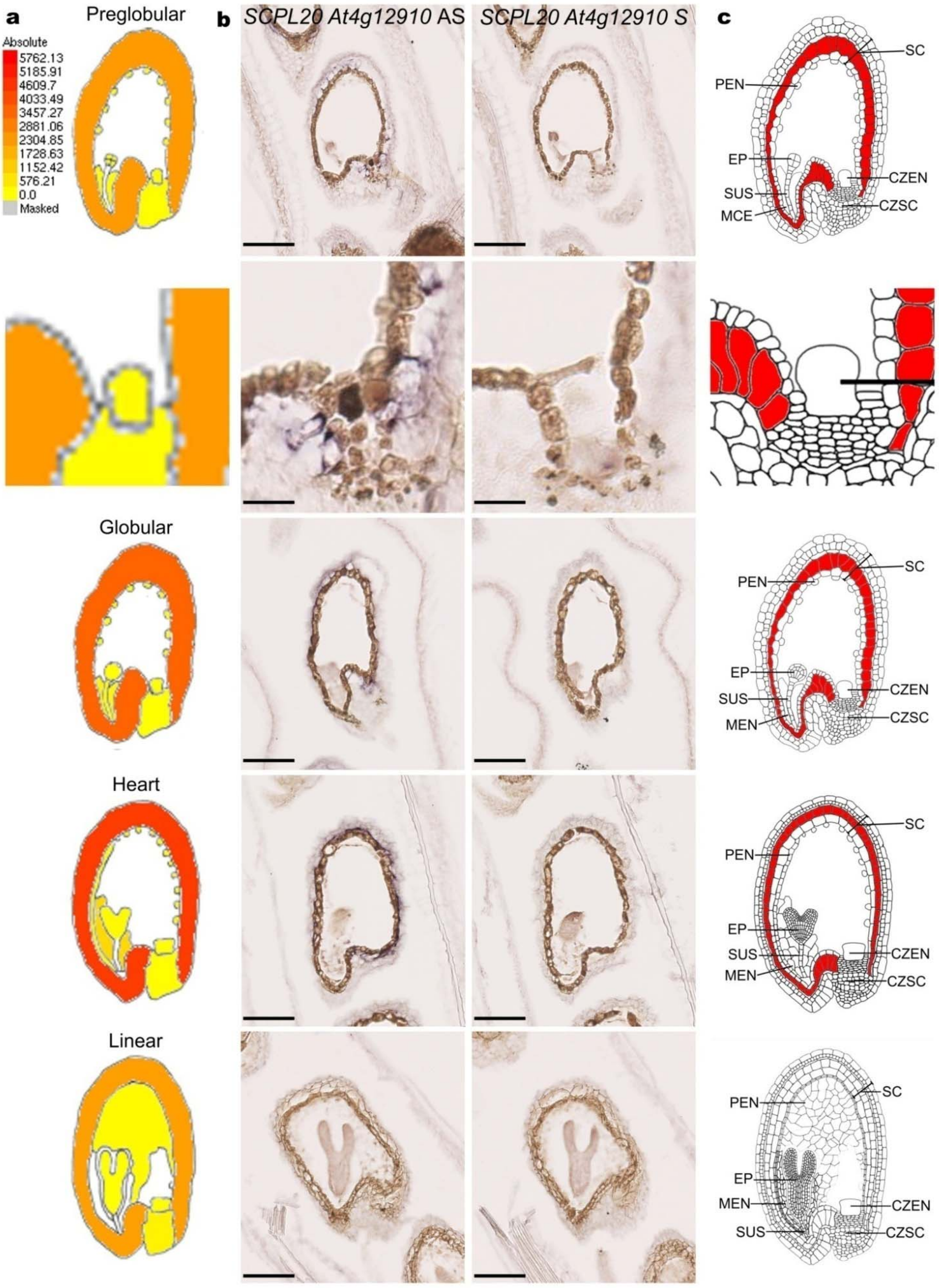

Supplementary Figure S36: *SCPL20 (AT4G12910)*

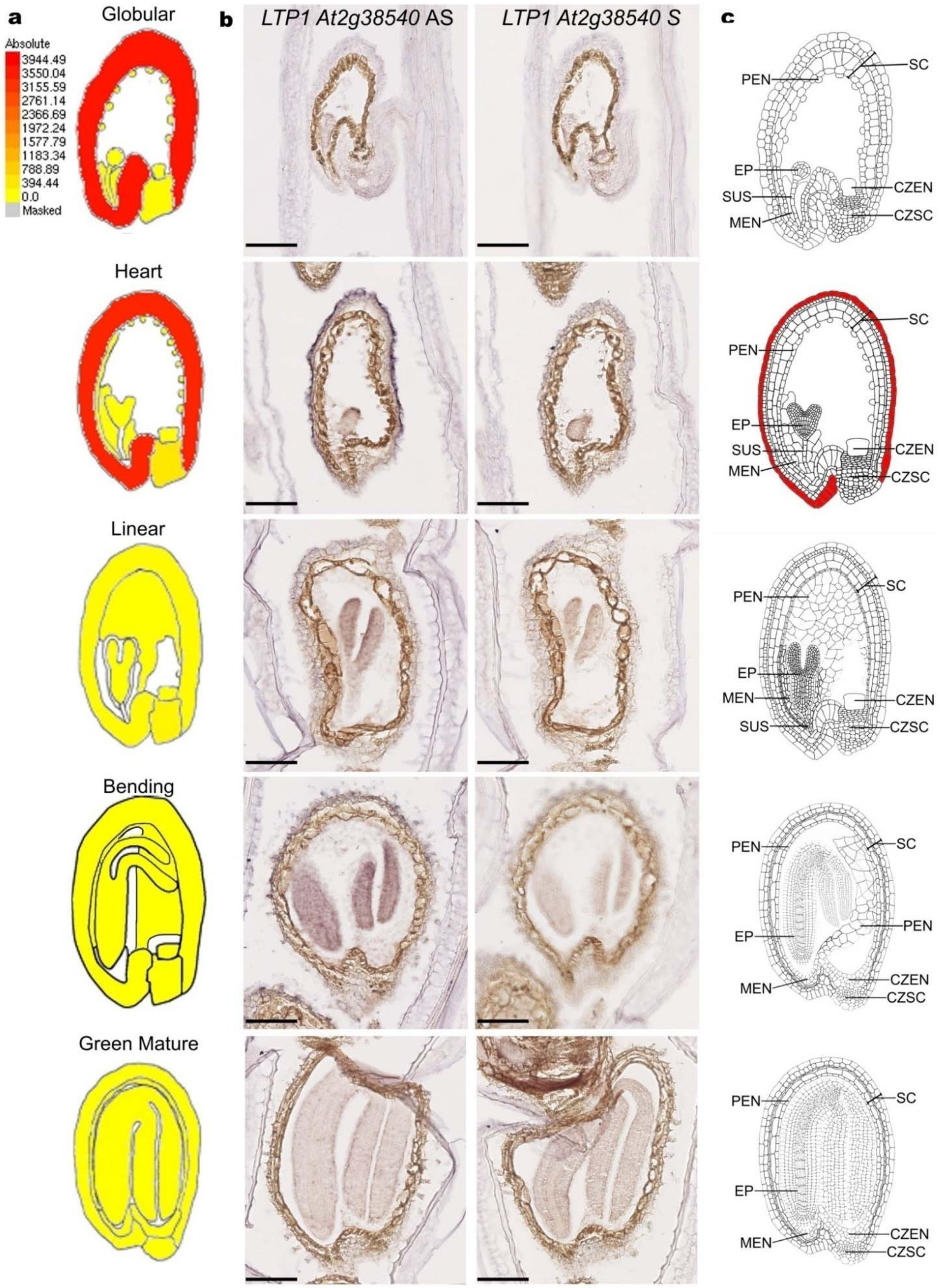

Supplementary Figure S37: *LTP1 (AT2G38540)*

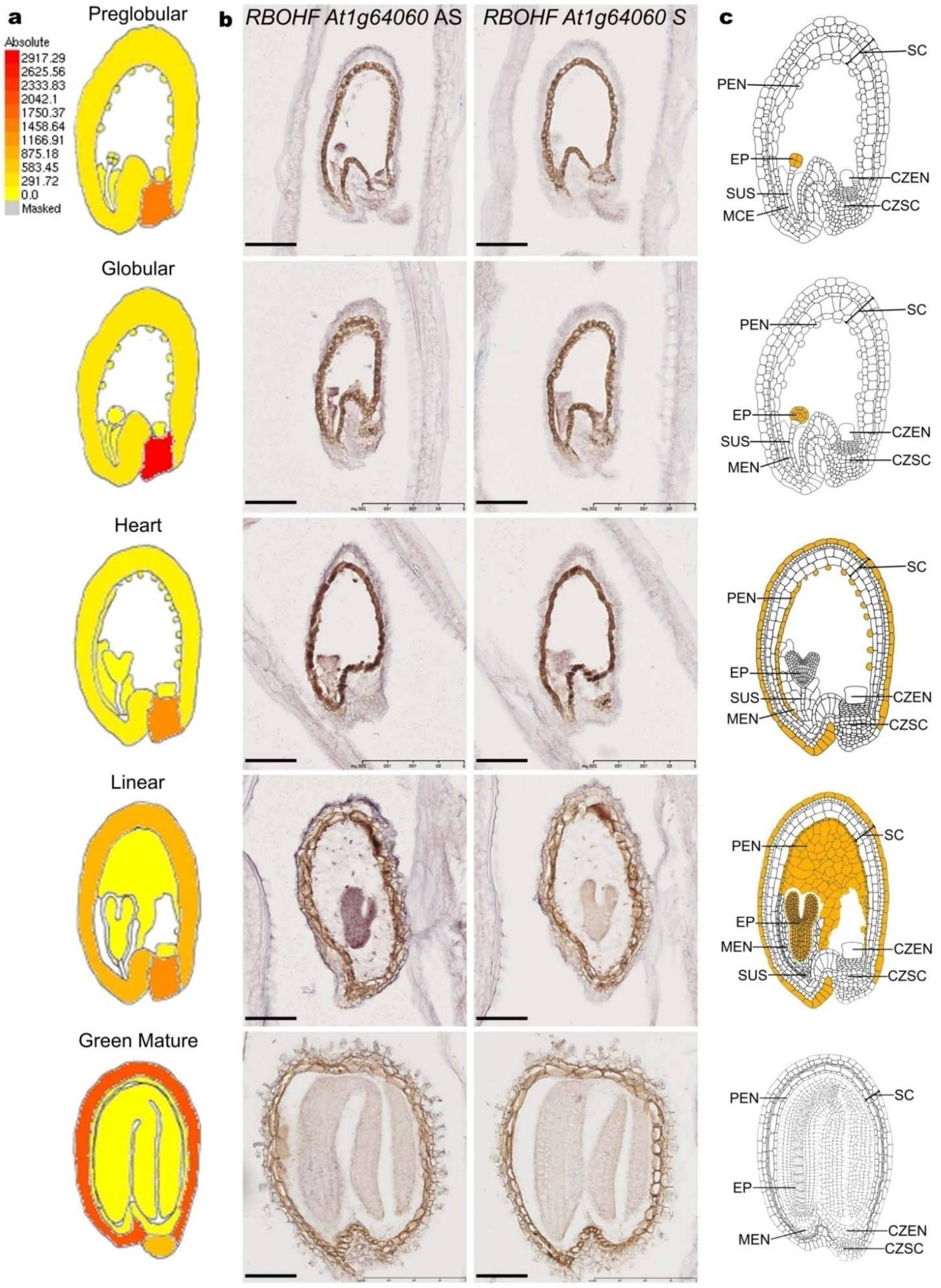

Supplementary Figure S38: *RBOHF (AT1G64060)*

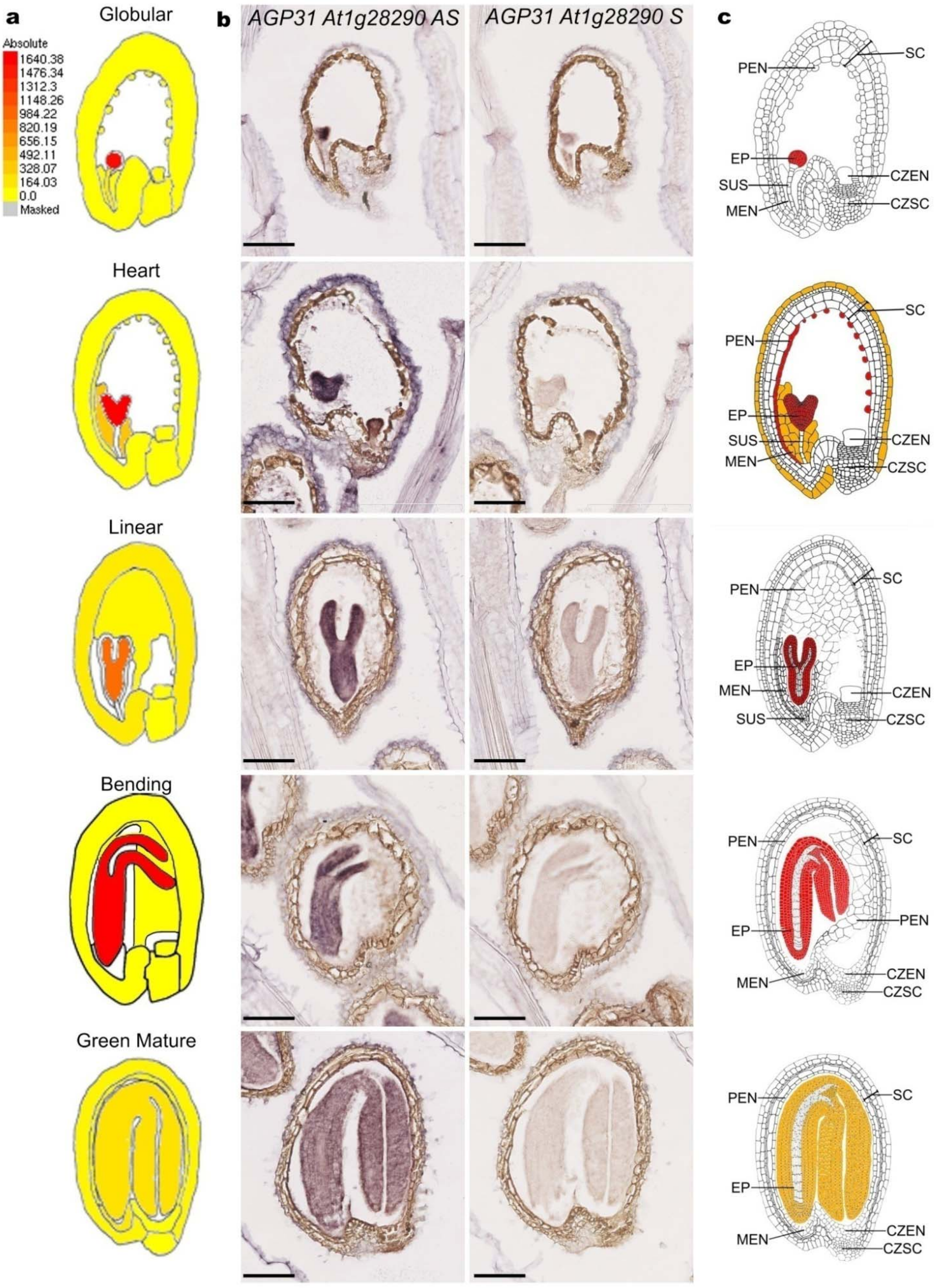

Supplementary Figure S39: *AGP31* *(AT1G28290)*

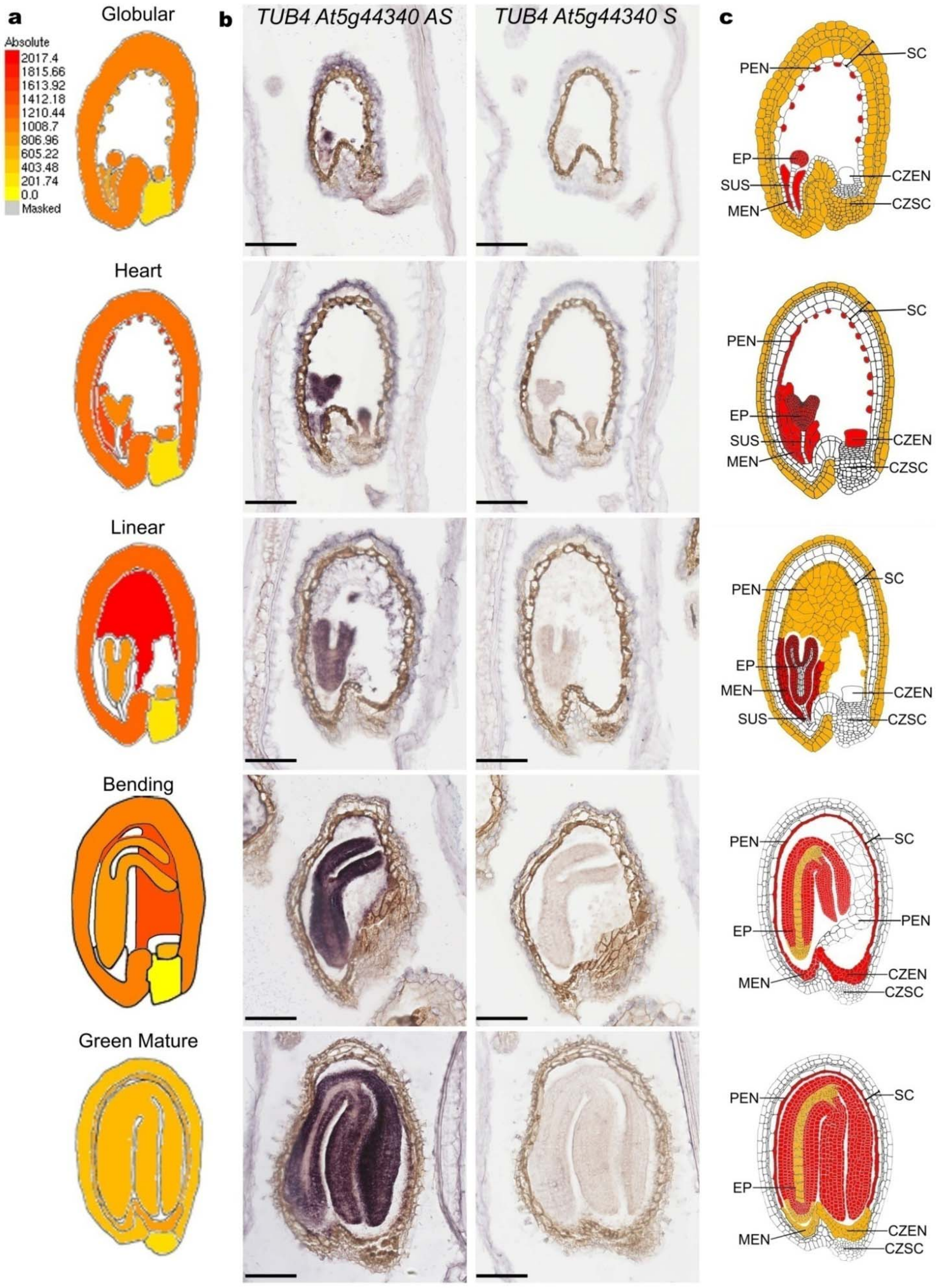

Supplementary Figure S40: *TUB4 (AT5G44340)*

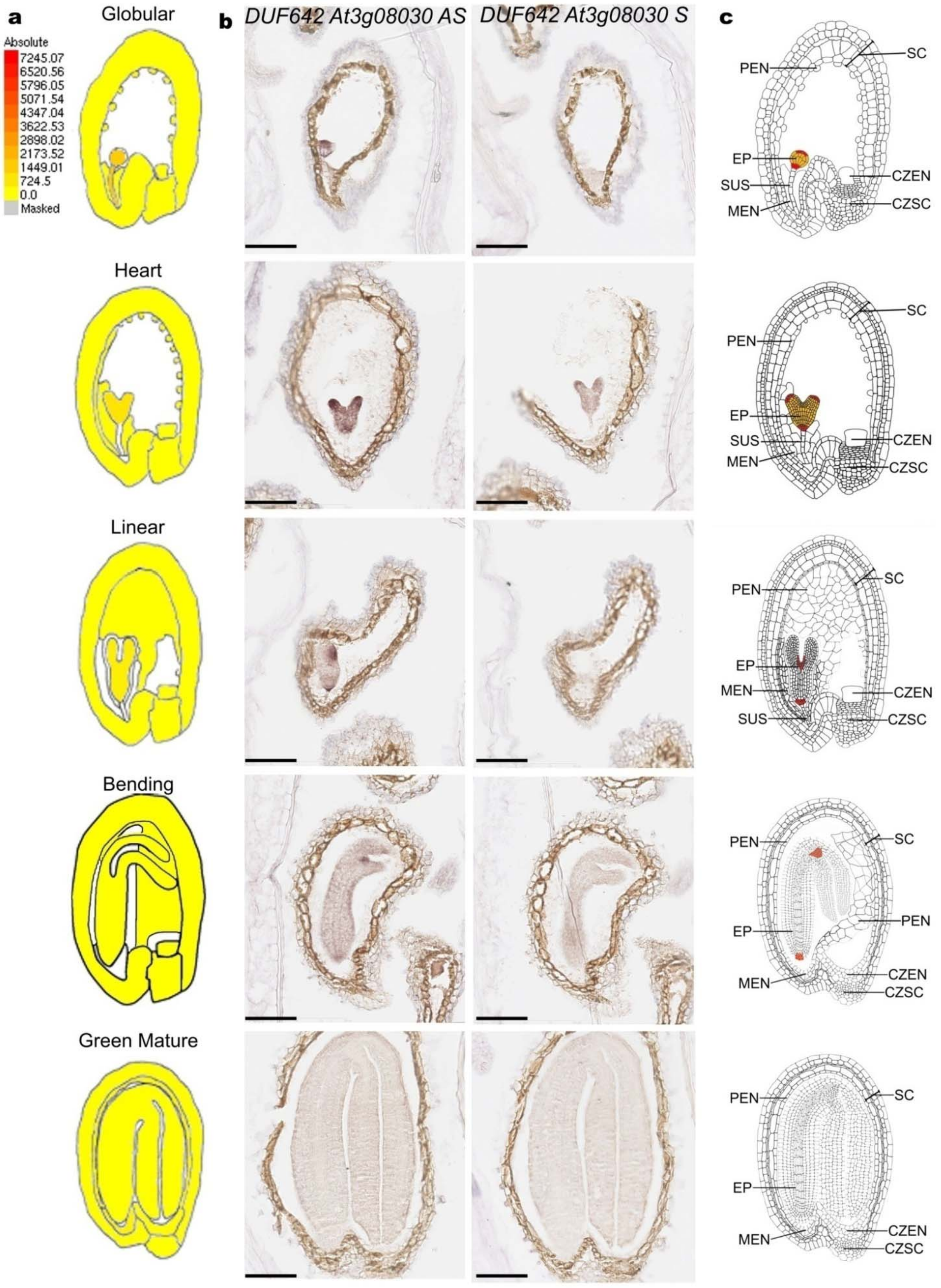

Supplementary Figure S41: *DUF642* *(AT3G08030)*

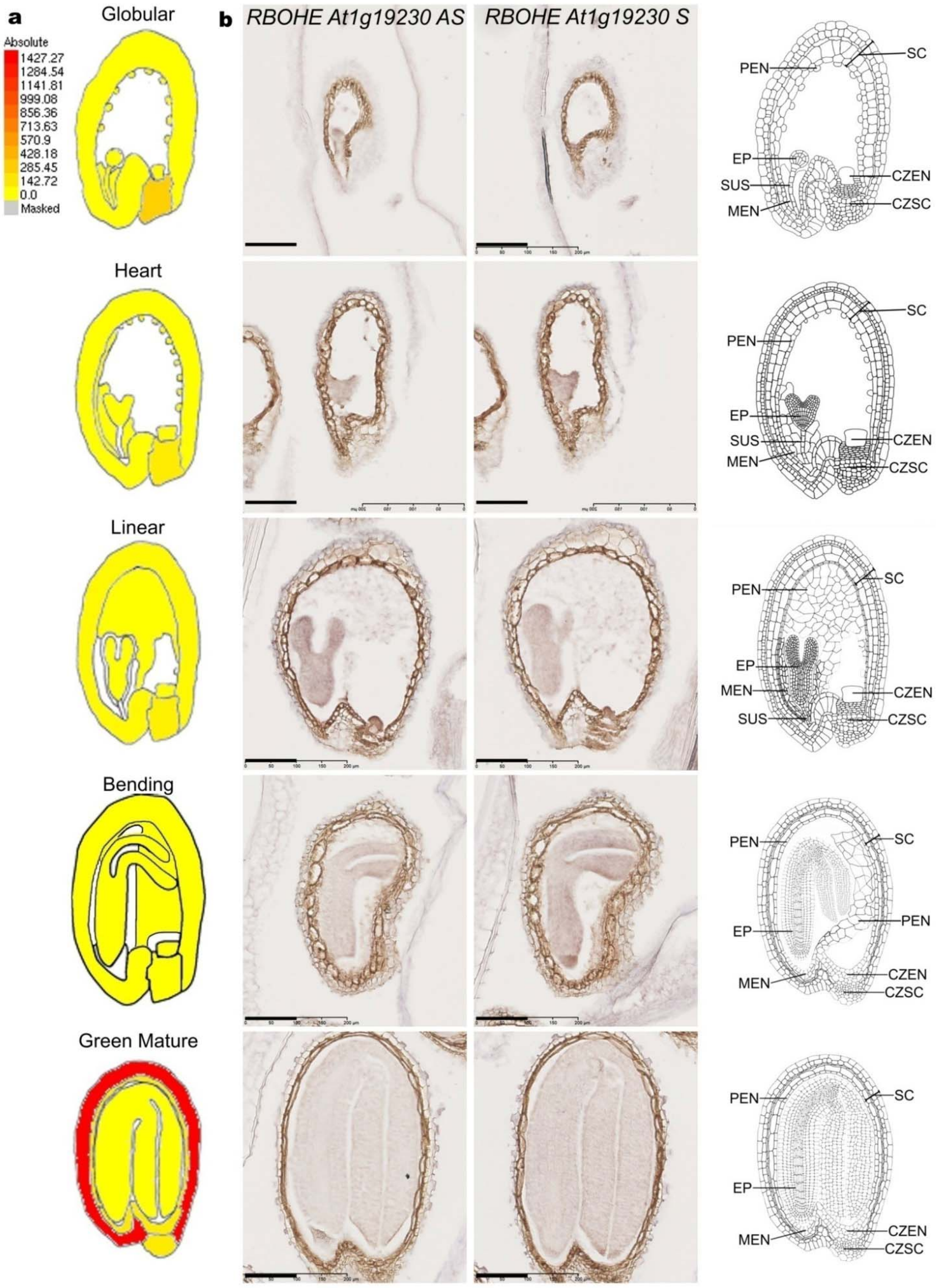

Supplementary Figure S42: *RBOHE (AT1G19230)*

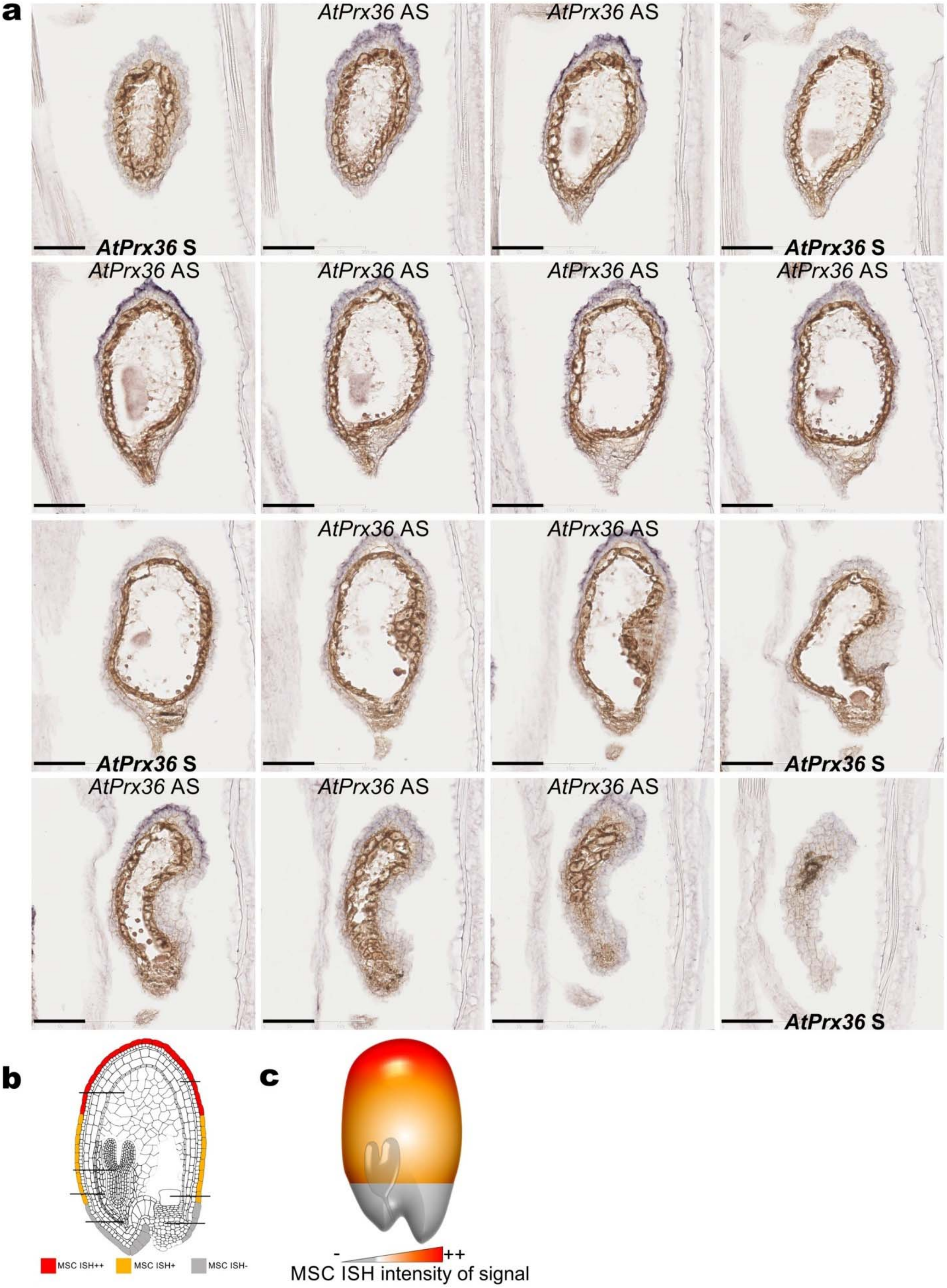

Supplementary Figure S43: Illustration of the possibility to use serial sections for a same riboprobe pair to draw tomographical view of *in situ* hybridization (ISH) signals.
(a) 16 serial section of the same tissue array were hybridized either with *AtPRX36* antisense (AS, labelled on top of images) or sense (S, labelled on the bottom of images) probes, and serial sections from the same seed is displayed. **(b)** "flat" ISH map and **(c)** "3D-like" reconstituted ISH map using the same colour code as in **Supplementary Figures S4-S42.** Scale bars: 100µm.

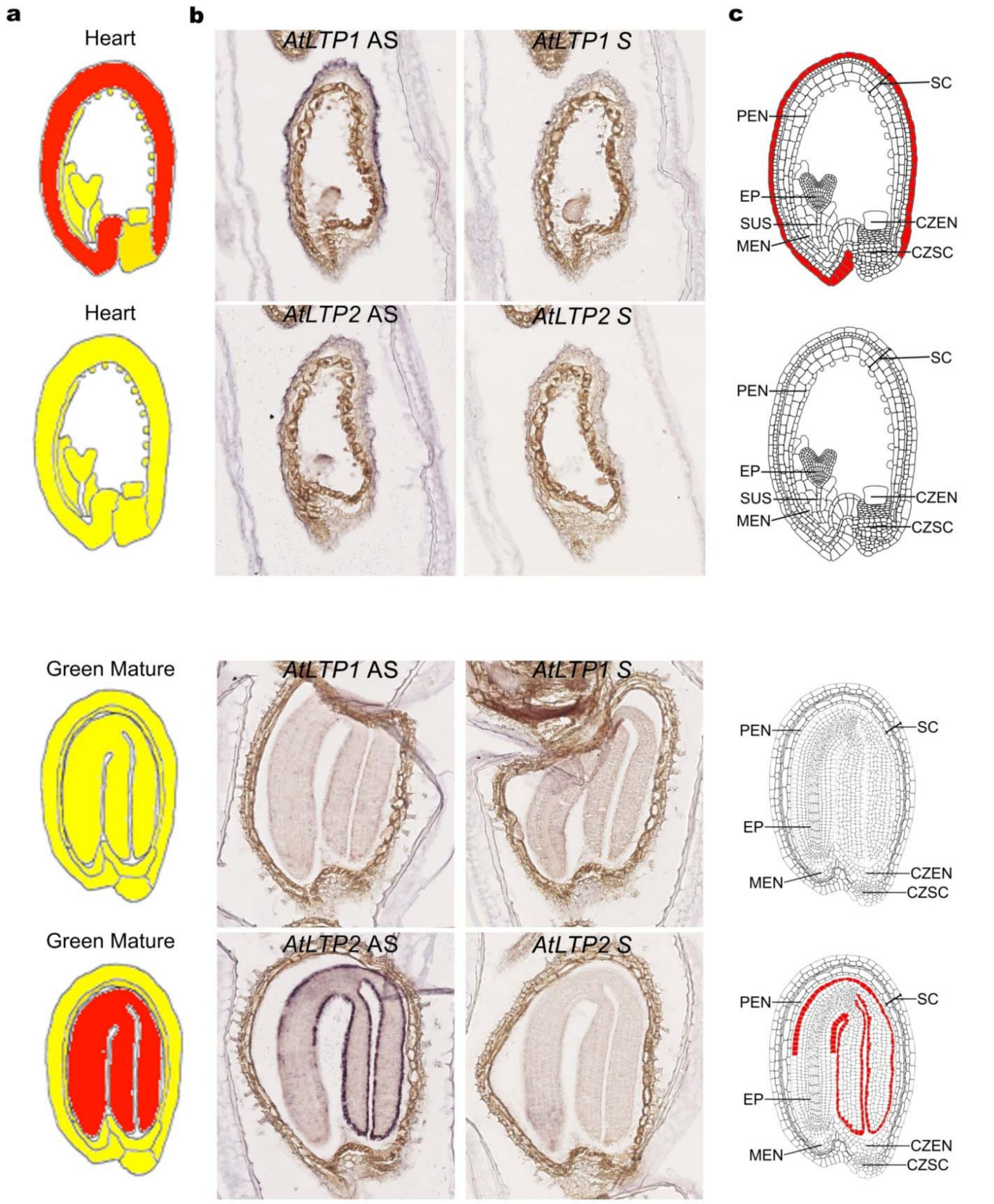

Supplementary Figure S44: Illustration of the specificity of *in situ* hybridization (ISH) signals for two duplicated genes (*LTP1* and *LTP2*) presenting 83.2% nucleotide sequence identity.
(a) a screen copy of the seed eFP browser tissue-specific microtranscriptomic map including the individual absolute heatmap scale (red-to-yellow colour codes correspond to high-to-low expression values) that is different for each gene (Winter *et al.,* 2007), **(b)** the corresponding ISH results for both the antisense (AS) and sense (S) probe used as a negative control, and **(c)** the final corresponding new cell-specific ISH map re-coloured after original drawings from Belmonte *et al*. (2013) available at Seedgenenetwork (http://estdb.biology.ucla.edu/ seed/) giving increased cellular resolution and using a unique colour code for all genes with red corresponding to strong signals, orange corresponding to moderate signals and white corresponding to the absence of detected ISH signal. Note that ISH on serial section of the same developping seeds detects spatiotemporally distinct signals for these two genes. See **Supplementary Table S8** for details on the sequence identities.

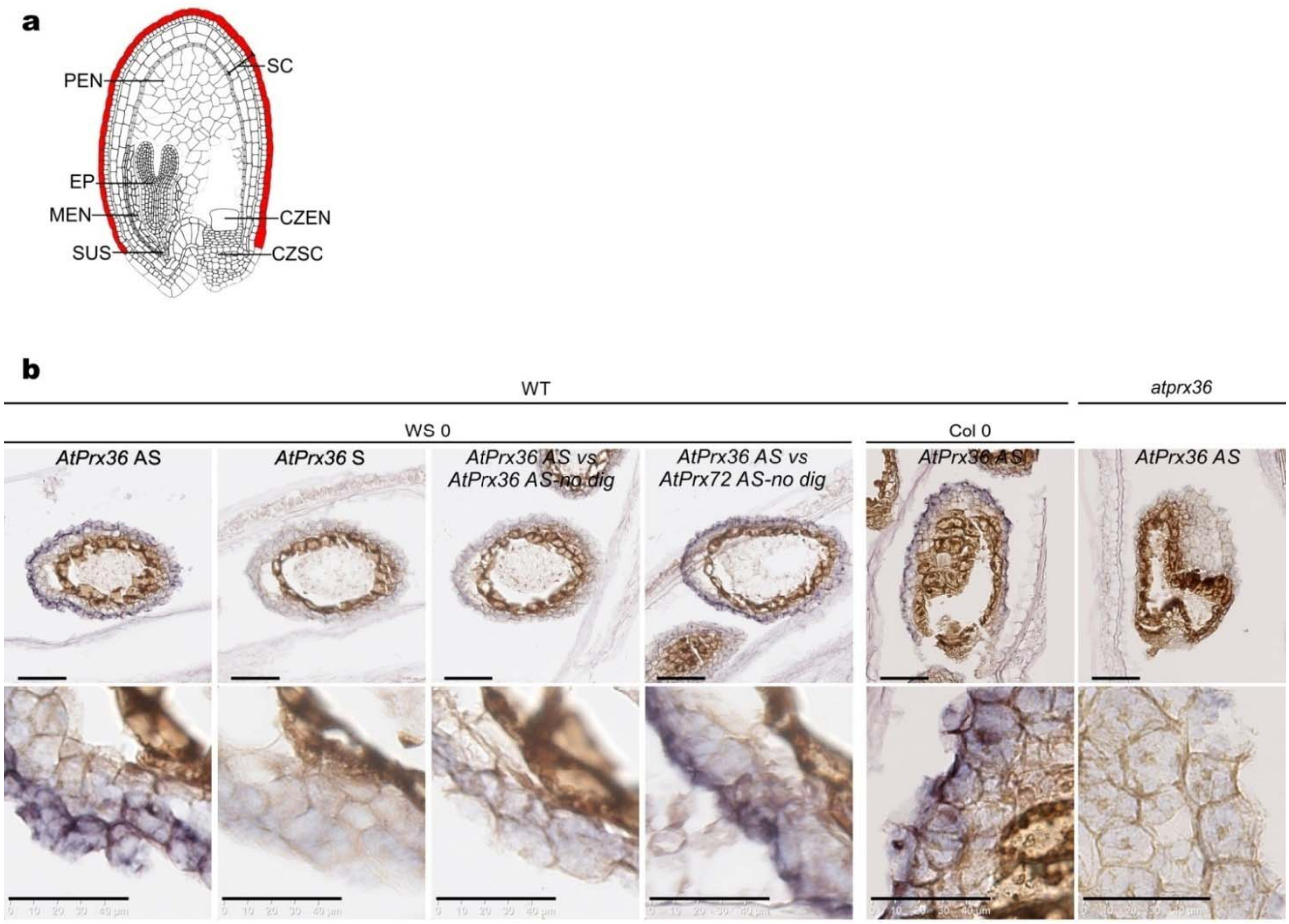

Supplementary Figure S45: Illustration of the specificity of *in situ* hybridization (ISH) signals using competitive inhibition and knock out (KO) mutant line.

(a) Cell-specific ISH map for *AtPRX36* redrawn after **Supplementary Figure S12.** **(b)** Serial sections of tissue microarray from wild type (WT) Ws ecotype was used for hybridization with *AtPRX36* AS or S probes as previously performed but also for competitive inhibition with co-hybridized *AtPRX36* AS probes with a 10-fold excess of *AtPRX36* unlabelled AS probes (AS-no dig) or of *AtPRX72* unlabelled AS probes (AS-no dig). Note that *AtPRX72* is the second best hit for *AtPRX36* among all cDNAs with 82.2% nucleotide identity (**Supplementary Table S8**). The top line images show serial section from the same whole seed and the bottom line images show magnifications. Note the specific ISH signal detected with *AtPRX36* AS probes and *AtPRX36* AS probe pre-incubated with *AtPRX72* unlabelled probes and the absence of signals with the negative controls (*AtPRX36* S probe and *AtPRX36* AS probe pre-incubated with *AtPRX36* unlabelled probes. **(c)** ISH results on sections from the same developmental stage from WT (Col-0 ecotype) and KO mutant line for *AtPRX36* generated in Col-0 genetic background (*atprx36*). Note the disappearance of the ISH signal in the KO line, further demonstrating the specificity of hybridization. Scale bars: 100µm (top line images) and 50µm (bottom line images)

Supplementary Figures S46-S51: Thresholds of tissue-specific transcriptomic values compatible with ISH are specific to each developmental stage/tissue.

For each developmental stage (**Supplementary Figure S46,** preglobular stage; **S47,** globular stage**; S48,** heart stage; **S49,** linear cotyledons stage; **S50,** bending cotyledons stage; **S51,** mature green stage) and each tissue (**a,** embryo proper; **b,** micropylar endosperm; **c,** peripheral endosperm; **d,** chalazal endosperm; **e,** chalazal seed coat; **f,** seed coat; **g,** suspensor) used for the tissue-specific transcriptomic sampling, the 23,933 genes present on the microarray were classified according to their transcriptomic expression values. The range 0-44 corresponded to the expression values below the 45 detection limit of the transcriptomic study (more than 50% of the genes in each sample); the range 45-299 corresponded to the maximum expression values between the 45 detection limit of the transcriptomics and the 300 arbitrary cut-off that we initially defined for our ISH study; the other ranges were arbitrary set to allow the distribution of the genes in various expression value groups. The resulting number of genes within each range was plotted on individual graphs.

All the genes analysed by ISH in this study were positioned above the graph according to their individual tissue-specific transcriptomic expression value. The ISH results were colour-coded using red, ISH strong signal; orange, ISH moderate-to-low signal; white, no detected ISH signal, according to **Supplementary Figures S4-S42** and **Supplementary Table S5**. The deducted total number of genes compatible to various extend with ISH was posted on the top of each graph within double arrows using the same colour coding. This clearly illustrates that the sensitivity of ISH is dependant on spatiotemporal parameters and that transcriptomics and ISH are complementary. The scarcity of ISH signal for preglobular stage could be explained by its underrepresentation on the tissue-arrays.

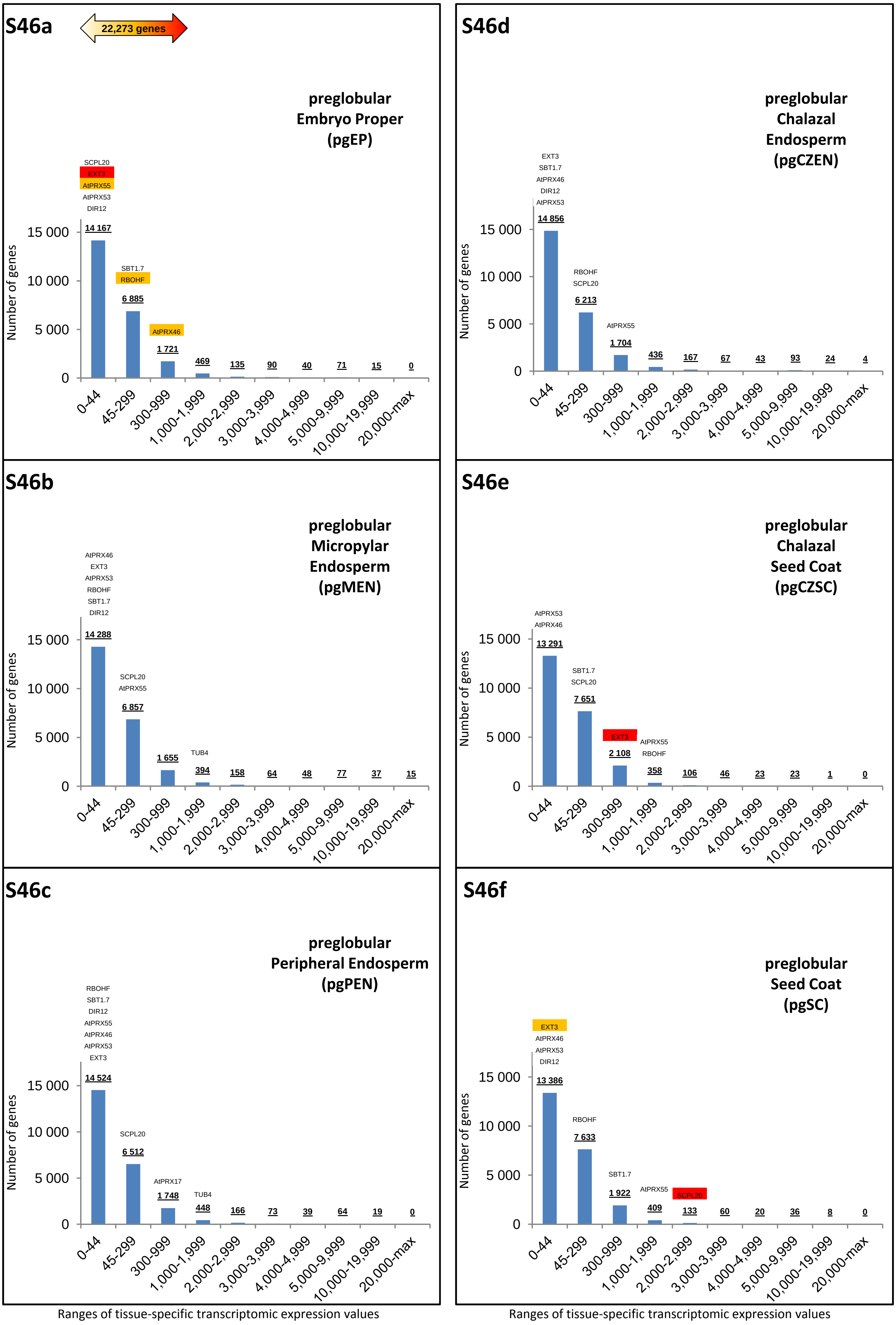
S46a
22,273 genes
preglobular
Embryo Proper
(pgEP)
SCPL20
EXT3
AtPRX55
AtPRX53
DIR12
14 167
SBT1.7
RBOHF
6 885
AtPRX46
1 721
469
135
90
40
71
15
0
S46d
preglobular
Chalazal
Endosperm
(pgCZEN)
EXT3
SBT1.7
AtPRX46
DIR12
AtPRX53
14 856
RBOHF
SCPL20
6 213
AtPRX55
1 704
436
167
67
43
93
24
4
S46b
preglobular
Micropylar
Endosperm
(pgMEN)
AtPRX46
EXT3
AtPRX53
RBOHF
SBT1.7
DIR12
14 288
SCPL20
AtPRX55
6 857
1 655
TUB4
394
158
64
48
77
37
15
S46e
preglobular
Chalazal
Seed Coat
(pgCZSC)
AtPRX53
AtPRX46
13 291
SBT1.7
SCPL20
7 651
EXT3
2 108
AtPRX55
RBOHF
358
106
46
23
23
1
0
S46c
preglobular
Peripheral Endosperm
(pgPEN)
RBOHF
SBT1.7
DIR12
AtPRX55
AtPRX46
AtPRX53
EXT3
14 524
SCPL20
6 512
AtPRX17
1 748
TUB4
448
166
73
39
64
19
0
S46f
preglobular
Seed Coat
(pgSC)
EXT3
AtPRX46
AtPRX53
DIR12
13 386
RBOHF
7 633
SBT1.7
1 922
AtPRX55
409
SCPL20
133
60
20
36
8
0
Number of genes
0
5 000
10 000
15 000
0-44
45-299
300-999
1,000-1,999
2,000-2,999
3,000-3,999
4,000-4,999
5,000-9,999
10,000-19,999
20,000-max
Ranges of tissue-specific transcriptomic expression values
Ranges of tissue-specific transcriptomic expression values

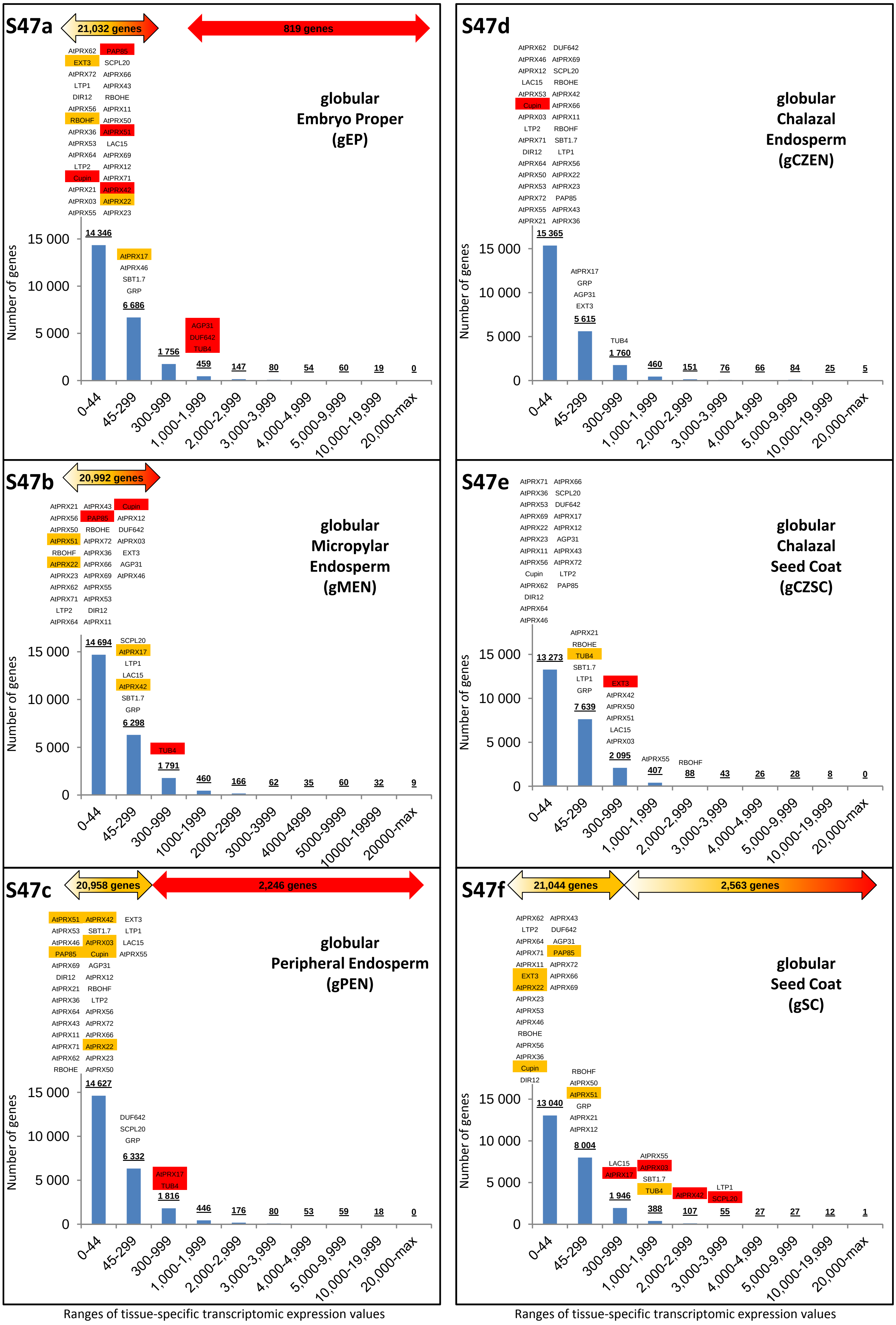
S47a
21,032 genes
819 genes
globular
Embryo Proper
(gEP)
AtPRX62 PAP85
EXT3 SCPL20
AtPRX72 AtPRX66
LTP1 AtPRX43
DIR12 RBOHE
AtPRX56 AtPRX11
RBOHF AtPRX50
AtPRX36 AtPRX51
AtPRX53 LAC15
AtPRX64 AtPRX69
LTP2 AtPRX12
Cupin AtPRX71
AtPRX21 AtPRX42
AtPRX03 AtPRX22
AtPRX55 AtPRX23
AtPRX17
AtPRX46
SBT1.7
GRP
AGP31
DUF642
TUB4
14 346
6 686
1 756
459
147
80
54
60
19
0
Number of genes
15 000
10 000
5 000
0
0-44
45-299
300-999
1,000-1,999
2,000-2,999
3,000-3,999
4,000-4,999
5,000-9,999
10,000-19,999
20,000-max
S47b
20,992 genes
globular
Micropylar
Endosperm
(gMEN)
AtPRX21 AtPRX43 Cupin
AtPRX56 PAP85 AtPRX12
AtPRX50 RBOHE DUF642
AtPRX51 AtPRX72 AtPRX03
RBOHF AtPRX36 EXT3
AtPRX22 AtPRX66 AGP31
AtPRX23 AtPRX69 AtPRX46
AtPRX62 AtPRX55
AtPRX71 AtPRX53
LTP2 DIR12
AtPRX64 AtPRX11
SCPL20
AtPRX17
LTP1
LAC15
AtPRX42
SBT1.7
GRP
TUB4
14 694
6 298
1 791
460
166
62
35
60
32
9
Number of genes
15 000
10 000
5 000
0
0-44
45-299
300-999
1000-1999
2000-2999
3000-3999
4000-4999
5000-9999
10000-19999
20000-max
S47c
20,958 genes
2,246 genes
globular
Peripheral Endosperm
(gPEN)
AtPRX51 AtPRX42 EXT3
AtPRX53 SBT1.7 LTP1
AtPRX46 AtPRX03 LAC15
PAP85 Cupin AtPRX55
AtPRX69 AGP31
DIR12 AtPRX12
AtPRX21 RBOHF
AtPRX36 LTP2
AtPRX64 AtPRX56
AtPRX43 AtPRX72
AtPRX11 AtPRX66
AtPRX71 AtPRX22
AtPRX62 AtPRX23
RBOHE AtPRX50
DUF642
SCPL20
GRP
AtPRX17
TUB4
14 627
6 332
1 816
446
176
80
53
59
18
0
Number of genes
15 000
10 000
5 000
0
0-44
45-299
300-999
1,000-1,999
2,000-2,999
3,000-3,999
4,000-4,999
5,000-9,999
10,000-19,999
20,000-max
Ranges of tissue-specific transcriptomic expression values
S47d
globular
Chalazal
Endosperm
(gCZEN)
AtPRX62 DUF642
AtPRX46 AtPRX69
AtPRX12 SCPL20
LAC15 RBOHE
AtPRX53 AtPRX42
Cupin AtPRX66
AtPRX03 AtPRX11
LTP2 RBOHF
AtPRX71 SBT1.7
DIR12 LTP1
AtPRX64 AtPRX56
AtPRX50 AtPRX22
AtPRX53 AtPRX23
AtPRX72 PAP85
AtPRX55 AtPRX43
AtPRX21 AtPRX36
AtPRX17
GRP
AGP31
EXT3
TUB4
15 365
5 615
1 760
460
151
76
66
84
25
5
Number of genes
15 000
10 000
5 000
0
0-44
45-299
300-999
1,000-1,999
2,000-2,999
3,000-3,999
4,000-4,999
5,000-9,999
10,000-19,999
20,000-max
S47e
globular
Chalazal
Seed Coat
(gCZSC)
AtPRX71 AtPRX66
AtPRX36 SCPL20
AtPRX53 DUF642
AtPRX69 AtPRX17
AtPRX22 AtPRX12
AtPRX23 AGP31
AtPRX11 AtPRX43
AtPRX56 AtPRX72
Cupin LTP2
AtPRX62 PAP85
DIR12
AtPRX64
AtPRX46
AtPRX21
RBOHE
TUB4
SBT1.7
LTP1
GRP
EXT3
AtPRX42
AtPRX50
AtPRX51
LAC15
AtPRX03
AtPRX55
RBOHF
13 273
7 639
2 095
407
88
43
26
28
8
0
Number of genes
15 000
10 000
5 000
0
0-44
45-299
300-999
1,000-1,999
2,000-2,999
3,000-3,999
4,000-4,999
5,000-9,999
10,000-19,999
20,000-max
S47f
21,044 genes
2,563 genes
globular
Seed Coat
(gSC)
AtPRX62 AtPRX43
LTP2 DUF642
AtPRX64 AGP31
AtPRX71 PAP85
AtPRX11 AtPRX72
EXT3 AtPRX66
AtPRX22 AtPRX69
AtPRX23
AtPRX53
AtPRX46
RBOHE
AtPRX56
AtPRX36
Cupin
DIR12
RBOHF
AtPRX50
AtPRX51
GRP
AtPRX21
AtPRX12
LAC15
AtPRX17
AtPRX55
AtPRX03
SBT1.7
TUB4
AtPRX42
LTP1
SCPL20
13 040
8 004
1 946
388
107
55
27
27
12
1
Number of genes
15 000
10 000
5 000
0
0-44
45-299
300-999
1,000-1,999
2,000-2,999
3,000-3,999
4,000-4,999
5,000-9,999
10,000-19,999
20,000-max
Ranges of tissue-specific transcriptomic expression values

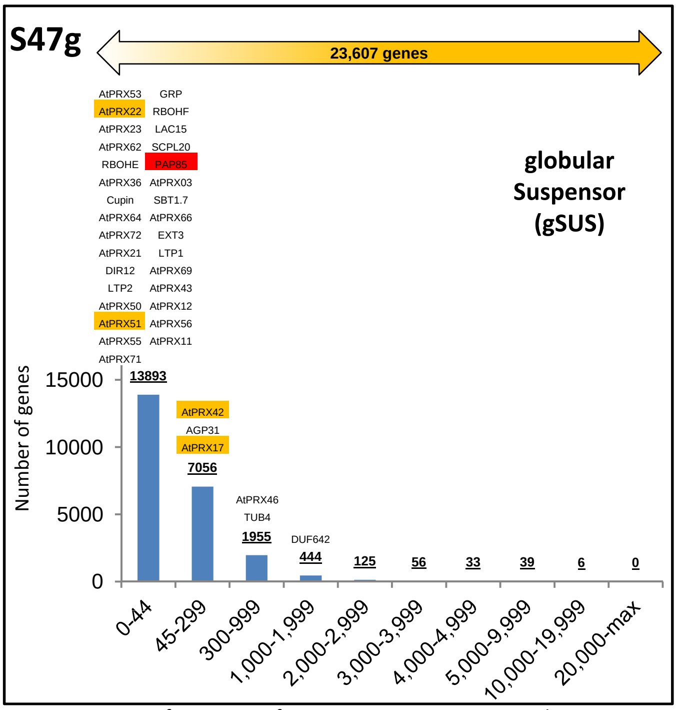

Ranges of tissue-specific transcriptomic expression values

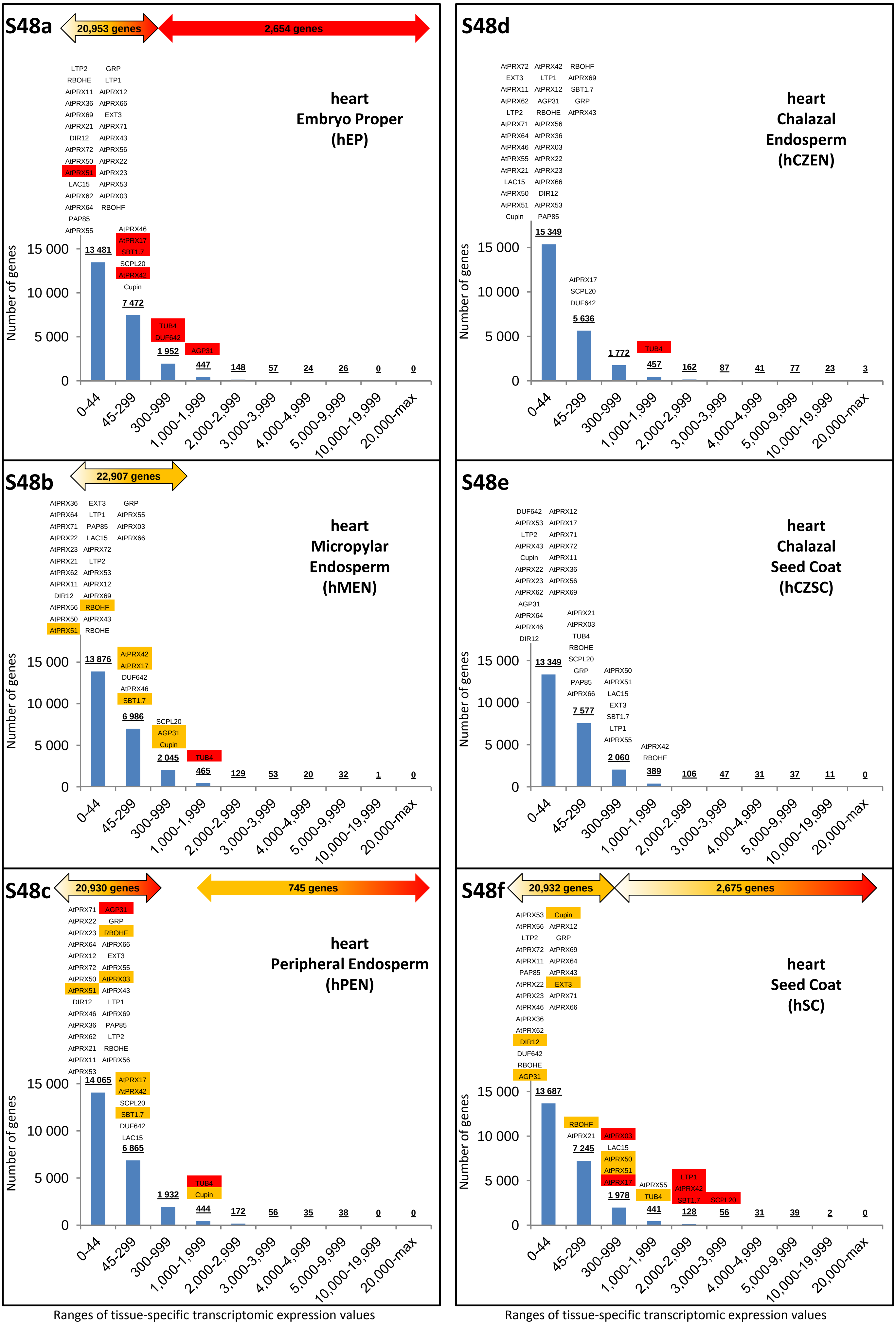
S48a
20,953 genes
2,654 genes
heart
Embryo Proper
(hEP)
LTP2 GRP
RBOHE LTP1
AtPRX11 AtPRX12
AtPRX36 AtPRX66
AtPRX69 EXT3
AtPRX21 AtPRX71
DIR12 AtPRX43
AtPRX72 AtPRX56
AtPRX50 AtPRX22
AtPRX51 AtPRX23
LAC15 AtPRX53
AtPRX62 AtPRX03
AtPRX64 RBOHF
PAP85
AtPRX55
AtPRX46
AtPRX17
SBT1.7
SCPL20
AtPRX42
Cupin
TUB4
DUF642
AGP31
13 481
7 472
1 952
447
148
57
24
26
0
0
S48d
heart
Chalazal
Endosperm
(hCZEN)
AtPRX72 AtPRX42 RBOHF
EXT3 LTP1 AtPRX69
AtPRX11 AtPRX12 SBT1.7
AtPRX62 AGP31 GRP
LTP2 RBOHE AtPRX43
AtPRX71 AtPRX56
AtPRX64 AtPRX36
AtPRX46 AtPRX03
AtPRX55 AtPRX22
AtPRX21 AtPRX23
LAC15 AtPRX66
AtPRX50 DIR12
AtPRX51 AtPRX53
Cupin PAP85
AtPRX17
SCPL20
DUF642
TUB4
15 349
5 636
1 772
457
162
87
41
77
23
3
S48b
22,907 genes
heart
Micropylar
Endosperm
(hMEN)
AtPRX36 EXT3 GRP
AtPRX64 LTP1 AtPRX55
AtPRX71 PAP85 AtPRX03
AtPRX22 LAC15 AtPRX66
AtPRX23 AtPRX72
AtPRX21 LTP2
AtPRX62 AtPRX53
AtPRX11 AtPRX12
DIR12 AtPRX69
AtPRX56 RBOHF
AtPRX50 AtPRX43
AtPRX51 RBOHE
AtPRX42
AtPRX17
DUF642
AtPRX46
SBT1.7
SCPL20
AGP31
Cupin
TUB4
13 876
6 986
2 045
465
129
53
20
32
1
0
S48e
heart
Chalazal
Seed Coat
(hCZSC)
DUF642 AtPRX12
AtPRX53 AtPRX17
LTP2 AtPRX71
AtPRX43 AtPRX72
Cupin AtPRX11
AtPRX22 AtPRX36
AtPRX23 AtPRX56
AtPRX62 AtPRX69
AGP31
AtPRX64
AtPRX46
DIR12
AtPRX21
AtPRX03
TUB4
RBOHE
SCPL20
GRP
PAP85
AtPRX66
AtPRX50
AtPRX51
LAC15
EXT3
SBT1.7
LTP1
AtPRX55
AtPRX42
RBOHF
13 349
7 577
2 060
389
106
47
31
37
11
0
S48c
20,930 genes
745 genes
heart
Peripheral Endosperm
(hPEN)
AtPRX71 AGP31
AtPRX22 GRP
AtPRX23 RBOHF
AtPRX64 AtPRX66
AtPRX12 EXT3
AtPRX72 AtPRX55
AtPRX50 AtPRX03
AtPRX51 AtPRX43
DIR12 LTP1
AtPRX46 AtPRX69
AtPRX36 PAP85
AtPRX62 LTP2
AtPRX21 RBOHE
AtPRX11 AtPRX56
AtPRX53
AtPRX17
AtPRX42
SCPL20
SBT1.7
DUF642
LAC15
TUB4
Cupin
14 065
6 865
1 932
444
172
56
35
38
0
0
S48f
20,932 genes
2,675 genes
heart
Seed Coat
(hSC)
AtPRX53 Cupin
AtPRX56 AtPRX12
LTP2 GRP
AtPRX72 AtPRX69
AtPRX11 AtPRX64
PAP85 AtPRX43
AtPRX22 EXT3
AtPRX23 AtPRX71
AtPRX46 AtPRX66
AtPRX36
AtPRX62
DIR12
DUF642
RBOHE
AGP31
RBOHF
AtPRX21
AtPRX03
LAC15
AtPRX50
AtPRX51
AtPRX17
AtPRX55
TUB4
LTP1
AtPRX42
SBT1.7
SCPL20
13 687
7 245
1 978
441
128
56
31
39
2
0
Number of genes
15 000
10 000
5 000
0
0-44
45-299
300-999
1,000-1,999
2,000-2,999
3,000-3,999
4,000-4,999
5,000-9,999
10,000-19,999
20,000-max
Ranges of tissue-specific transcriptomic expression values

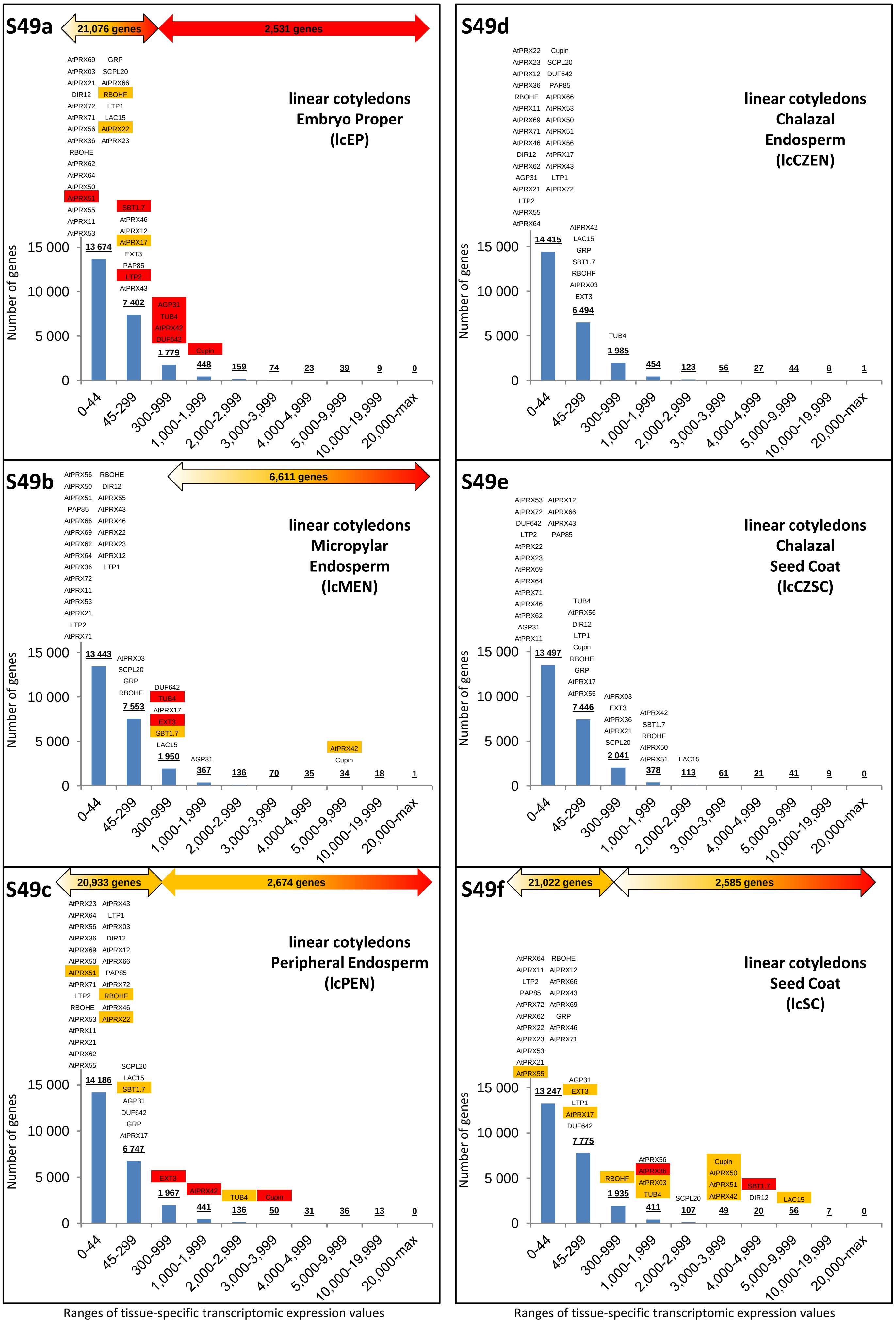

S49a
21,076 genes
2,531 genes
linear cotyledons Embryo Proper (lcEP)
AtPRX69 GRP
AtPRX03 SCPL20
AtPRX21 AtPRX66
DIR12 RBOHF
AtPRX72 LTP1
AtPRX71 LAC15
AtPRX56 AtPRX22
AtPRX36 AtPRX23
RBOHE
AtPRX62
AtPRX64
AtPRX50
AtPRX51
AtPRX55
AtPRX11
AtPRX53
SBT1.7
AtPRX46
AtPRX12
AtPRX17
EXT3
PAP85
LTP2
AtPRX43
AGP31
TUB4
AtPRX42
DUF642
Cupin
13 674
7 402
1 779
448
159
74
23
39
9
0
S49b
6,611 genes
linear cotyledons Micropylar Endosperm (lcMEN)
AtPRX56 RBOHE
AtPRX50 DIR12
AtPRX51 AtPRX55
PAP85 AtPRX43
AtPRX66 AtPRX46
AtPRX69 AtPRX22
AtPRX62 AtPRX23
AtPRX64 AtPRX12
AtPRX36 LTP1
AtPRX72
AtPRX11
AtPRX53
AtPRX21
LTP2
AtPRX71
AtPRX03
SCPL20
GRP
RBOHF
DUF642
TUB4
AtPRX17
EXT3
SBT1.7
LAC15
AGP31
AtPRX42
Cupin
13 443
7 553
1 950
367
136
70
35
34
18
1
S49c
20,933 genes
2,674 genes
linear cotyledons Peripheral Endosperm (lcPEN)
AtPRX23 AtPRX43
AtPRX64 LTP1
AtPRX56 AtPRX03
AtPRX36 DIR12
AtPRX69 AtPRX12
AtPRX50 AtPRX66
AtPRX51 PAP85
AtPRX71 AtPRX72
LTP2 RBOHF
RBOHE AtPRX46
AtPRX53 AtPRX22
AtPRX11
AtPRX21
AtPRX62
AtPRX55
SCPL20
LAC15
SBT1.7
AGP31
DUF642
GRP
AtPRX17
EXT3
AtPRX42
TUB4
Cupin
14 186
6 747
1 967
441
136
50
31
36
13
0
S49d
linear cotyledons Chalazal Endosperm (lcCZEN)
AtPRX22 Cupin
AtPRX23 SCPL20
AtPRX12 DUF642
AtPRX36 PAP85
RBOHE AtPRX66
AtPRX11 AtPRX53
AtPRX69 AtPRX50
AtPRX71 AtPRX51
AtPRX46 AtPRX56
DIR12 AtPRX17
AtPRX62 AtPRX43
AGP31 LTP1
AtPRX21 AtPRX72
LTP2
AtPRX55
AtPRX64
AtPRX42
LAC15
GRP
SBT1.7
RBOHF
AtPRX03
EXT3
TUB4
14 415
6 494
1 985
454
123
56
27
44
8
1
S49e
linear cotyledons Chalazal Seed Coat (lcCZSC)
AtPRX53 AtPRX12
AtPRX72 AtPRX66
DUF642 AtPRX43
LTP2 PAP85
AtPRX22
AtPRX23
AtPRX69
AtPRX64
AtPRX71
AtPRX46
AtPRX62
AGP31
AtPRX11
TUB4
AtPRX56
DIR12
LTP1
Cupin
RBOHE
GRP
AtPRX17
AtPRX55
AtPRX03
EXT3
AtPRX36
AtPRX21
SCPL20
AtPRX42
SBT1.7
RBOHF
AtPRX50
AtPRX51
LAC15
13 497
7 446
2 041
378
113
61
21
41
9
0
S49f
21,022 genes
2,585 genes
linear cotyledons Seed Coat (lcSC)
AtPRX64 RBOHE
AtPRX11 AtPRX12
LTP2 AtPRX66
PAP85 AtPRX43
AtPRX72 AtPRX69
AtPRX62 GRP
AtPRX22 AtPRX46
AtPRX23 AtPRX71
AtPRX53
AtPRX21
AtPRX55
AGP31
EXT3
LTP1
AtPRX17
DUF642
RBOHF
AtPRX56
AtPRX36
AtPRX03
TUB4
SCPL20
Cupin
AtPRX50
AtPRX51
AtPRX42
SBT1.7
DIR12
LAC15
13 247
7 775
1 935
411
107
49
20
56
7
0
Number of genes
0
5 000
10 000
15 000
0-44
45-299
300-999
1,000-1,999
2,000-2,999
3,000-3,999
4,000-4,999
5,000-9,999
10,000-19,999
20,000-max
Ranges of tissue-specific transcriptomic expression values

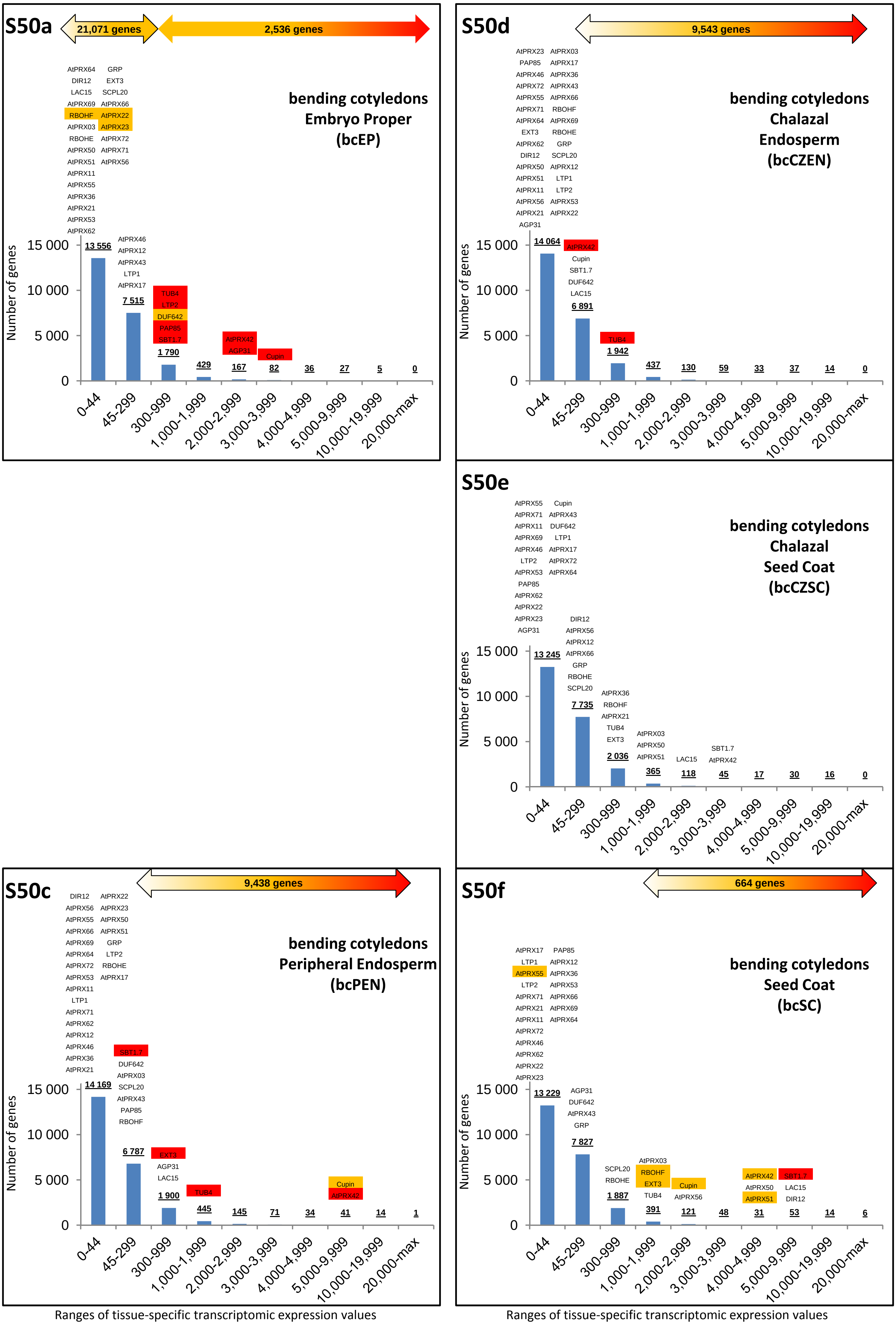
S50a
21,071 genes
2,536 genes
bending cotyledons
Embryo Proper
(bcEP)
AtPRX64 GRP
DIR12 EXT3
LAC15 SCPL20
AtPRX69 AtPRX66
RBOHF AtPRX22
AtPRX03 AtPRX23
RBOHE AtPRX72
AtPRX50 AtPRX71
AtPRX51 AtPRX56
AtPRX11
AtPRX55
AtPRX36
AtPRX21
AtPRX53
AtPRX62
13 556
AtPRX46
AtPRX12
AtPRX43
LTP1
AtPRX17
7 515
TUB4
LTP2
DUF642
PAP85
SBT1.7
1 790
429
AtPRX42
AGP31
167
Cupin
82
36
27
5
0
S50d
9,543 genes
bending cotyledons
Chalazal
Endosperm
(bcCZEN)
AtPRX23 AtPRX03
PAP85 AtPRX17
AtPRX46 AtPRX36
AtPRX72 AtPRX43
AtPRX55 AtPRX66
AtPRX71 RBOHF
AtPRX64 AtPRX69
EXT3 RBOHE
AtPRX62 GRP
DIR12 SCPL20
AtPRX50 AtPRX12
AtPRX51 LTP1
AtPRX11 LTP2
AtPRX56 AtPRX53
AtPRX21 AtPRX22
AGP31
14 064
AtPRX42
Cupin
SBT1.7
DUF642
LAC15
6 891
TUB4
1 942
437
130
59
33
37
14
0
S50e
bending cotyledons
Chalazal
Seed Coat
(bcCZSC)
AtPRX55 Cupin
AtPRX71 AtPRX43
AtPRX11 DUF642
AtPRX69 LTP1
AtPRX46 AtPRX17
LTP2 AtPRX72
AtPRX53 AtPRX64
PAP85
AtPRX62
AtPRX22
AtPRX23
AGP31
13 245
DIR12
AtPRX56
AtPRX12
AtPRX66
GRP
RBOHE
SCPL20
7 735
AtPRX36
RBOHF
AtPRX21
TUB4
EXT3
2 036
AtPRX03
AtPRX50
AtPRX51
365
LAC15
118
SBT1.7
AtPRX42
45
17
30
16
0
S50c
9,438 genes
bending cotyledons
Peripheral Endosperm
(bcPEN)
DIR12 AtPRX22
AtPRX56 AtPRX23
AtPRX55 AtPRX50
AtPRX66 AtPRX51
AtPRX69 GRP
AtPRX64 LTP2
AtPRX72 RBOHE
AtPRX53 AtPRX17
AtPRX11
LTP1
AtPRX71
AtPRX62
AtPRX12
AtPRX46
AtPRX36
AtPRX21
14 169
SBT1.7
DUF642
AtPRX03
SCPL20
AtPRX43
PAP85
RBOHF
6 787
EXT3
AGP31
LAC15
1 900
TUB4
445
145
71
34
Cupin
AtPRX42
41
14
1
S50f
664 genes
bending cotyledons
Seed Coat
(bcSC)
AtPRX17 PAP85
LTP1 AtPRX12
AtPRX55 AtPRX36
LTP2 AtPRX53
AtPRX71 AtPRX66
AtPRX21 AtPRX69
AtPRX11 AtPRX64
AtPRX72
AtPRX46
AtPRX62
AtPRX22
AtPRX23
13 229
AGP31
DUF642
AtPRX43
GRP
7 827
SCPL20
RBOHE
1 887
AtPRX03
RBOHF
EXT3
TUB4
391
Cupin
AtPRX56
121
48
AtPRX42
AtPRX50
AtPRX51
31
SBT1.7
LAC15
DIR12
53
14
6
Number of genes
15 000
10 000
5 000
0
0-44
45-299
300-999
1,000-1,999
2,000-2,999
3,000-3,999
4,000-4,999
5,000-9,999
10,000-19,999
20,000-max
Ranges of tissue-specific transcriptomic expression values

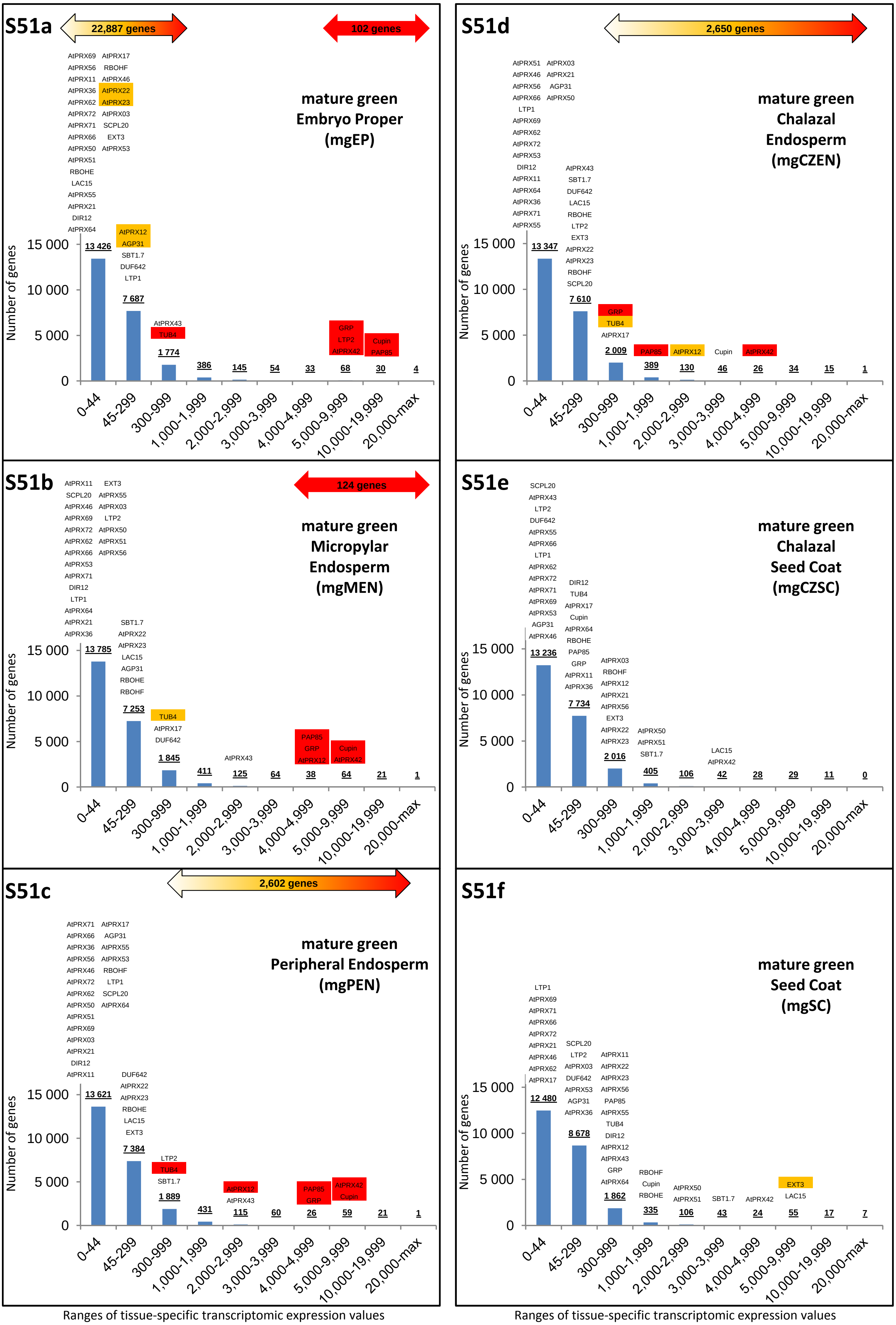
S51a
22,887 genes
102 genes
mature green
Embryo Proper
(mgEP)
AtPRX69 AtPRX17
AtPRX56 RBOHF
AtPRX11 AtPRX46
AtPRX36 AtPRX22
AtPRX62 AtPRX23
AtPRX72 AtPRX03
AtPRX71 SCPL20
AtPRX66 EXT3
AtPRX50 AtPRX53
AtPRX51
RBOHE
LAC15
AtPRX55
AtPRX21
DIR12
AtPRX64
AtPRX12
AGP31
SBT1.7
DUF642
LTP1
AtPRX43
TUB4
GRP
LTP2
AtPRX42
Cupin
PAP85
13 426
7 687
1 774
386
145
54
33
68
30
4
Number of genes
15 000
10 000
5 000
0
0-44
45-299
300-999
1,000-1,999
2,000-2,999
3,000-3,999
4,000-4,999
5,000-9,999
10,000-19,999
20,000-max
S51d
2,650 genes
mature green
Chalazal
Endosperm
(mgCZEN)
AtPRX51 AtPRX03
AtPRX46 AtPRX21
AtPRX56 AGP31
AtPRX66 AtPRX50
LTP1
AtPRX69
AtPRX62
AtPRX72
AtPRX53
DIR12
AtPRX11
AtPRX64
AtPRX36
AtPRX71
AtPRX55
AtPRX43
SBT1.7
DUF642
LAC15
RBOHE
LTP2
EXT3
AtPRX22
AtPRX23
RBOHF
SCPL20
GRP
TUB4
AtPRX17
PAP85
AtPRX12
Cupin
AtPRX42
13 347
7 610
2 009
389
130
46
26
34
15
1
Number of genes
15 000
10 000
5 000
0
0-44
45-299
300-999
1,000-1,999
2,000-2,999
3,000-3,999
4,000-4,999
5,000-9,999
10,000-19,999
20,000-max
S51b
124 genes
mature green
Micropylar
Endosperm
(mgMEN)
AtPRX11 EXT3
SCPL20 AtPRX55
AtPRX46 AtPRX03
AtPRX69 LTP2
AtPRX72 AtPRX50
AtPRX62 AtPRX51
AtPRX66 AtPRX56
AtPRX53
AtPRX71
DIR12
LTP1
AtPRX64
AtPRX21
AtPRX36
SBT1.7
AtPRX22
AtPRX23
LAC15
AGP31
RBOHE
RBOHF
TUB4
AtPRX17
DUF642
AtPRX43
PAP85
GRP
AtPRX12
Cupin
AtPRX42
13 785
7 253
1 845
411
125
64
38
64
21
1
Number of genes
15 000
10 000
5 000
0
0-44
45-299
300-999
1,000-1,999
2,000-2,999
3,000-3,999
4,000-4,999
5,000-9,999
10,000-19,999
20,000-max
S51e
mature green
Chalazal
Seed Coat
(mgCZSC)
SCPL20
AtPRX43
LTP2
DUF642
AtPRX55
AtPRX66
LTP1
AtPRX62
AtPRX72
AtPRX71
AtPRX69
AtPRX53
AGP31
AtPRX46
DIR12
TUB4
AtPRX17
Cupin
AtPRX64
RBOHE
PAP85
GRP
AtPRX11
AtPRX36
AtPRX03
RBOHF
AtPRX12
AtPRX21
AtPRX56
EXT3
AtPRX22
AtPRX23
AtPRX50
AtPRX51
SBT1.7
LAC15
AtPRX42
13 236
7 734
2 016
405
106
42
28
29
11
0
Number of genes
15 000
10 000
5 000
0
0-44
45-299
300-999
1,000-1,999
2,000-2,999
3,000-3,999
4,000-4,999
5,000-9,999
10,000-19,999
20,000-max
S51c
2,602 genes
mature green
Peripheral Endosperm
(mgPEN)
AtPRX71 AtPRX17
AtPRX66 AGP31
AtPRX36 AtPRX55
AtPRX56 AtPRX53
AtPRX46 RBOHF
AtPRX72 LTP1
AtPRX62 SCPL20
AtPRX50 AtPRX64
AtPRX51
AtPRX69
AtPRX03
AtPRX21
DIR12
AtPRX11
DUF642
AtPRX22
AtPRX23
RBOHE
LAC15
EXT3
LTP2
TUB4
SBT1.7
AtPRX12
AtPRX43
PAP85
GRP
AtPRX42
Cupin
13 621
7 384
1 889
431
115
60
26
59
21
1
Number of genes
15 000
10 000
5 000
0
0-44
45-299
300-999
1,000-1,999
2,000-2,999
3,000-3,999
4,000-4,999
5,000-9,999
10,000-19,999
20,000-max
S51f
mature green
Seed Coat
(mgSC)
LTP1
AtPRX69
AtPRX71
AtPRX66
AtPRX72
AtPRX21
AtPRX46
AtPRX62
AtPRX17
SCPL20
LTP2
AtPRX03
DUF642
AtPRX53
AGP31
AtPRX36
AtPRX11
AtPRX22
AtPRX23
AtPRX56
PAP85
AtPRX55
TUB4
DIR12
AtPRX12
AtPRX43
GRP
AtPRX64
RBOHF
Cupin
RBOHE
AtPRX50
AtPRX51
SBT1.7
AtPRX42
EXT3
LAC15
12 480
8 678
1 862
335
106
43
24
55
17
7
Number of genes
15 000
10 000
5 000
0
0-44
45-299
300-999
1,000-1,999
2,000-2,999
3,000-3,999
4,000-4,999
5,000-9,999
10,000-19,999
20,000-max
Ranges of tissue-specific transcriptomic expression values
Ranges of tissue-specific transcriptomic expression values

Supplementary Methods

We took advantage of a publicly available tissue-specific transcriptomic dataset targeted on the seed development of the model plant Arabidopsis (Belmonte *et al.,* 2013) to develop a simplified and optimized medium throughput non isotopic RNA ISH protocol. This protocol was applied on serial sections of paraffin-embedded samples from Arabidopsis seed development kinetics. As a case study, we chose to screen first the expression profile of a medium size multigenic family: the Arabidopsis class III peroxidases (*AtPRX*). This family consists in 73 genes presenting high nucleotide-sequence identity and various expression levels (Francoz *et al.,* 2015), and therefore constitutes a good model to study both ISH specificity and sensitivity. First, 16 *AtPRX* candidates were selected among highly expressed genes according to transcriptomic data. In a second time, we enlarged our selection list to 6 less abundantly expressed *AtPRX* genes, to 2 *AtPRX*s not spotted on the array, and to 15 additional non-peroxidase genes, reaching the number of 39 candidate genes.

The overall ISH flowchart of our method (**Figure 1**) and the detailed step-by-step **Supplementary Methods** show how we have been able to reach the medium throughput necessary to study dozens of genes with an optimized and simplified traditional non isotopic ISH protocol. Briefly, the digoxigenin-labelled riboprobe synthesis was facilitated by the availability at the RIKEN genomic resource of ready-to-use cDNA containing plasmids for the majority of our genes [http://www.brc.riken.jp/lab/epd/catalog/cdnaclone.html; (Seki *et al.,* 1998, Seki *et al.,* 2002)]. One advantage of riboprobes is their stability for years pending proper storage at -20°C in RNase free conditions, enabling to constitute riboprobe stocks prior the ISH itself. A second parameter allowing to reach medium throughput was the production of concentrated biological samples constituting the tissue arrays inspired by strategies previously developed in the human pathology field (Simon *et al.,* 2004). These consisted in covering the whole kinetics of seed development used for the tissue-specific transcriptomic (**Supplementary Figure S1a**) by concentrating fruit (silique) samples containing numerous seeds within single paraffin-embedded blocks that can be stored for years at 4°C, allowing to constitute stocks of ready-to-use biological sample (**Supplementary Figure S1b**). With in hand stocks of stable riboprobes and tissue-arrays and of all buffers and aliquoted chemicals (detailed in the following sections of **Supplementary Methods**), a typical ISH experiments proceeded as follows. Serial sections of tissue arrays, comprising up to 1,000 developing seeds of various age, were individually spread on 40 microscopy slides, each one being devoted to one riboprobe. Therefore, in a single 3-days ISH experiment, 20 genes could be studied along all developmental kinetics, under high stringency conditions with no particular expensive robot. One half of the 40 slides were used for the antisense (AS) riboprobes and the other half for the corresponding sense (S) riboprobes used as negative controls. The basis of the ISH protocol itself comes from (Jackson 1992) with first adaptations by (St-Pierre *et al.,* 1999, Burlat *et al.,* 2004, Mahroug *et al.,* 2006) and final simplification and full description detailed in the following sections of **Supplementary Methods**. Finally, even if conventional bright field microscopy is suitable for such a project, the medium throughput level was also achieved by taking advantage of slide scanners that allowed to acquire high resolution images collections of the

full slide area, and the analysis of the results was facilitated by the use of an easy-to-use image viewer (**Figure 1; Supplementary Figure S1**).

The paragraph numbering in the **Supplementary Methods** correspond to the flow chart of the method displayed in **Figure 1**.

I. Tissue-specific transcriptomic data mining to select candidate genes for medium-throughput ISH

I.1. Definition of the *AtPRX* toolbox

We used the "microarray element search and download tool" form TAIR (https://www.arabidopsis.org/tools/bulk/microarray/index.jsp) to find Affymetrix ATH1 (25K) spot identifiers corresponding to the 73 *AtPRXs* locus identifiers gathered in the PeroxiBase (Koua *et al.,* 2009, Fawal *et al.,* 2013) (http://peroxibase.toulouse.inra.fr/). This allowed building the *AtPRX* toolbox (**Supplementary Table S2**). Only *AtPRX13* (At1g77100) and *AtPRX32* (At3g32980) were not available since no spot IDs existed for these genes. 6 spot IDs corresponded to 11 ambiguous genes and one pseudogene (*AtPRX01/AtPRX02, AtPRX14/AtPRX15, AtPRX22/AtPRX23, AtPRX29/AtPRX[P]29* (pseudogene)*, AtPRX33/AtPRX34, AtPRX50/AtPRX51*).

I.2. Retrieval of tissue-specific transcriptomic data

Among the publically available tissue-specific transcriptomic datasets, we decided to focus our study on the Harada-Goldberg Arabidopsis seed LCM gene-chip dataset available through different sources [Seedgenenetwork at http://estdb.biology.ucla.edu/ seed/, Gene Expression Omnibus accession series GSE12404 at www.ncbi.nlm.nih.gov/geo, seed eFP browser at http://bar.utoronto.ca/efp/cgi-bin/efpWeb.cgi?dataSource=Seed (Winter *et al.,* 2007), and the original publication (Belmonte *et al.,* 2013)]. This dataset had the advantage to provide up to 42 valuable tissue samples along up to 6 developmental stages of seeds from *A. thaliana* Ws-0 grown under continuous light (**Supplementary Figure S1**). The last three sources have been deeply analysed:

I.2.1. Seed eFP browser

The table of expression values was individually downloaded for the 71 *AtPRXs* available out of the 73 members of the multigenic family. The eFP browser presented identical results for the 6 ambiguous gene pairs identified in the *AtPRX* toolbox.

I.2.2. Original published supplemental data 2 (Belmonte *et al.,* 2013)

Expression profiles from all available *AtPRXs* were extracted from the Supplemental data 2 Microsoft Excel file provided by Belmonte *et al.* (2013) using the *AtPRX* toolbox as query. It has to be noticed that the 12 ambiguous genes (6 gene pairs) were not found when using locus identifiers (AGI) as

query whereas the 71 genes from the *AtPRX* toolbox were found when using the ATH1 probe set IDs as query.

I.2.3. Gene Expression Omnibus accession series GSE12404

Gene Expression Omnibus accession series GSE12404 was downloaded as series matrix files at www.ncbi.nlm.nih.gov/geo. The ".txt" file was converted to ".xls" file which was further annotated and edited (**Supplementary Table S1**). Briefly, the 87 individual GSM samples corresponded to 39 samples with duplicate values and 3 samples with triplicate values. The resulting 42 mean data series were classified in 6-8 tissues along 6 developmental stages and "normalized" following the same scheme used in the seed eFP browser (http://bar.utoronto.ca/efp/cgi-bin/efpWeb.cgi?dataSource=Seed). Replicates values were added and divided by 10 and triplicate values (3 samples highlighted in beige) were added, multiplied by 2/3 and divided by 10 (**Supplementary Table S1**). The original series matrix file only contained the probe set IDs as an identification mean. We used the "microarray element search and download tool" from TAIR (https://www.arabidopsis.org/tools/bulk/microarray/index.jsp) to find locus identifiers, gene symbols and annotations corresponding to the 22,810 Affymetrix ATH1 (25K) probe set IDs. 21,770 were considered as non ambiguous spot IDs (corresponding to a unique locus identifier), and 1,040 as ambiguous spot IDs corresponding all together to 2,415 genes (**Supplementary Table S1**). The locus identifiers and gene symbol corresponding to the 22,810 spot IDs were annotated in the GSE12404 series matrix file using this resource. Finally, the maximum expression value was determined for each spot ID and the whole table was classified and ranked following decreasing maximum expression value for each spot ID and a three-colour coded heat map was drawn in Microsoft Excel using grey (minimal value)/yellow (arbitrary cut-off= 300)/red (maximal value) (**Supplementary Table S1**).

I.3. Unification of tissue-specific transcriptomic data

The expression profiles of the 16 most expressed *AtPRXs* retrieved from these three resources were collected in a single Microsoft Excel file. Pearson correlation coefficients (PCCs) were calculated for Belmonte et al *vs.* eFP browser and eFP browser *vs.* GSE 12404, respectively, using the expression data series above the threshold determined by Belmonte *et al.* (2013) (**Supplementary Table S3**). This Table shows that despite different data treatments and different coverage of the samples, the three resources corresponded to a unique original dataset. The eFP browser seed data series referred to (Le *et al.,* 2010) but the data actually corresponded both to the GSE12404 series matrix files and to (Belmonte *et al.,* 2013). The *circa* 3 ratio of signal intensities between the GSE12404/eFP browser datasets and the Belmonte *et al.* (2013) dataset (**Supplementary Table S3**) allowed to convert the detection limit value of 15 defined in the original publication (Belmonte *et al.,* 2013) to a detection limit value of 45 for the GSE12404/eFP browser datasets. In the following, we used the most complete GSE12404 dataset that we organized in a more accessible and fully annotated version, ranking spot IDs/genes in decreasing order of maximum expression values and also systematically including the locus identifiers and the number of genes corresponding to each spot ID (**Supplementary Table S1**).

I.4. Selection of 39 candidate genes

The expression data of the 73 *AtPRXs* was filtered, keeping the absolute ranking information (**Supplementary Table S2**). More than 50% of the *AtPRX* genes (37 out of 73) had maximum expression values below the transcriptomic detection limit of 45. Having in mind the relative sensitivity of ISH, we arbitrarily set a first cut-off of 300 allowing to select the 16 most highly expressed *AtPRXs*. Interestingly, these included two ambiguous spot IDs corresponding to duplicated genes. Our selection reached the number of 24 *AtPRXs* (about 1/3 of the family) by adding 2 randomly selected *AtPRXs* whose maximum expression value was between 45 and 300, 4 randomly selected *AtPRXs* whose maximum expression value was below 45 as well as the 2 *AtPRXs* for which no spot ID was available (**Supplementary Table S2**). Finally, we added 15 non-peroxidase genes, that corresponded to two peroxidase partners (*RBOHE*, *RBOHF*), one housekeeping gene (*TUB4*), and 12 putative cell wall proteins in which we have particular interest (Irshad *et al.,* 2008, Albenne *et al.,* 2013). We paid attention to select candidates presenting maximum tissue-specific transcriptomic expression values ranging from very high to medium levels (**Supplementary Table S5**).

I.5. Search for second best hits for all cDNAs available in TAIR10

The most recent release of the *Arabidopsis* cDNA sequences dataset (TAIR10) was downloaded from The Arabidopsis Information Resource FTP server (ftp://ftp.arabidopsis.org/home/tair/Sequences/blast_datasets/TAIR10_blastsets/). Each sequence of this dataset was searched locally using Blastall 2.2.24 ["blastn" command with the default parameters (Altschul *et al.,* 1997)] against the TAIR10 cDNA database. The second best hit (defined as the best hit beside the query itself) for each of the 33,602 cDNA sequences of TAIR10 is indicated in **Supplementary Table S6**. The **Supplementary Table S7** corresponds to the extraction from the **Supplementary Table S6** of the second best hit of the 15,866 cDNAs with a nucleotide number above 300. The **Supplementary Table S8** corresponds to the extraction from **Supplementary Table S7** of the second best hit among the 73 *AtPRXs* and the 15 additional non peroxidase genes selected for our *in situ* hybridization study.

II. Plasmid resources and riboprobe *in vitro* transcription

II.1. Plasmids available at RIKEN

The full length cDNA (pda clones) ordered at the RIKEN bioresource center [http://www.brc.riken.jp/lab/epd/catalog/cdnaclone.html; (Seki *et al.,* 1998, Seki *et al.,* 2002)] are listed in the **Supplementary Table S9**. These cDNA clones are provided within pBluescript-derived vector series allowing their direct utilization for *in vitro* transcription of riboprobes since the multiple cloning sites are framed by the T3 and T7 RNA polymerase promoters, respectively (**Supplementary Figure S2**).

II.2. pGEM-T Easy cloning of additional plasmids

Five cDNAs not available at RIKEN were amplified using a pool of retro-transcribed mRNAs from various seed developmental stages, and cloned in pGEM-T Easy (Promega) using the following conditions (**Supplementary Table S10**). These clones are included in the **Supplementary Table S9**, and the multiple cloning site (MCS) including SP6 and T7 RNA polymerase promoter position is shown in **Supplementary Figure S2**.

II.3. Bacterial transformation and plasmid preparation

A 10 pg aliquot of RIKEN plasmid (5 pg.μl^{-1} in 10mM Tris / 1mM EDTA pH 8) was added to 50 µl of competent DH5α *Escherichia coli* (Z-Competent™ *E. coli* Transformation Kit; Zymo Research) on ice. The transformation was performed by stretching out the mix on pre-warmed (37°C) Petri dishes containing Luria-Bertani (LB) medium and 50 µg.ml^{-1} ampicillin, and incubating overnight at 37°C. From these plates, one to three isolated colonies were retrieved and sub cultured on solid and liquid LB medium, both of them containing 50 µg.ml^{-1} of ampicillin. From liquid culture, plasmid extraction (10-15 µg) was performed with the GeneJET™ Plasmid Miniprep Kit (Fermentas). The sequence of the plasmids was checked.

II.4. RNase-free conditions

RNAse-free disposable plastic material was used. Kits, enzymes and solutions were devoted to this type of experiment and stored separately. Diethylpyrocarbonate (DEPC)-treated water was prepared according to (Sambrook *et al.,* 1989). It has to be remembered that autoclaving did not degrade RNases.

II.5. Plasmid linearization

Plasmids (5-7 µg) were linearized with excess (20 units) of single cut 5' overhang or blunt restriction enzymes (Promega, Roche) (**Supplementary Table S9**) for 4 h at 37°C. Restriction enzymes producing 3' protruding ends were avoided to prevent the initiation of transcription of opposite transcription products (Schenborn and Mierendorf 1985). Linearization efficiency was controlled on 1% (w/v) agarose gel electrophoresis in Tris Acetate EDTA (1X TAE) buffer (Sambrook *et al.,* 1989). Fully linearized plasmids were purified using the GeneJET Plasmid Miniprep Kit (Fermentas) following manufacturer's instructions except that the protocol started at the neutralization buffer step and the elution was performed in 50 µl DEPC-treated water.

The digestion occurred either at the 5' or the 3' end of the cDNA, in order to produce the template for *in vitro* transcription of the antisense or the sense probes, respectively. Whenever possible, the linearization was performed using enzymes single-cutting in the multiple cloning site (MCS) and not cutting the cDNA (**Supplementary Figure S2**). In some cases, we used enzymes single-cutting at the cDNA ends and not cutting the vector (**Supplementary Figure S3**).

II.6. *In vitro* transcription of riboprobes

In vitro transcription of Digoxigenin (Dig)-labelled or unlabelled riboprobes used for competitive inhibition consisted in:

- 1 µg of linearized plasmid (Vmax=24 µl for Dig-labelled probes and Vmax=18 µl for unlabelled riboprobes)
- 2 µl of Dig RNA labelling mix (10 mM ATP, 10 mM CTP, 10 mM GTP, 6.5 mM UTP, 3.5 mM Dig-UTP, Roche)
or - 8 µl of unlabelled NTP mix (2.5 mM ATP, 2.5 mM CTP, 2.5 mM GTP, 2.5 mM UTP, Promega)
- 8 µl of 5X RNA polymerase transcription buffer (Note that T3 and SP6 RNA polymerase have a common buffer which is different from T7 RNA polymerase buffer, Promega)
- 4 µl of 100 mM DTT (provided with RNA polymerases, Promega)
- 1 µl of T3, T7 or SP6 RNA polymerase (15-17 unit.$µl^{-1}$, Promega) **(Supplementary Table S9)**
- up to 40 µl of DEPC-treated water.

In vitro transcription was performed during 2 h at 37°C, and stopped by 1 µl of 0.5M EDTA (pH 8, 4°C). Then, probes were sequentially precipitated with 6 µl 3M NaOAC pH 5.2 and 140 µl 100% ethanol during 1 h on ice (both solutions were stored at 4°C). After centrifugation at 14,000 × g during 15 min, the pellet was gently washed with 1 ml of 70% ethanol, centrifuged again during 15 min, carefully dried at 45°C and re-suspended by pipetting and vortexing in 50 µl of DEPC-treated water. The resulting transcription product consisted essentially in the remaining linearized plasmid and the produced dig-labelled riboprobe was subsequently referred as "(ribo)probe".

II.7. Riboprobe alkaline hydrolysis and quantification

Before probe hydrolysis, an aliquot of 2 µl of non-hydrolysed transcription product was reserved for further *in vitro* transcription control by agarose gel electrophoresis. 48 µl of remaining probes were hydrolysed by adding 1 volume of 100 mM sodium carbonate buffer pH 10.2, at 60°C for 9-20 min, depending on the transcript size, in order to obtain fragments of about 400 bases (**Supplementary Table S9**). The probe hydrolysis allows a better accessibility to their target while retaining specificity. The hydrolysis duration expressed in min was determined by the formula: $t = (Li - Lf) / (K \times Li \times Lf)$, where Li is initial RNA length (kb), Lf is final RNA length (0.4 kb) and K is a constant 0.11 $kb.min^{-1}$ (Jackson 1992). Control of non-hydrolysed probe quality and quantification of hydrolysed probe was performed by a 1% (w/v) agarose gel electrophoresis in TAE 1X and then by a comparison of fluorescence intensity between RNA probe smear with a quantitative RNA ladder (RiboRuler Low Range RNA Ladder, Fermentas for initial quantification, and then homemade hydrolysed unlabelled riboprobe aliquoted following quantification as compared to the quantitative commercial RNA ladder). In order to make accurate comparison, we used a homemade lightly coloured RNA loading dye to not impair the quantification of the probe smear. This relative riboprobe concentration comparison was performed with the ImageJ software (Schneider *et al.,* 2012) using the "gel plot lanes" option taking

care to exclude the plasmid band from the probe smear selection zone. Calculation to obtain the absolute concentration ($ng.\mu l^{-1}$) was made in Microsoft Excel comparing the peak area. In order to normalise the volume of probe to use per slide, probe stock standard concentration at 25 $ng.kb^{-1}.\mu l^{-1}$ was obtained by dilution of probes in DEPC-treated water according to each initial probe length. This allowed to take into consideration the initial probe length (before hydrolysis) and to normalise the probe concentration according to the number of probe molecules and not only to the weight of probes. Typically, a riboprobe whose initial size was 1 kb had to be diluted twice as compared to a probe whose initial size was 2 kb. Riboprobes were safely stored at -20°C for years.

III. Plant culture and tissue array preparation

III.1. Plant material and culture conditions

The same wild-type *Arabidopsis thaliana* ecotype (Wassilewskija, Ws) and similar culture conditions (115 $\mu mol.m^{-2}.sec^{-1}$ continuous light using 25% Osram 58W Fluora and 75% lumilux cool daylight 58W tubular fluorescent lamps, respectively; 22°C; 75% relative humidity) as used for the tissue-specific transcriptomic reference study (Belmonte *et al.,* 2013) was primarily used. For specificity studies, *atprx36* T-DNA knock out insertion line [per36-1; SAIL_194_G03; (Kunieda *et al.,* 2013)] and its corresponding *A. thaliana* wild type background (Columbia 0, Col-0) were cultivated in the same conditions.

III.2. Tissue fixation and Paraplast infiltration

RNase-free conditions were strictly observed for all steps. In order to cover the whole kinetics of seed development, 39, 40, 43 and 45-day old plants were sampled each in three batches (siliques from the top, medium and bottom of the floral stem, the youngest siliques being at the top). In each case, dozens of whole siliques were harvested by cutting the pedicel with a razor blade and rapidly fixed in 50 ml Falcon tubes containing 20-30 ml of FAA (10% Formalin (37% formaldehyde solution, Sigma-Aldrich); 50% ethyl alcohol; 5% acetic acid; 35% DEPC-treated water). It is also possible to use dedicated embedding cassettes or other tubes, but we find the 50 ml tubes to be the most convenient to pour and fill the solutions while accommodating large quantities of samples. 20-30 tubes can easily be accommodated in a single experiment allowing to process thousands of samples. The fixative infiltration was achieved by 5 cycles of vacuum infiltration (1 min) / vacuum release in a vacuum chamber. The infiltrated samples were finally incubated for 6-16 h at 4°C in FAA and rinsed 4 times in 50% ethanol in DEPC water for 10 min, each. At this step, the samples can be processed directly or can be stored safely for weeks-to-months at 4°C in 50% ethanol. No obvious ISH difference was observed between the samples with different incubation times, and the final protocol corresponded to a harvest at 8 AM and fixation duration of 6 h. The infiltration protocol was adapted from (Burlat *et al.,* 2004) with a progressive dehydration in a DEPC water / absolute ethanol / tert-butanol gradient series with following proportions (50/40/10; 30/50/20; 15/50/35; 0/45/55 with 0.025% erythrosine solution to temporarily stain the depigmentated samples; 0/25/75, for 1 h each at room temperature (above the 25°C fusion point of tert-butanol) followed by overnight incubation in pure tert-butanol. The next day,

the tert-butanol solution was renewed for 1 h, then half of the tert-butanol was replaced by melted Paraplast Plus and the tubes were incubated in a dedicated oven at 60°C positioned under a hood for 5 h. The mixture was carefully poured and replaced by tert-butanol/ Paraplast Plus 50/50 pre-mix and incubated overnight at 60°C. From the next day and for the 2-3 following days, pure melted Paraplast Plus was changed once or twice a day (*i.e.* in the morning and/or in the evening) in order to have at least 4 changes of pure Paraplast Plus over this period. Caps must be removed during all these incubations in order to allow evaporation of remaining tert-butanol. At this stage, it is possible to directly perform tissue embedding or to store tubes with caps at 4°C. In this last case, on restart, the tubes have to be incubated for a day at 60°C before being able to access the samples.

III.3. Paraplast Plus embedding and tissue array preparation

Our embedding strategy was to concentrate numerous siliques at different developmental stages within single blocs constituting the so-called tissue arrays. The goal was to simultaneously and homogeneously cut longitudinal sections of numerous siliques at different developmental stages in order to optimize the *in situ* hybridization throughput. We used metallic embedding base molds (38 × 25 × 12 mm, Tissue-tek 4133; Delta Microscopies, Mauressac, France) that presented the advantage to have a flat base allowing to dispose a large number of thin samples (siliques) within a wide surface covering most of the area of a microscopy slide and to be adapted to standard polystyrene embedding rings (simport M460; Ref 040272, Dutscher SAS, Brumath, France) to later hold on the microtome arm. Large slide warmer (64 × 20 cm, Labscientific XH-2001; Delta Microscopies, Mauressac, France) was set to 62°C and protected with foil. On one side, metallic base molds were filled with melted Paraplast Plus and on the other side, 120 × 120 × 17 mm square Petri dishes were filled with siliques in melted Paraplast Plus (the tubes were directly poured in the Petri dishes). Metallic spatula and tweezers were kept warm on the slide warmer and sequentially used to dispose the siliques in the molds. Up to 90 siliques at different developmental stages containing all together about 1,000 developing seeds could be concentrated in a single tissue array (**Supplementary Figure S1b**). The filled tissue arrays were each covered by a white embedding ring with written reference/number and the whole system was gently moved to a frozen metallic plate to secure the position of the samples. The Paraplast Plus solidification and the fixation of plastic ring with the metallic mold were achieved after about 15 min at room temperature followed by cooling at 4°C for at least 1 h. The samples could then be separated from the base mold for direct microtomy or for storage at 4°C for years.

IV. Medium-throughput *in situ* hybridization conditions

IV.1. RNase-free conditions

The respect of RNase-free conditions, whenever reasonably possible, is crucial for the success of *in situ* hybridization. All glassware (except microscopy slides) and metallic materials were rinsed with distilled water, wrapped in aluminium foil and baked at 180°C for 8 h (Sambrook *et al.,* 1989). Non-disposable plastic material (*e.g.* tweezers, magnetic bars, Polypropylene staining racks for 20 slides) were washed for 20 min in 0.2N NaOH and rinsed 3 times in DEPC-treated water before drying overnight at 37°C in RNase-free glassware. RNase-free glassware and plastic material were properly

stored at room temperature in a dedicated cabinet for weeks-months. All disposable material was RNase free.

Water means either DEPC-treated water (Sambrook *et al.,* 1989) prepared in 5 l Schott bottle using red caps that can be safely baked at 180°C, or fresh milliQ water contained in RNase-free bottles.

Solvents and chemicals were dedicated for this type of experiment and stored separately. Stock solutions and concentrated buffers were prepared in RNase-free conditions and stored either at -20°C for small aliquots or at 4°C for bottles.

IV.2. Material, chemicals and solutions

IV.2.1. Bench material and large material

- Classical rotary microtome. We used a Seit 1872 Jung AG Microtome with tissue-tek A35 disposable microtome blades (Delta Microscopies, Mauressac, France).
- At least three magnetic stirrers enabling stirring of up to 2 l solutions
- 2 water baths (37°C and 65°C)
- Large slide warmer (64 × 20 cm, Labscientific XH-2001; Delta Microscopies, Mauressac, France). It can easily accommodate 40 slides used in one ISH experiment.
- Hybridization oven (interior 400 × 330 × 400 mm) with accurate temperature setting (Binder BF53 incubator; VWR International S.A.S., Fontenay-sous-Bois, France)
- Nanozoomer HT slide scanner (Hamamatsu, TRI imaging platform facilities, Toulouse, France)

IV.2.2. Specific glassware, plastic material and other materials

- Classical cleaned ready-to-use 76 × 26 mm Starfrost microscopy slides (not RNase free, use clean new boxes properly stored) (Dutscher SAS, Brumath, France)
- Cover slips (25 x 60 mm, Dutscher SAS, Brumath, France)
- Glass staining jars SPC (115 × 73 × 74 mm, Rogo-sampaic; Ref 068506 Dutscher SAS, Brumath, France). This material is relatively inexpensive and stocks of at least 20-30 jars are necessary.
- Polypropylene staining racks for 20 slides (Kartell polypropylene staining racks 354; Ref 391058 Dutscher SAS, Brumath, France). This material is inexpensive and stocks of at least 10-20 jars are convenient.
- Sterile Thermo Scientific™ Nunc™ BioAssay square dishes (245 × 245 × 25 mm; Ref 055253 Dutscher SAS, Brumath, France)
- Schott bottles of various volumes (0.5 l to 5 l) with red caps supporting heating at 180°C.
- Benchkote (Whatman 2300-599)

IV.2.3. Solvents and chemicals

- Acetone (analytical reagent grade, VWR chemicals), Xylene (Laboratory reagent grade, Fisher scientific), absolute ethanol (Normapur,VWR chemicals)
- Dimethylformamide (DMF; Analytical grade, Fisher scientific)
- Sodium acetate (Sigma-Aldrich S2889)
- Sodium carbonate (Na_2CO_3, Sigma-Aldrich 71345)
- Sodium bicarbonate ($NaHCO_3$, Sigma-Aldrich S4019)
- Formalin (37% formaldehyde solution, Sigma-Aldrich F1635)
- Diethyl pyrocarbonate (DEPC; Sigma-Aldrich D5758)
- 3-(Aminopropyl)triethoxysilane (Sigma-Aldrich A3648)
- Tris base (ultrapure, Euromedex, Mundolsheim, France)
- EDTA (Molecular biology grade, Euromedex, Mundolsheim, France)
- NaCl (Chemically pure grade, Euromedex, Mundolsheim, France)
- Proteinase K (Sigma-Aldrich P2308)
- Glycine (Molecular biology grade, Euromedex, Mundolsheim, France)
- Sodium phosphate monobasic NaH_2PO_4 (Sigma-Aldrich S3139)
- Sodium phosphate dibasic Na_2HPO_4 (Sigma-Aldrich S3264)
- Potassium phosphate dibasic KH_2PO_4 (Sigma-Aldrich 60353)
- Sodium citrate (citric acid, trisodium salt, dihydrate interchim CAS#6132-04-3)
- RNase A (Sigma-Aldrich R4875)
- Deionised formamide (Sigma Aldrich F9037)
- Formamide (Sigma-Aldrich F7503)
- Dextran sulfate (Sigma-Aldrich D8906)
- Yeast RNA (Roche 10109223001)
- BSA (Fraction V, Euromedex, Mundolsheim, France)
- Anti-digoxigenin-AP Fab fragments (Roche 11093274910)
- Nitro blue tetrazolium (Kalys N1411-1; Kalys S.A., Bernin, France)
- X-Phos *p*-Toluidine salt (BCIP *p*-Toluidine salt; Kalys X1413-1; Kalys S.A., Bernin, France)
- Eukitt® quick-hardening mounting medium (Sigma-Aldrich 03989)

IV.2.4. Buffers and solutions

Buffers were prepared in RNase-free liquid cylinders, with DEPC-treated water using normal pH meter electrode cleaned with DEPC-treated water and were stocked at 4°C for months/years in RNase-free Schott bottles, following autoclaving.

IV.2.4.1. Tris-EDTA (100 mM Tris-HCl pH 8.0, 50 mM EDTA pH 8.0) for proteinase K

Prepare 10X separated stock solutions: 1 M Tris-HCl pH 8.0 and 0.5 M EDTA pH 8.0 (40 ml each are necessary for one 40 slide ISH experiment):

IV.2.4.2. 1X Phosphate buffer (main buffer for ISH)

Prepare 10X stock solution (260 ml necessary for one 40 slide ISH experiment):

- 112 ml 1 M Na_2HPO_4 (70 mM final concentration)
- 48 ml 1 M NaH_2PO_4 (30 mM final concentration)
- 121.6 g NaCl (1.3 M final concentration)
- About 1.2 l DEPC water
- Adjust pH 7.0 and complete volume to 1.6 l with DEPC water.

IV.2.4.3. 0.2 % (w/v) glycine in 1X phosphate buffer

Prepare 1X stock solution (200 ml necessary for one 40 slide ISH experiment)

IV.2.4.4. Hybridization buffer stock solutions

Prepare 10X salts and aliquot in microtubes at -20°C:

- 30 ml of 5 M NaCl (3.0 M final concentration)
- 5 ml of 1 M Tris-HCl pH 6.8 (0.1 M final concentration)
- 5 ml of 1 M sodium phosphate pH 6.8 (0.1 M final concentration)
- 5 ml of 0.5 M EDTA (50 mM final concentration)
- DEPC water up to 50 ml

Yeast RNA should be deproteinated by phenol/chloroform extraction (Sambrook *et al.,* 1989) and diluted to 10 $mg.ml^{-1}$. Aliquots are stored in microtubes at -20°C.

50% (w/v) dextran sulfate solution is prepared in DEPC water. Solubilisation is tedious due to the viscosity. Aliquots are stored in microtubes at -20°C.

New batch of clean deionized formamide is directly aliquoted in microtubes and stored at -20°C.

IV.2.4.5. TBS-EDTA (10 mM Tris-HCl pH 7.5, 500 mM NaCl, 1 mM EDTA) for RNase A

Prepare 1X stock solution (400 ml necessary for one 40 slide ISH experiment)

IV.2.4.6. 20X SSC (for stringent post-hybridization washing)

Mix 140.24 g NaCl and 70.56 g sodium citrate in about 600 ml DEPC water. Adjust pH to 7.0 and fill with DEPC water up to 800 ml (about 272 ml necessary for one 40 slide ISH experiment)

IV.2.4.7. TTBS (100 mM Tris-HCl pH 7.5, 150 mM NaCl, 0.3% (w/v) Triton X100) for immunodetection

Prepare 10X solution without Triton X100 and store at 4°C (160 ml necessary for one 40 slide ISH experiment: mix extemporaneously 4.8 g Triton X100 with 160 ml 10X TTBS up to 1600 ml fresh milliQ water)

IV.2.4.8. TBS-$MgCl_2$ (100 mM Tris-HCl pH 9.5, 100 mM NaCl, 10 mM $MgCl_2$) for alkaline phosphatase reaction

Prepare 10X separated stock solutions of 1 M TBS_{1000} pH 9.5 and 100 mM $MgCl_2$ (100 ml each are necessary for one 40 slide ISH experiment)

IV.2.4.9. BCIP and NBT stock solutions

Dissolve 1 g BCIP (Kalys X1413-1) in DMF up to 20 ml in a 50 ml Falcon tube to get a 50 $mg.ml^{-1}$ stock solution stored at -20°C

Prepare 20 ml of 70% (v/v) DMF in fresh milliQ water and dissolve 1 g NBT (Kalys N1411-1) in 70% DMF up to 20 ml in a 50 ml Falcon tube to get a 50 $mg.ml^{-1}$ stock solution stored at -20°C.

IV.2.10. Slide coating

100 Starfrost microscopy slides (2 packs of 50) were carefully disposed with gloves in 5 polypropylene staining racks for 20 slides. Each rack was dipped for 5 min in 200 ml of fresh 2% (v/v) 3-aminopropyltriethoxysilane solution in acetone within a glass staining jar disposed under a fume hood. Each rack was allowed to dry for 15 min under the hood on a clean kimwipe and then sequentially washed in three glass staining jars each containing 200 ml of DEPC-treated water. The same solutions were used for sequential processing of the 5 racks. Racks were piled in 2 l glass beakers for drying overnight at 37°C and for storage for weeks/months in a clean dedicated place at room temperature.

V. Medium-throughput *in situ* hybridization protocol

At this stage, all material is available to perform an ISH experiment. Starting on Monday (day 1), the results will be available on Thursday (day) 4 morning for 40 slides each corresponding to one probe hybridized with serial sections of tissue arrays. The basis of this protocol comes from (Jackson 1992) with first adaptations by (St-Pierre *et al.,* 1999, Burlat *et al.,* 2004, Mahroug *et al.,* 2006) and final simplification using a unique buffer for most of the steps and substituting the acetic anhydre/triethanolamine tedious charge equilibration step by DEPC a more simple carbethoxylation step, inspired by (Brewer *et al.,* 2006).

V.1. Microtomy (few hours microtomy on day 1 and drying until day 2 morning)

40 slides were annotated with a pencil (do not use a pen since ink disappears with solvent), disposed as 2 lines of 20 on a slide warmer set to 35°C and carefully covered with 2-3 ml of DEPC-treated water previously degassed for at least 1 h in a vacuum chamber. Serial sections (10 µm thick) of tissue arrays were cut with a rotary microtome. Ribbons of sections were positioned on a large kimwipe and sections were carefully separated with needles and paint brush, and individually floated to spread in the same orientation and position on each slides. This careful positioning of the sections is important to facilitate medium-throughput during final comparative analysis of the results on the same exact tissues with different probes. For a typical ISH experiment we managed to float 2-3 sections from 2-3 different tissue arrays on each slides, all together representing about 1,000 developing seeds (**Supplementary Figure S1**). Given the diameter of the siliques and the thickness of the sections, tissue arrays could provide 20-30 longitudinal sections of young siliques and 30-40 longitudinal sections of mature siliques. Since each slide will correspond to one probe, the future probe distribution along the 40 slides was organized in order to allow the best morphology comparison

for a selection of probes of interest. Typically, antisense and sense probes or two antisense probes to be compared will be placed in subsequent slides corresponding to the same tissue array. When slides were entirely filled with sections, the excess water was carefully removed with a pipette and the slides were allowed to dry overnight at 35°C on the slide warmer for section adherence.

V.2. Paraplast Plus removal and prehybridization (day 2 morning)

Dispose 9 glass staining jar under a fume hood for the sequential deparaffinization/rehydration series with 200 ml of each solution as followed:

xylene (2 × 15 min), 100% ethanol (2 × 5 min), 95% ethanol, 70% ethanol, 50% ethanol (5 min each), DEPC-water (2 × 5 min). Note that the pink erythrosine colour of the samples will be solubilised in the aqueous baths. The same bath series is used for both 20 slide-racks with a 15 min delay. Keep this bath series clean for re-use during the dehydration series at the end of the prehybridization.

During this series prepare 2 glass staining jars each prewarmed with 200 ml TE at 37°C in the hybridization oven. At the end of the rehydration series, incubate the first 20 slide-rack for 15 min and the second one for 10 min in order to equilibrate the slides and to set a 5 min delay between each racks for the following steps. Quickly add and mix concentrated proteinase K to have a 2 $\mu g.ml^{-1}$ final concentration in each jar and digest for 30 min at 37°C with the 5 min delay between the two 20 slide-racks.

During this step, prepare 2.6 l of 1X PBS and organize 14 glass staining jars for the next prehybridization steps to be directly proceeded at the end of the digestion, keeping the 5 min delay between the two racks all over the process (the same jar/solution will be used for each step equal to or below 5 min and two jars will be doubled for the post fixation steps and the carbethoxylation steps exceeding 5 min):

- Washing in PBS (200 ml, 15 sec)
- Blocking proteinase K in PBS-2% glycine (200 ml, 2 min)
- Washing in PBS (200 ml, 1 min)
- Washing in PBS (200 ml, 1 min)
- Post fixation in 10% formalin (3.7% formaldehyde) in PBS (200 ml, 10 min, fume hood)
- Washing in PBS (200 ml, 5 min)
- Washing in PBS (200 ml, 5 min)
- Carbethoxylation in 0.1% (v/v) DEPC in PBS (200 ml, 15 min, fume hood)
- Carbethoxylation in 0.1% (v/v) DEPC in PBS (200 ml, 15 min, fume hood)
  Note that DEPC will sediment in the jars during the incubation if no care is taken. To avoid stirring during the incubation we set the convenient following condition: Prepare 800 ml of 0.1% DEPC in PBS in a 1 l Schott bottle. Thoroughly shake before the distribution of 200ml in each of the 4 jars just before use, respectively. During the 15 min incubation periods of the 20-slide racks in the jars, invert the racks by 180° every five min. Finally when these steps are

finished put back the 800 ml in the bottle and degrade the DEPC by autoclaving before throwing it away.

- Washing in PBS (200 ml, 5 min)
- Washing in PBS (200 ml, 5 min)

At this stage the slides are dehydrated by incubation in the baths kept under the fume hood as follows: 50% ethanol, 70% ethanol, 95% ethanol, 100% ethanol (5 min each)

Make sure not to re-use the 100% ethanol bath that followed the xylene bath since it is more saturated in solubilised paraffin. Slides are allowed to completely air dry at least 1 h, by storing the 20 slide-racks on an RNase-free surface during the preparation of the following steps.

V.3. Hybridization (few hours to prepare and distribute the mix and overnight hybridization)

Prepare in a 50 ml Falcon tube about 6 ml of hybridization medium by carefully mixing aliquots of the following stock solutions stored at -20°C (these should be melted on ice a few hours before):

- 1,100 µl DEPC water
- 600 µl 10X salts (thoroughly vortex the melted aliquot until no precipitate is visible)
- 2,400 µl deionized formamide
- 600 µl 10 $mg.ml^{-1}$ yeast RNA
- 1,200 µl 50% dextran sulfate (slowly pipet and distribute since it is highly viscous; it can be heated to 50°C to reduce the viscosity)
- 6 µl RNasine (Promega)

Distribute 137.8 µl of hybridization buffer in a series of 40 microtubes (slowly pipet and distribute since it is relatively viscous) and add 2.24 µl of probes in order to obtain a concentration of 400 $ng.kb^{-1}.\mu l^{-1}$ hybridization buffer (probe stock solutions that were set to 25 $ng.kb^{-1}.\mu l^{-1}$ and stored at -20°C have to be melted on ice a few h before, and should be vortexed and spinned down before use).

Prepare 4 Nunc square dishes each with a piece of clean pre-cut benchkote soaked with a mix of 50% formamide/2X SSC (about 12-16 ml/dish). Wearing clean gloves, carefully flatten the fully soaked benchkote and eliminate the excess solution. Take the slides from the 20 slide-racks with tweezers and dispose 10 slides per dish, carefully avoiding any contact between them and pressing with the tweezers on the white frame to secure the slide position on the humid benchkote. Distribute 135-140 µl of the probe mixtures as a line in the middle of the corresponding slides avoiding air bubbles. Carefully cover each slide with a 25 × 60 mm cover slip. Make sure that the solution mixture does not escape to the white frame before proceeding to the hybridization. Make a pile of the 4 covered Nunc square dishes and totally wrap the dishes together with Saran to maintain a saturated atmosphere during the overnight (at least 16 h) hybridization at 50°C in the hybridization oven.

Competitive inhibition experiments were performed similarly except that the hybridization medium contained 400 $ng.kb^{-1}.\mu l^{-1}$ of digoxigenin-labelled antisense probe together with 4,000 $ng.kb^{-1}.\mu l^{-1}$ of unlabelled competitive antisense probe (113.1 µl of hybridization buffer mixed with 2.24 µl dig-labelled probe and 22.4µl unlabelled probe).

V.4. Stringent washing (day 3 morning)

Three glass staining jars were prewarmed, one with 200 ml 2X SSC, two with 200 ml TBS-EDTA, at 37°C in a water bath. The pile of covered Nunc square dishes was removed from the hybridization oven and the slides were individually taken with clean gloves and tweezers (maintaining a 90° angle between the tweezers and the slide) and gently dip in the 2X SSC bath until the cover slip fall by itself. The slides were put back in the 20-slide racks (preserved empty on a RNase free surface during the hybridization period) and were not allowed to dry by immersing the rack in the 2X SSC solution every 5-10 slides. RNase A was added to 50 $\mu g.ml^{-1}$ to each TBS-EDTA baths and both racks were incubated in parallel during 30 min at 37°C (no need to have the 5 min delay between the two racks as during the prehybridization).

Prepare three 2 l beakers containing each a magnetic bar and a glass slide rack (102 × 80 × 24 mm, with 10 slide positions) that fits within the glass staining jars. This home-made system used this type of classical glass rack only to allow magnetic stirring in its middle while accommodating the two 20-slide racks on its top during subsequent washing steps. The three beakers were filled with 1.5 l of 2X, 1X and 0.1X SSC, respectively. Each washing step lasted 1 h under gentle magnetic stirring inverting the two racks after 30 min to ensure homogeneous washing. The 2X and 1X washing were performed at room temperature while the 0.1X was performed at 65°C. To achieve this goal we put a plastic water bath set to 65°C on top of a magnetic stirrer and we immersed the 2 l beaker system in the bath. These stringent washing conditions are necessary for hybridization specificity.

V.5. Immunological detection of hybridized probes (day 3 afternoon)

The two 20-slide racks containing the slides were equilibrated in parallel in 2 glass staining jars each containing 200 ml TTBS for 10 min and the slides were blocked in 2 glass staining jars, each containing 200 ml 2% (w/v) BSA in TTBS for 30 min. Slides were further individually incubated with 140µl anti-digoxigenin-AP Fab fragments diluted 1:200 in 1% (w/v) BSA in TTBS under cover slips for 2 h at room temperature, using the same Nunc square dishes and benchkote (additionally soaked with TTBS) as for the hybridization step. The same tweezers and gesture as those used following the hybridization step were used to carefully remove the cover slips, using TTBS solution contained in a glass staining jar. This step is critical to avoid destroying the sections. The two 20-slide racks were further washed twice 15 min in TTBS and twice 10min in TBS-$MgCl_2$ using 200 ml solution in glass staining jars for each step/rack.

V.6. Colour development of AP reaction (overnight day 4 morning)

In order to save substrate, we used 2 rectangular glass staining jars (inside 3" × 25/16" × 13/16"), each accommodating 20 slides back to back within their 10 slide positions. The two jars were each filled with 80 ml of TBS-$MgCl_2$ to which was added 250 µl BCIP and 500 µl NBT stock solutions. The

filled and covered jars were wrapped in aluminium foil and the slides were incubated in the chromogenic alkaline phosphatase substrate overnight (at least 16 h) at room temperature. Slides were put back in the 2 polypropylene 20-slide staining racks and the reaction was stopped by washing 8 times in 200 ml distilled water (no need of RNase-free water at this step). The back and side of the slides was carefully cleaned with a kimwipe and slides were totally dried under a fume hood on a slide warmer set to 40-50°C.

V.7. Slide permanent mounting and scanning (day 4)

Three droplets of Eukitt were longitudinally disposed along each slide avoiding air bubbles and directly covered by a cover slip. Before the Eukitt started to dry, the cover slips were carefully adjusted; excess air bubbles (if any) were removed by gently pushing them away with two pipette tips. After 1-2 h on the slide warmer set to 40-50°C under the fume hood allowing solvent evaporation, the slides were ready to be observed with bright-field microscopy or to be scanned at high-throughput using a nanozoomer HT slide scanner (Hamamatsu) that could accommodate up to 210 slides. We routinely scanned all slides at × 20 focus (= 0.46 µm per pixel on a single z plan) and these scans were directly used to prepare Figures. In some instance, we also scanned at higher resolution (× 40 focus on several z plans) in order to reach better resolution.

V.8. Result analysis and figure mounting

The NDP view freeware (Hamamatsu) allowed easy visualization files despite their large size (a typical 20x image covering all the slide corresponded to 300-400 Mb). The rotation and synchronization modes of NDP view were routinely used to simultaneously compare 2-8 slides on the same exact zone using 1 or 2 computer screens. This mode of observation greatly increased the image analysis throughput allowing to directly compare 2-8 probes (either antisense or sense) on serial sections of the exact same seed samples. Images were extracted from NDP view to assemble Figures using Corel Photopaint. All together tens of thousands of individual seed sections were viewed on the computer screen, and each image displayed on the Figures was representative of numerous seeds observed in at least 3 experimental repeats.

VI. Integration of ISH and tissue-specific transcriptomic results

One Figure was assembled for each gene studied by ISH. In each Figure, we compared the screen copy of the individual tissue-specific transcriptomic maps and relative heatmap scale available through seed eFP browser at http://bar.utoronto.ca/efp/cgi-bin/efpWeb.cgi?dataSource=Seed (Winter *et al.,* 2007) for 5 selected developmental stages, with corresponding ISH antisense and sense images directly extracted from NDP view. Finally, we constructed using Corel Photopaint a new detailed expression map for each gene based on our ISH results, with a red/orange/white colour code (red, strong ISH signals; orange, moderate ISH signals; white, no ISH signal), and using the original detailed cartoon from Belmonte *et al*. (2013) available at Seedgenenetwork (http://estdb.biology.ucla.edu/ seed/) as a basis for the drawing except for the bending cotyledons stage for which we drew our own cartoon. Note that the new ISH maps corresponded to the

observation of numerous seed sections from at least 3 experimental repeats and not only to the individual displayed ISH images.

The **Supplementary Table S1** was used to extract tissue-specific transcriptomic expression profiles from all the *AtPRXs* and the 39 genes studied in ISH. This data was gathered in single Microsoft Excel files (**Supplementary Table S4** for peroxidases and **Supplementary Table S5** for the 39 genes studied by ISH), keeping the original information about the maximum expression value and the ranking position of each gene. The individual tissue-specific transcriptomic values were framed in bold when either strong or moderate ISH signal was observed in the corresponding tissues according to **Supplementary Figures S4-S42.** The gene annotation was framed in red (strong ISH signal in at least one tissue), orange (moderate-to-low ISH signal in at least one tissue) or yellow (no ISH signal detected) following the similar colour code as in **Supplementary Figures S4-S42**.

The **Supplementary Table S1** was sequentially used to distribute, for each of 36 tissue-specific transcriptomic samples (all samples except the 6 whole seed samples), the 23,933 genes within 10 groups according to their transcriptomic expression value. The groups corresponded to expression values of 0-44 (below the detection limit of the transcriptomic study; 55-65% of the genes depending on the sample), 45-299 (below the arbitrary 300 cut-off used for our first selection of candidate genes), 1000-1999, 2000-2999, 3000-3999, 4000-4999, 5000-9999, 10000-19999, 20000-max. In order to fully integrate our ISH results with the whole tissue-specific transcriptomic data, the abbreviation of the 37 genes of the 39 genes studied by ISH that were present on the array (all 39 genes except *AtPRX13* and *AtPRX32*) was positioned on the individual histograms on the top of the range corresponding to their tissue-specific transcriptomic value (**Figure 3; Supplementary Figures S46-S51**). In order to summarize the ISH results, we kept the same red/orange/white colour code described above. Less genes were studied in ISH for the preglobular stage which was under-represented on the tissue arrays. We finally calculated within colour-coded double arrows positioned on the top of the histograms, the sum of genes for which ISH could be sensitive enough to various degrees for each tissue/developmental stage.

Supplementary Methods references:

Albenne, C., Canut, H. and Jamet, E. (2013) Plant cell wall proteomics: the leadership of *Arabidopsis thaliana*. *Front. Plant Sci.*, **4**, 111.

Altschul, S.F., Madden, T.L., Schaffer, A.A., Zhang, J.H., Zhang, Z., Miller, W. and Lipman, D.J. (1997) Gapped BLAST and PSI-BLAST: a new generation of protein database search programs. *Nucleic Acids Res.*, **25**, 3389-3402.

Belmonte, M.F., Kirkbride, R.C., Stone, S.L., Pelletier, J.M., Bui, A.Q., Yeung, E.C., Hashimoto, M., Fei, J., Harada, M., Munoz, M.D., Le, B.H., Drews, G.N., Brady, S.M., Goldberg, R.B. and Harada, J.J. (2013) Comprehensive developmental profiles of gene activity in regions and subregions of the Arabidopsis seed. *Proc. Natl. Acad. Sci. U. S. A.*, **110**, E435-E444.

Brewer, P.B., Heisler, M.G., Hejatko, J., Friml, J. and Benkova, E. (2006) *In situ* hybridization for mRNA detection in Arabidopsis tissue sections. *Nat. Protoc.*, **1**, 1462-1467.

Burlat, V., Oudin, A., Courtois, M., Rideau, M. and St-Pierre, B. (2004) Co-expression of three MEP pathway genes and geraniol 10-hydroxylase in internal phloem parenchyma of *Catharanthus roseus* implicates multicellular translocation of intermediates during the biosynthesis of monoterpene indole alkaloids and isoprenoid-derived primary metabolites. *Plant J.*, **38**, 131-141.

Fawal, N., Li, Q., Savelli, B., Brette, M., Passaia, G., Fabre, M., Mathe, C. and Dunand, C. (2013) PeroxiBase: a database for large-scale evolutionary analysis of peroxidases. *Nucleic Acids Res.*, **41**, D441-D444.

Francoz, E., Ranocha, P., Nguyen-Kim, H., Jamet, E., Burlat, V. and Dunand, C. (2015) Roles of cell wall peroxidases in plant development. *Phytochemistry*, **112**, 15-21.

Irshad, M., Canut, H., Borderies, G., Pont-Lezica, R. and Jamet, E. (2008) A new picture of cell wall protein dynamics in elongating cells of *Arabidopsis thaliana*: Confirmed actors and newcomers. *BMC Plant Biol.*, **8**, 94.

Jackson, D. (1992) *In situ* hybridisation in plants. . In *Molecular Plant Pathology: a Practical Approach* (Gurr, S.J., McPherson, M.J. and Bowles, D.J. eds). Oxford, UK: IRL Press at Oxford University, pp. 163-174.

Koua, D., Cerutti, L., Falquet, L., Sigrist, C.J.A., Theiler, G., Hulo, N. and Dunand, C. (2009) PeroxiBase: a database with new tools for peroxidase family classification. *Nucleic Acids Res.*, **37**, D261-D266.

Kunieda, T., Shimada, T., Kondo, M., Nishimura, M., Nishitani, K. and Hara-Nishimura, I. (2013) Spatiotemporal secretion of PEROXIDASE36 is required for seed coat mucilage extrusion in Arabidopsis. *Plant Cell*, **25**, 1355-1367.

Le, B.H., Cheng, C., Bui, A.Q., Wagmaister, J.A., Henry, K.F., Pelletier, J., Kwong, L., Belmonte, M., Kirkbride, R., Horvath, S., Drews, G.N., Fischer, R.L., Okamuro, J.K., Harada, J.J. and Goldberg, R.B. (2010) Global analysis of gene activity during Arabidopsis seed development and identification of seed-specific transcription factors. *Proc. Natl. Acad. Sci. U. S. A.*, **107**, 8063-8070.

Mahroug, S., Courdavault, V., Thiersault, M., St-Pierre, B. and Burlat, V. (2006) Epidermis is a pivotal site of at least four secondary metabolic pathways in *Catharanthus roseus* aerial organs. *Planta*, **223**, 1191-1200.

Sambrook, J., Fritsh, E. and Maniatis, T. eds (1989) Molecular cloning. A laboratory manual Cold Spring Harbor: Laboratory Press.

Schenborn, E.T. and Mierendorf, R.C. (1985) A novel transcription property of SP6 and T7 RNA-polymerases: Dependence on template structure *Nucleic Acids Res.*, **13**, 6223-6236.

Schneider, C.A., Rasband, W.S. and Eliceiri, K.W. (2012) NIH Image to ImageJ: 25 years of image analysis. *Nat. Methods*, **9**, 671-675.

Seki, M., Carninci, P., Nishiyama, Y., Hayashizaki, Y. and Shinozaki, K. (1998) High-efficiency cloning of Arabidopsis full-length cDNA by biotinylated CAP trapper. *Plant J.*, **15**, 707-720.

Seki, M., Narusaka, M., Kamiya, A., Ishida, J., Satou, M., Sakurai, T., Nakajima, M., Enju, A., Akiyama, K., Oono, Y., Muramatsu, M., Hayashizaki, Y., Kawai, J., Carninci, P., Itoh, M., Ishii, Y., Arakawa, T., Shibata, K., Shinagawa, A. and Shinozaki, K. (2002) Functional annotation of a full-length Arabidopsis cDNA collection. *Science*, **296**, 141-145.

Simon, R., Mirlacher, M. and Sauter, G. (2004) Tissue microarrays. *Biotechniques*, **36**, 98-105.

St-Pierre, B., Vazquez-Flota, F.A. and De Luca, V. (1999) Multicellular compartmentation of *Catharanthus roseus* alkaloid biosynthesis predicts intercellular translocation of a pathway intermediate. *Plant Cell*, **11**, 887-900.

Winter, D., Vinegar, B., Nahal, H., Ammar, R., Wilson, G.V. and Provart, N.J. (2007) An "Electronic Fluorescent Pictograph" browser for exploring and analyzing large-scale biological data sets. *PLoS One*, **2**, e718.

Supplementary Table S9: List of plasmids used in this study and conditions for riboprobe *in vitro* transcription. The plasmids were obtained from the RIKEN genomic resource center (http://www.brc.riken.jp/lab/epd/catalog/cdnaclone.html) or directly cloned in pGEM-T Easy. The different columns give various information on the clones with hypertext links, and include the conditions used for restriction *in vitro* transcription of antisense (AS) in green and sense (S) in red riboprobes (restriction enzyme for linearization, RNA polymerase for transcription, probe length and time of alkaline hydrolysis to chop the probes to an average of 400b are also provided).

							Antisense (AS) riboprobes				Sense (S) riboprobes			
Locus identifier	Annotation	GenBank	Size (bp)	CDS (bp position)	Selected RIKEN Resource #	Corresponding cDNA clone name (see Supplementary Methods Fig. 1 for the multiple cloning site maps)	Restriction enzyme (* cut in cDNA)	RNA Pol	Probe length (b)	Hydrolysis time (min) 400b	Restriction enzyme (* cut in cDNA)	RNA Pol	Probe length (b)	Hydrolysis time (min) 400b
**At1g05260**	***AtPRX03***	AK227859	**1198**	**56-1036**	pda18567	RAFL14-42-O04	**EcoRI**	**T3**	**1297**	**15,72**	**Asp718**	**T7**	**1375**	**16,12**
**At1g68850**	***AtPRX11***	AK175248	**1262**	**71-1081**	pda13621	RAFL21-67-O04	**NotI**	**T3**	**1427**	**16,36**	**Asp718**	**T7**	**1439**	**16,41**
**At1g71695**	***AtPRX12***	BT000715	**1264**	**52-1128**	pda09996	RAFL09-89-E13	**EcoRI**	**T3**	**1375**	**16,12**	**BamHI**	**T7**	**1415**	**16,3**
**At1g77100**	***AtPRX13***	NM106360	**1011**	**1-1011**	**pGEMT-Easy**		**NcoI**	**SP6**	**1152**	**14,84**	**NdeI**	**T7**	**1145**	**14,79**
**At2g22420**	***AtPRX17***	BT004021	**1088**	**32-1021**	pda12861	RAFL15-19-M20	**EcoRI**	**T3**	**1187**	**15,07**	**Asp718**	**T7**	**1265**	**15,54**
**At2g37130**	***AtPRX21***	AY059933	**995**	**206-889**	pda02895	RAFL06-08-J02	**Asp718**	**T3**	**1112**	**14,55**	**EcolCRI**	**T7**	**1086**	**14,36**
**At2g38380**	***AtPRX22***	AK230233	**1953**	**814-1863**	pda20767	RAFL23-07-B04	**EcoRI**	**T3**	**2052**	**18,3**	**Asp718**	**T7**	**2130**	**18,46**
**At2g38390**	***AtPRX23***	AY099555	**1221**	**46-1095**	pda08364	RAFL09-42-N24	**EcoRI**	**T3**	**1332**	**15,9**	**BamHI**	**T7**	**1372**	**16,1**
**At3g32980**	***AtPRX32***	AY080608	**1208**	**45-1103**	pda07720	RAFL06-83-F21	**Asp718**	**T3**	**1325**	**15,87**	**EcolCRI**	**T7**	**1299**	**15,73**
**At3g50990**	***AtPRX36***	AK229843	**1272**	**21-1055**	pda20378	RAFL22-03-B11	**EcoRI**	**T3**	**1371**	**16,1**	**BamHI**	**T7**	**1409**	**16,28**
**At4g21960**	***AtPRX42***	AY059810	**1252**	**40-1032**	pda02336	RAFL04-16-O12	**Asp718**	**T3**	**1369**	**16,09**	**EcolCRI**	**T7**	**1343**	**15,96**
**At4g25980**	***AtPRX43***	NM118731	**1116**	**1-1116**	**pGEMT-Easy**		**NcoI**	**SP6**	**1248**	**15,44**	**NdeI**	**T7**	**1241**	**15,4**
**At4g31760**	***AtPRX46***	NM119325	**1065**	**1-1065**	**pGEMT-Easy**		**NcoI**	**SP6**	**1207**	**15,2**	**NdeI**	**T7**	**1200**	**15,15**
**At4g37520**	***AtPRX50***	AY062816	**1147**	**42-1031**	pda02873	RAFL06-08-C18	**XhoI**	**T3**	**1229**	**15,33**	**EcolCRI**	**T7**	**1238**	**15,38**
**At4g37530**	***AtPRX51***	AY070459	**1160**	**36-1025**	pda06253	RAFL09-41-F02	**EcoRI**	**T3**	**1271**	**15,57**	**Asp718**	**T7**	**1351**	**16**
**At5g06720**	***AtPRX53***	AY056186	**1233**	**76-1083**	pda04715	RAFL08-12-E19	**EcoRI**	**T3**	**1344**	**15,96**	**BamHI**	**T7**	**1384**	**16,16**
**At5g14130**	***AtPRX55***	AY057607	**1190**	**40-1032**	pda06095	RAFL09-35-C03	**NotI**	**T3**	**1371**	**16,1**	**Asp718**	**T7**	**1381**	**16,14**
**At5g15180**	***AtPRX56***	AY072172	**1191**	**43-1032**	pda05794	RAFL09-25-M23	**EcoRI**	**T3**	**1302**	**15,75**	**Asp718**	**T7**	**1382**	**16,15**
**At5g39580**	***AtPRX62***	BT004203	**1256**	**82-1041**	pda13156	RAFL16-02-G15	**NotI**	**T3**	**1421**	**16,33**	**BamHI**	**T7**	**1393**	**16,2**
**At5g42180**	***AtPRX64***	AY063962	**1235**	**71-1024**	pda03304	RAFL06-68-K16	**XhoI**	**T3**	**1317**	**15,82**	**EcolCRI**	**T7**	**1326**	**15,87**
**At5g51890**	***AtPRX66***	AY072121	**1222**	**67-1035**	pda06970	RAFL09-92-M15	**EcoRI**	**T3**	**1333**	**15,91**	**BamHI**	**T7**	**1373**	**16,11**
**At5g64100**	***AtPRX69***	AY093012	**1234**	**60-566**	pda07505	RAFL06-07-K07	**XhoI**	**T3**	**1316**	**15,82**	**EcoRI**	**T7**	**1169**	**14,95**
**At5g64120**	***AtPRX71***	AF428274	**1287**	**58-1044**	pda06662	RAFL09-69-I19	**EcoRI**	**T3**	**1398**	**16,22**	**BamHI**	**T7**	**1438**	**16,41**
**At5g66390**	***AtPRX72***	AK227717	**1205**	**56-1066**	pda18425	RAFL14-30-F18	**EcoRI**	**T3**	**1304**	**15,76**	**BamHI**	**T7**	**1342**	**15,95**
**At4g36700**	***cupin***	BT003976	**1817**	**31-1598**	pda12680	RAFL15-06-K01	**EcoRI**	**T3**	**1913**	**17,98**	**BamHI**	**T7**	**1954**	**18,07**
**At3g22640**	***PAP85***	AY058085	**1664**	**20-1480**	pda05847	RAFL09-31-G07	**EcoRI**	**T3**	**1775**	**17,61**				
**At2g05580**	***GRP***	BT008624	**1005**	**30-851**	pda13261	RAFL15-04-D20	**NotI**	**T3**	**1170**	**14,96**	**BamHI**	**T7**	**1142**	**14,77**
**At2g38530**	***LTP2***	AY059927	**608**	**55-411**	pda02841	RAFL06-07-J20	**XhoI**	**T3**	**690**	**9,55**	**EcolCRI**	**T7**	**699**	**9,72**
**At2g38540**	***LTP1***	AY049296	**634**	**65-421**	**pGEMT-Easy**		**SalI**	**T7**	**700**	**9,74**	**NcoI**	**SP6**	**700**	**9,74**
**At4g12910**	***SCPL20***	AY136365	**1840**	**208-1701**	pda09699	RAFL09-28-C19	**EcoRI**	**T3**	**1951**	**18,07**	**Asp718**	**T7**	**2031**	**18,25**
**At1g21310**	***EXT3***	NM101983	**1663**	**22-1317**	**pGEMT-Easy**		**NdeI**	**T7**	**1452**	**16,47**	**NcoI**	**SP6**	**1459**	**16,5**
**At1g19230**	***RBOHE***	3	**2878**	**janv-05**	**pGEMT-Easy**		**NcoI**	**SP6**	**2991**	**19,69**	**NdeI***	**T7**	**2291**	**18,76**
**At1g64060**	***RBOHF***	AK228418	**4141**	**238-1716**	pda19110	RAFL14-95-K20	**NotI**	**T3**	**4306**	**20,62**	**BamHI**	**T7**	**4278**	**20,6**
**At5g48100**	***LAC15***	BT002919	**1861**	**34-1731**	pda12625	RAFL15-04-D21	**EcoRI**	**T3**	**1960**	**18,09**	**BamHI**	**T7**	**1998**	**18,18**
**At4g11180**	***DIR12/DP1***	BT004016	**729**	**17-574**	pda13066	RAFL15-41-E09	**NotI**	**T3**	**894**	**12,56**	**BamHI**	**T7**	**866**	**12,23**
**At3g08030**	***DUF642***	AY072318	**1277**	**109-1206**	pda07290	RAFL05-13-A11	**XhoI***	**T3**	**1323**	**15,86**	**HindIII***	**T7**	**1073**	**14,25**
**At5g44340**	***TUB4***	AY035141	**1731**	**108-1442**	pda01095	RAFL05-09-F01	**XhoI**	**T3**	**1822**	**17,74**	**BamHI**	**T7**	**1210**	**15,21**
**At5g67360**	***SBT1.7***	AF360285	**2618**	**115-2388**	pda01306	RAFL05-16-L13	**Asp718**	**T3**	**2735**	**19,4**	**EcolCRI**	**T7**	**2709**	**19,37**
**At1g28290**	***AGP31***	AK229236	**1187**	**28-975**	pda19846	RAFL16-52-I05	**EcoRI**	**T3**	**1286**	**15,66**	**BamHI**	**T7**	**1324**	**15,86**

Supplementary Table S10: Primers and PCR information for the cloning in pGEM-T Easy of *RBOHE* , *EXT3* , *LTP1* , *AtPRX13* , *AtPRX43* and *AtPRX46*

Locus identifier	Annotation		Oligonucleotide primer sequence (5'-3')	Tm (°C)	Amplicon size (bp)
At1g19230	***RBOHE***	forward	ATGAAGTTATCGCCTCTGAGTT	60.7	2859
		reverse	TTAGAAATGCTCCTTATGGAACTC	61.3	
At1g21310	***EXT3***	forward	AAACCAAAAACAAAACAACCAATGG	67.0	1332
		reverse	CCGTGATAGAGATATCTAATAGTG	55.4	
At2g38540	***LTP1***	forward	CTTCATCATCCATCACTACACACATC	60.6	643
		reverse	ACTTAAACTTCAAGTACTCTGAAATTTCAT	58.9	
At1g77100	***AtPRX13***	forward	ATGAGCCGTAGAATACGAGAAG	61.2	1011
		reverse	CTAGTTTGTAGCACTGCATAAACG	61.6	
At4g25980	***AtPRX43***	forward	ATGTCTGTCTCTATTCCGGTCTTA	61.8	1116
		reverse	TCAATTGAAGGCCGAGCAAA	67.7	
At4g31760	***AtPRX46***	forward	ATGGCGTCCTCTTACAGAAT	60.2	1065
		reverse	CTAACTAGTAGTAGTAAATAATTTCACTTTAC	55.0	
